# Opening of the Diamondoid Cage upon Ionization Probed by Infrared Spectra of the Amantadine Cation Solvated by Ar, N_2_, and H_2_O

**DOI:** 10.1002/chem.202200577

**Published:** 2022-06-20

**Authors:** Martin Andreas Robert George, Otto Dopfer

**Affiliations:** ^1^ Institut für Optik und Atomare Physik Technische Universität Berlin Hardenbergstr. 36 10623 Berlin Germany

**Keywords:** amantadine, carbocation, diamondoids, IR spectroscopy, structure elucidation

## Abstract

Radical cations of diamondoids, a fundamental class of very stable cyclic hydrocarbon molecules, play an important role in their functionalization reactions and the chemistry of the interstellar medium. Herein, we characterize the structure, energy, and intermolecular interaction of clusters of the amantadine radical cation (Ama^+^, 1‐aminoadamantane) with solvent molecules of different interaction strength by infrared photodissociation (IRPD) spectroscopy of mass‐selected Ama^+^L_*n*_ clusters, with L=Ar (*n*≤3) and L=N_2_ and H_2_O (*n*=1), and dispersion‐corrected density functional theory calculations (B3LYP−D3/cc‐pVTZ). Three isomers of Ama^+^ generated by electron ionization are identified by the vibrational properties of their rather different NH_2_ groups. The ligands bind preferentially to the acidic NH_2_ protons, and the strength of the NH…L ionic H‐bonds are probed by the solvation‐induced red‐shifts in the NH stretch modes. The three Ama^+^ isomers include the most abundant canonical cage isomer (**I**) produced by vertical ionization, which is separated by appreciable barriers from two bicyclic distonic iminium ions obtained from cage‐opening (primary radical **II**) and subsequent 1,2 H‐shift (tertiary radical **III**), the latter of which is the global minimum on the Ama^+^ potential energy surface. The effect of solvation on the energetics of the potential energy profile revealed by the calculations is consistent with the observed relative abundance of the three isomers. Comparison to the adamantane cation indicates that substitution of H by the electron‐donating NH_2_ group substantially lowers the barriers for the isomerization reaction.

## Graphical abstract

**Cage‐opening upon ionization**: Three isomers of Ama^+^ (1‐aminoadamantane) are identified by IR spectroscopy ‐ the cage isomer (**I**) generated by vertical ionization and separated by substantial barriers from two bicyclic distonic iminium ions generated by cage opening (primary radical **II**) and subsequent 1,2 H shift (tertiary radical **III**). The potential energy profile for this reaction depends strongly on solvation and H→NH_2_ substitution.

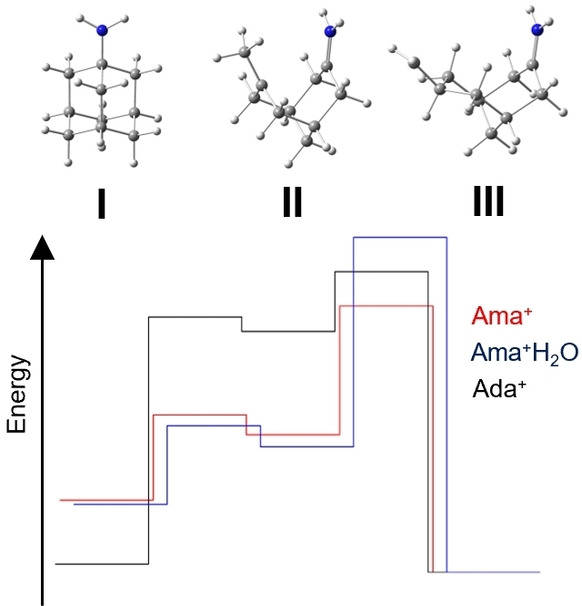

## Introduction

Amantadine (Ama, 1‐tricyclo[3.3.1.1^3,7^]decylamine, 1‐adamantylamine, 1‐aminoadamantane, C_16_H_15_NH_2_) is the amino derivative of adamantane (C_10_H_16_, Ada), the smallest diamondoid.^[1]^ Diamondoids are nm‐sized H‐passivated nanodiamonds.^[2]^ These rigid, stress‐free, and highly stable cycloalkanes form a fundamental class of saturated sp^3^‐hybridized hydrocarbon molecules. Due to their special properties, diamondoids and their derivatives have (potential) applications in a variety of disciplines, ranging from materials and polymer science, molecular electronics, medical sciences, and chemical synthesis to astrochemistry.^[3]^ For example, diamondoids and their radical cations are predicted to be abundant in the interstellar medium, and since several amines have been already detected,^[4]^ the presence of Ama and its cation is expected as well.[^3i^, ^5^] On the other hand, radical cations of diamondoids are frequently involved as intermediates in substitution reactions occurring in polar solvents.[^3e^, ^6^] Despite its high stability, functionalization of Ada and higher diamondoids can readily be carried out, making several derivatives accessible.[^3b^, ^7^]

One of the most important diamondoid derivatives is Ama, because it has widespread pharmaceutical applications as anti‐virus and anti‐Parkinson drug and thus is successfully marketed under several brand names, including Symmetrel® (Ama ⋅ HCl), Gocovri®, Symadine®, and Osmolex ER®.[^3f^, ^8^] Ama is effective in the prophylaxis and treatment of influenza A infections, because it prevents the virus from entering the host cell by blocking the ion channel.^[9]^ Due to drug resistance, however, its use is no longer recommended for influenza.^[10]^ Although the mechanism of action as anti‐Parkinson drug is not yet fully understood, it increases the release of dopamine from the nerve endings of brain cells and stimulates the noradrenaline response.^[11]^ In addition, Ama has NMDA receptor antagonistic effects^[12]^ and is discussed for treatment of other diseases such as multiple sclerosis, depression, and cocaine addiction.^[13]^ In 2013, the methylated derivative (memantine, Namenda®) was among the top 100 drugs sold worldwide, with sales of more than 10^9^ US$.^[14]^

In Ama (*C*_s_), the H atom of one of the four CH groups of Ada (*T*_d_) is replaced by an NH_2_ group, thus preserving most of the special chemical properties of diamond‐like structures.[^12b^, ^15^] The vibrational, electronic, and geometric properties of Ama have been determined by Raman, infrared (IR), and electron momentum spectroscopy.^[16]^ In addition, the interaction between Ama and DNA was investigated by Raman spectroscopy.^[17]^ Unlike neutral Ama, there have been only very few studies of the Ama^+^ radical cation, the target of the present work. Low‐resolution electron momentum spectra show that ionization of Ama into the electronic ground state of Ama^+^ occurs by removal of an electron from the nonbonding lone pair of the NH_2_ group, with a rough estimate of the vertical ionization energy of 8.6 eV.^[16b]^ Early mass spectrometric studies of 1‐substituted derivatives of Ada have suggested opening of the adamantyl cage by breaking the C−C bond adjacent to the substituent, with the major fragmentation channel for Ama^+^ being elimination of C_4_H_9_ leading to the formation protonated aniline.^[18]^

Compared to Ama^+^, considerably more information is available for the Ada^+^ parent cation, which has been characterized by photoelectron, photoionization, and fragmentation spectroscopy, IR and electronic photodissociation (IRPD, EPD) spectroscopy, and quantum chemical calculations.[^3l^, ^5b^, ^19^] IRPD spectra of Ada^+^L cluster ions (L=He, N_2_, H_2_O) indicate Jahn‐Teller distortion and C−H bond activation upon ionization into the cation ground state.[^19g^, ^20^] Significantly, all transitions observed could be assigned to cluster isomers composed of the nascent cage structure of Ada^+^, consistent with low‐resolution EPD spectra of bare Ada^+^ generated by electron ionization (EI).^[5b]^ Due to vertical ionization given by the Franck‐Condon principle, photoelectron and photoionization spectra of Ada only probe the vibronic structure of the nascent cation geometry and thus detect solely the canonical cage structure of Ada^+^.[^3l^, ^19b^, ^19l^, ^19m^, ^19o^] Fragmentation mass spectra of Ada^+^ generated by VUV and XUV photoionization as well as EI indicate substantial fragmentation.[^19e^, ^19f^, ^19k^, ^19n^] Interestingly, quantum chemical analysis of the involved fragmentation processes postulate stable open‐cage isomers of Ada^+^ as local and even global minima on the potential energy surface as reaction intermediates prior to fragmentation, although experimental evidence for such minima has not been presented.[^19e^, ^19f^, ^19n^] In the condensed phase, diamondoid derivatives with open‐cage structures have been prepared by chemical reactions.^[21]^ Such species are of interest, because they are well suited for complexation with transition metals.^[22]^ Studying the opening process of diamondoid cages is also of interest for understanding chemical reactions in which cage opening and reclosing occurs. To this end, it has been shown that a reaction of 1‐hydroxydiamantane with elemental bromine leads to consecutive opening and reclosing of the cage, providing straightforward access to a class of previously unknown 1,2‐disubstituted diamondoid derivatives.^[23]^ However, in none of the condensed‐phase studies, the open‐cage intermediates could be trapped or detected as stable cations.

**Figure 1:**
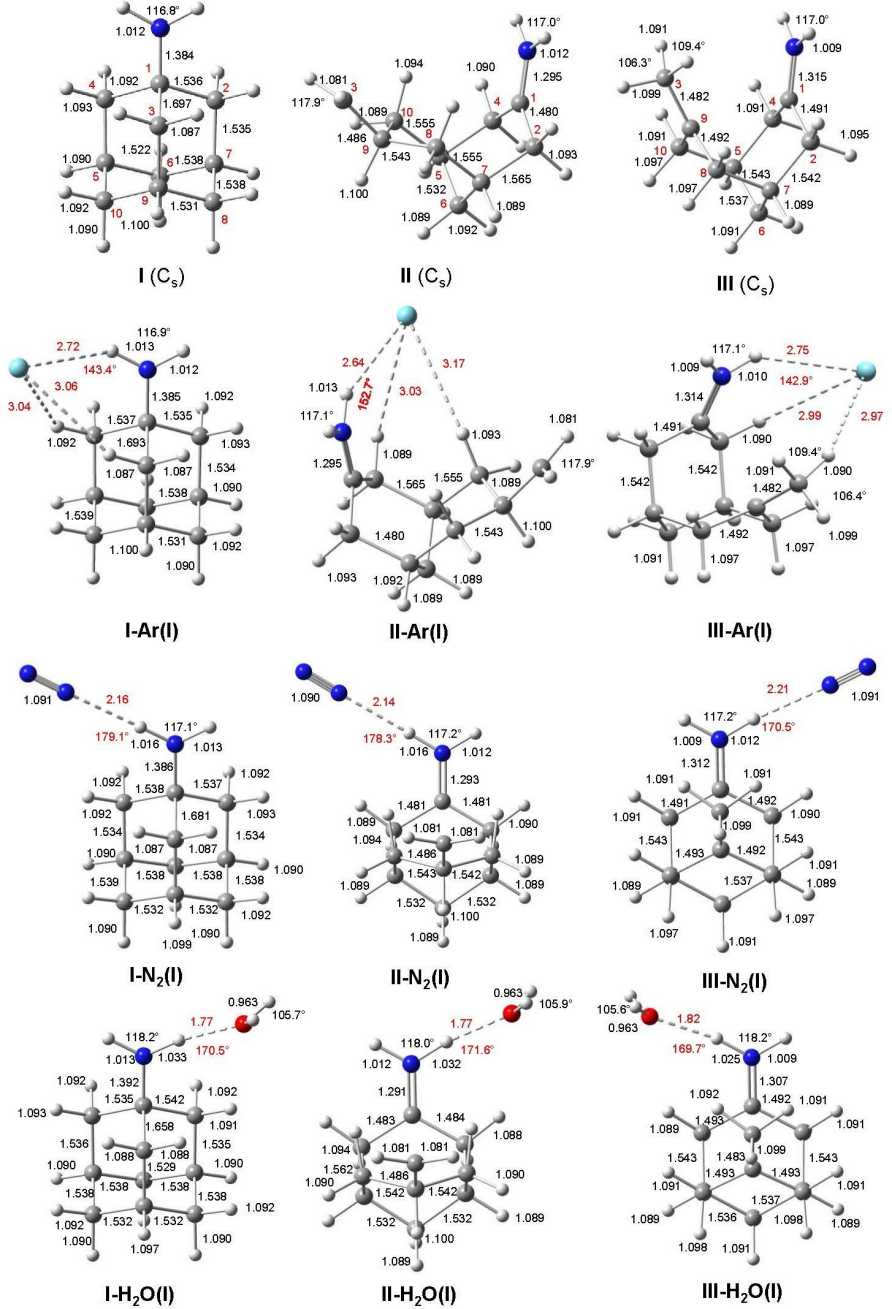
Calculated equilibrium structures (in Å and degrees) of Ama^+^(**I–III**) and **(I–III**)**‐L**(**I**) with L=Ar, N_2_, and H_2_O in their ground electronic state (B3LYP−D3/cc‐pVTZ). The numbering of C atoms is indicated by red numbers. Intermolecular bond lengths and angles are indicated by red values.

**Figure 2:**
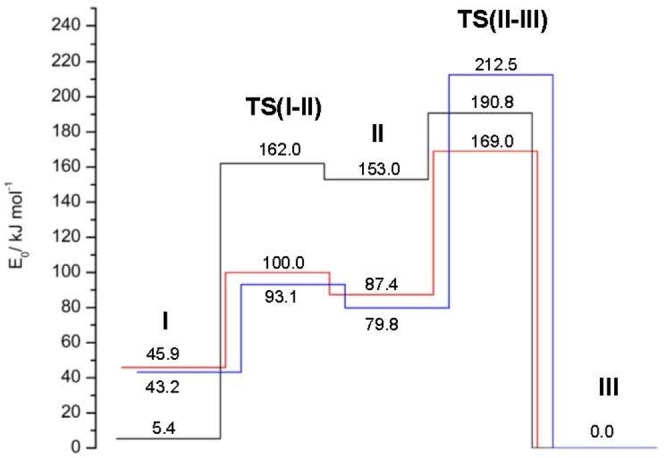
Potential energy surfaces (minima and transition states) of Ama^+^ (red), Ama^+^H_2_O (blue), and Ada^+^ (black) for the cage‐opening reaction upon ionization.

With the major goal of characterizing the ionization‐induced cage‐opening process of diamondoid cations in the gas phase, we analyze herein IRPD spectra of size‐selected Ama^+^L_*n*_ clusters with L=Ar (*n*=1‐3), L=N_2_ (*n*=1), and L=H_2_O (*n*=1) generated in an EI source by means of dispersion‐corrected density functional theory (DFT) calculations at the B3LYP−D3/cc‐pVTZ level. Ama^+^ was chosen for the study of rearrangement processes of the adamantyl cage (C_10_H_15_) upon ionization for the following reasons. Our previous studies on Ada^+^L and Ama^+^(H_2_O)_1‐3_ using the same experimental and computational approach indicate that, as a result of H→NH_2_ substitution, one of the C−C bonds of Ama^+^ is substantially less stable and highly activated compared to Ada^+^.[^19g^, ^20^, ^24^] Thus, Ama^+^ has a strongly reduced barrier to cage opening which may thus be observable. Indeed, ionization of Ama into its cation ground state occurs by removal of an electron from the nonbonding N lone pair rather than from a bonding C−C σ orbital completely delocalized over the cycloalkane cage in Ada^+^. Within the ligand series Ar<N_2_<H_2_O, we tune the type and strength of the interaction of the solvent molecules with Ama^+^. While Ar will only weakly interact with Ama^+^ via dispersion and induction (charge‐induced dipole), the interaction of N_2_ will be somewhat stronger due to additional electrostatic charge‐quadrupole forces, as has previously been shown for related amine cation clusters (such as aniline^+^ and 4‐aminobenzonitrile^+^).^[25]^ On the other hand, H_2_O will form a strong NH…O ionic hydrogen bond (H‐bond) to Ama^+^ based mostly on electrostatic charge‐dipole interaction.[^24^, ^26^] Thus, the weak interaction of Ar and N_2_ with Ama^+^ is expected to have only a negligible influence on the structural isomers and the rearrangement process of the Ama^+^ core ion, while its strong interaction with H_2_O may substantially perturb the potential energy surface of the Ama^+^ cage‐opening reaction. In a recent letter,^[27]^ we have already reported a preliminary analysis of the IRPD spectrum of the Ama^+^Ar dimer (*n*=1), and the main results may be summarized as follows. In addition to the canonical nascent Ama^+^ isomer with an intact C_10_H_15_ cage (**I**, ∼50 %), we could unambiguously identify two distonic bicyclic iminium isomers in which the adamantyl cage opens upon ionization (**II**/**III**, ∼15/35 %), whereby **III** is in fact lower in energy than the canonical cage isomer **I** (Figure 1 and 2). Moreover, we determined the reaction profile with barriers and intermediates along this cage‐opening reaction by DFT calculations and rationalized their relative abundance. In the present work, we provide a full account of this cage‐opening process by presenting and analyzing the IRPD spectra of larger Ama^+^Ar_*n*_ clusters (*n*≤3) and Ama^+^L dimers with L=N_2_ and H_2_O to determine the effect of solvent molecules with different binding energy on the reaction profile. In fact, we have studied previously the microhydration process of Ama^+^ by IRPD and DFT of Ama^+^(H_2_O)_*n*_ clusters up to *n*≤3.^[24]^ While the results gave a first impression of the structure and IR spectrum of bare Ama^+^ and the microhydration structure of the cage isomer (**I**), no evidence of the open‐cage bicyclic isomers was found.^[24]^ To this end, we report herein a new IRPD spectrum of Ama^+^H_2_O with an improved signal‐to‐noise ratio, which allows us to identify also the open‐cage structures of Ama^+^H_2_O (**II/III**). We also briefly discuss why the cage‐opening process is suppressed for Ada^+^ and its clusters based on the effect of H→NH_2_ substitution on the potential energy profile.

## Experimental and Computational Techniques

IRPD spectra of Ama^+^L_*n*_ clusters are obtained in a quadrupole tandem mass spectrometer coupled to an EI cluster source and an octupole ion trap.^[28]^ Cold Ama^+^L_*n*_ clusters are generated in a pulsed supersonic plasma expansion by electron and/or chemical ionization of a heated Ama sample (Sigma Aldrich, >97 %, *T*∼130°) seeded in a suitable carrier gas (Ar or N_2_, 10–20 bar stagnation pressure) close to the nozzle orifice and three‐body clustering reactions. For the generation of Ama^+^H_2_O clusters, a small amount of distilled water is added to the gas inlet system. The filaments of the EI source are operated at an offset voltage of up to 200 V, which provides the upper limit for the kinetic energy of the electrons hitting the molecular beam. These electrons are (in)elastically scattered by collisions with atoms/molecules in the high‐pressure region of the expansion and produce secondary electrons by ionization events. Ama^+^ ions are then generated either by EI with primary or secondary electrons or by chemical ionization (e. g., Penning ionization or charge transfer with excited or ionized carrier gas). As a result of these complex processes in the plasma expansion, the initial excess ionization energy deposited in Ama^+^ upon ionization is not well characterized. The generated Ama^+^ and resulting fragment ions are cooled by collisions and form clusters by three‐body aggregation. The upper limit of the internal energy (and thus the effective temperature) of the generated Ama^+^L_*n*_ clusters is then given by the dissociation energy of the ligands, which increases in the order Ar<N_2_<H_2_O. After mass selection by the first quadrupole, the desired parent cluster ion is irradiated in the adjacent octupole with a tunable IR laser pulse (*ν*_IR_) of an optical parametric oscillator (10 Hz repetition rate, <4 cm^−1^ bandwidth) pumped by a nanosecond Q‐switched Nd:YAG laser. The IR laser is characterized by a pulse energy of 2–5 mJ in the 2600–3800 cm^−1^ range and 0.1–0.5 mJ in the 1200–1800 cm^−1^ range. The laser frequency is calibrated using a wavemeter. Resonant vibrational excitation of the cluster ions causes the loss of all ligands in the size range investigated. The produced Ama^+^ fragments are selected by the second quadrupole and monitored by a Daly detector as a function of laser frequency to obtain IRPD spectra of Ama^+^L_*n*_. The ion source is triggered at twice the laser frequency, which facilitates subtraction of background signal arising from metastable decay. All IRPD spectra are normalized for frequency‐dependent variations in the photon flux. Collision‐induced dissociation experiments (CID) of Ama^+^L_*n*_ clusters mass‐selected by the first quadrupole are performed to confirm their chemical composition (Figure S1). To this end, the octupole is filled with N_2_ gas (10^−5^ mbar) leading to collisions with the mass‐selected ions at a collision energy of 10 eV in the laboratory frame.

Geometries, energies, and harmonic IR spectra of stable Ama^+^ isomers and their corresponding clusters are calculated using dispersion‐corrected DFT calculations at the unrestricted B3LYP−D3/cc‐pVTZ level.^[29]^ This computational method has already provided reliable results for Ama^+^(H_2_O)_*n*_ and Ada^+^H_2_O, and is an efficient compromise between computing time and accuracy.[^20^, ^24^] Relative energies and binding energies (*E*_e_, *D*_e_) are corrected for harmonic zero‐point vibrational energy to yield *E*_0_ and *D*_0_. Free energies (*G*) are obtained at room temperature (*T*=298.15 K). We will discuss herein only the energies at zero Kelvin, because entropy has only little impact on the reaction profile, but provide *G* values in the Supporting Information for comparison. Harmonic frequencies of NH/OH stretch modes (*ν*_NH/OH_) are scaled by a factor of 0.9491 derived from fitting *ν*_NH_ stretch frequencies of neutral Ama to experimental values.^[16c]^ Since the adamantyl cage of Ama and Ada are similar, the CH stretch frequencies (*ν*_CH*n*_) are scaled by a factor of 0.9618 derived from the experimental data for Ada^+^He_2_.^[19g]^ The CH_2_/NH_2_ bending modes and other vibrations occurring in the fingerprint range below 2000 cm^−1^ are scaled by 0.9732 derived from fitting the NH_2_ bending frequency of the related aniline cation to the experimental value of the NH_2_ bending overtone.^[25b]^ The use of three scaling factors accounts for the somewhat different anharmonicities of the various stretching and bending modes.^[27]^ Computed IR stick spectra are convoluted with Gaussian line profiles (fwhm=10 cm^−1^). In a few cases, anharmonic vibrational calculations are performed to estimate frequencies and IR intensities of overtones and combination bands.^[29]^ Natural bond orbital (NBO) analysis is used to evaluate the charge distribution, charge transfer, and spin density.^[30]^ A complete set of all relevant structures (Figure S2–S5), NBO charge and spin data (Figure S6–S13), computed and experimental IR spectra (Figure S14–S33, Tables S1–S6), potential energy surfaces (Figure S35), and HOMO orbitals (Figure S35–S36) are given in the Supporting Information, along with Cartesian coordinates and energies (Tables S7–S13), while the most important information is presented in Figure 1 and 8 and Table 1.

**Table 1:** Positions, widths (fwhm in parenthesis) and vibrational and isomer assignments of the transitions observed in the IRPD spectra of Ama^+^Ar_1‐3_, Ama^+^N_2_, and Ama^+^H_2_O (Figure 3 and 4).^[a]^

Peak	Mode^[b]^	Ama^+^Ar	Ama^+^Ar_2_	Ama^+^Ar_3_	Ama^+^N_2_	Ama^+^H_2_O	Isomer
**R**	*ρ*_NH2_, *τ*_CH2_, *γ*_CH2_	1228 (15)					**I, III**
**S**	*ρ*_NH2_, *γ*_CH2_, *γ*_CH3_	1368 (35)					**II, III**
**P**	*β*_CH2_, *β*_CH3_	1458 (25)					**I–III**
**T**	*ν*_CN_	1551 (7)					**III**
**Q_1_**	*β*_NH2_	1599 (6)					**I**
**Q_2_**	*β*_NH2_	1664 (13)					**III**
**Q_3_**	*β*_NH2_	1717 (7)					**II**
**A**	*ν*_CH_	2842 (4)	2844 (8)	2851 (4)	2841 (7)		**II**
**B**	*ν*_CH_, *ν*_CH2_, *ν*_CH3_	2864 (11)	2870 (15)	2873 (15)	2876 (16)	2865 (30)	**I, III**
**C**	*ν*_CH2_, *ν*_CH3_	2920 (6)	2921 (11)	2924 (8)	2923 (11)		**I–III**
**D**	*ν*_CH_, *ν*_CH2_	2947 (25)	2944 (10)	2946 (8)	2944 (11)	2937 (45)	**I–III**
**E**	*ν*_CH_, *ν*_CH2,_ *ν*_CH3_	2959 (6)	2963 (9)	2972 (6)	2966 (7)		**I–III**
**F**	*ν*_CH2_	2978 (4)	2984 (6)	2986 (5)	2984 (5)		**I–III**
**G**	*ν*_CH2_	3008 (6)			3020 (4)		**I, II**
**U**	*ν*_CH2_				3103 (5)		**II**
**H**	2*β*_NH2_	3151 (9)	3152 (12)	3152 (13)	3172 (14)	3187 (15)	**I**
**V**	2*β*_OH2_					3213 (13)	**I–III**
**I**	2*β*_NH2_	3267 (8)	3270 (7)	3273 (8)	3282 (9)	3318 (16)	**III**
**L_1_**	*ν*_NH_^b^					2990 (30)	**I**
**L_2_**	*ν*_NH_^b^					3021 (45)	**II**
**L_3_**	*ν*_NH_^b^					3147 (26)	**III**
**J_1_**	*ν*_NH_^s^	3321 (16)	3316 (17)	3319 (11)	3307 (15)		**I**
**J_2_**	*ν*_NH_^s^	3345 (8)	3351 (5)	3355 (9)	3316 (3)		**II**
**J_3_**	*ν*_NH_^s^	3381 (17)	3379 (16)	3384 (13)	3372 (18)		**III**
**K_1_**	*ν*_NH_^a^	3425 (9)	3419 (10)	3422 (8)	3417 (12)		**I**
**K_2_**	*ν*_NH_^a^	3451 (7)	3439 (9)	3444 (8)	3427 (2)		**II**
**K_3_**	*ν*_NH_^a^	3477 (11)	3474 (13)	3477 (10)	3467 (13)		**III**
**M_1_**	*ν*_NH_^f^					3368 (27)	**I**
**M_2_**	*ν*_NH_^f^					3387 (10)	**II**
**M_3_**	*ν*_NH_^f^					3433 (12)	**III**
**N**	*ν*_OH_^s^					3627 (31)	**I–III**
**O**	*ν*_OH_^a^					3717 (28)	**I–III**

[a] All values are given in cm^−1^. [b] Stretching (*ν*), bending (*β*), torsion (*τ*), wagging (*γ*), rocking (*ρ*).

## Experimental Results

Typical mass spectra of the EI source are shown in Figure S1. The major observed fragments of the parent Ama^+^ ion (*m/z* 151) occur at *m/z* 136, 108, 94, and 57. These are consistent with the standard EI mass spectrum of Ama and confirm the parent ion as Ama^+^, with protonated aniline (*m/z* 94) being the most intense fragment ion.[^16c^, ^18^] In addition, different clusters of Ama^+^ and its fragments with neutral ligands (L=Ar, N_2_, H_2_O, Ama) are generated in the supersonic plasma expansion. CID spectra of size‐selected Ama^+^L_*n*_ cluster ions show merely the loss of neutral ligands, confirming the composition of the clusters and ruling out any isobaric contamination (Figure S1).

**Figure 3:**
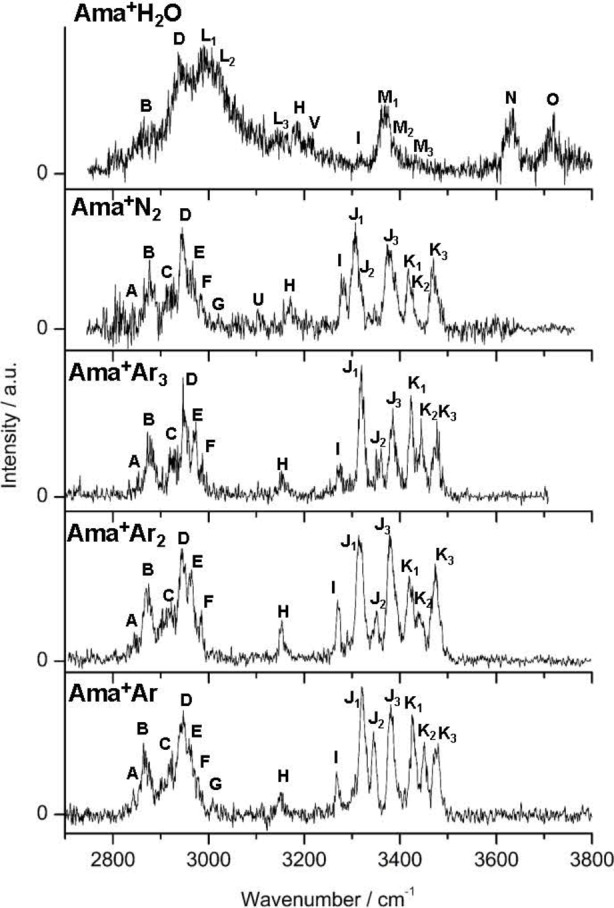
IRPD spectra of Ama^+^L_*n*_ (L=Ar, N_2_, H_2_O) in the XH stretch range recorded in the Ama^+^ channel. The positions and widths of the transitions observed (**A‐K**) are listed in Table 1, along with their vibrational and isomer assignments.

**Figure 4:**
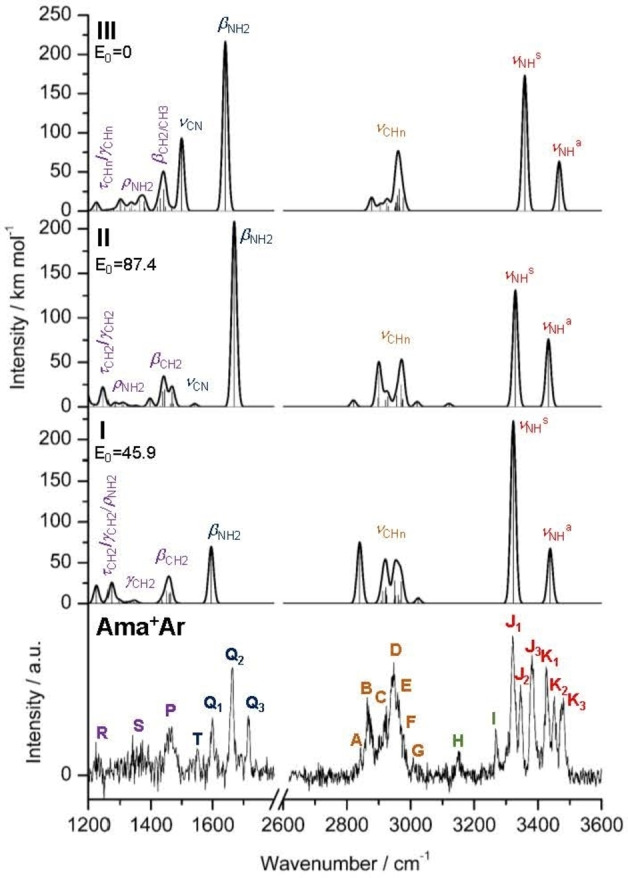
IRPD spectra of Ama^+^Ar in the XH stretch and fingerprint ranges compared to linear IR absorption spectra of Ama^+^(**I–III**) calculated at the B3LYP−D3/cc‐pVTZ level (reproduced with permission from Ref. [27], copyright 2022 American Chemical Society). The positions of the transitions observed and their vibrational and isomer assignment are listed in Table S2. Relative energies (*E*_0_) are given in kJ mol^‐1^.

The IRPD spectra of all investigated Ama^+^L_*n*_ clusters recorded in the XH stretch range (2700–3800 cm^−1^) are compared in Figure 3, while the IRPD spectrum of Ama^+^Ar obtained in the fingerprint range (1200–1800 cm^−1^) is shown in Figure 4. The positions and widths of the transitions observed are listed in Table 1, along with the suggested vibrational and isomer assignments derived from the DFT calculations. The IRPD spectrum of Ama^+^Ar was reported and partially analyzed before,^[27]^ while the Ama^+^H_2_O spectrum in Figure 3 provides an improved spectrum over the one reported earlier, with more discernible features not identified previously (**L_2_**, **L_3_**, **V**, **M_2_**, **M_3_**).^[24]^ The probed XH stretch range (2700‐3800 cm^−1^) covers aliphatic CH_*n*_ stretch modes (*ν*_CH/CH2/CH3_) of the C_10_H_15_ moiety (**A‐G**, **U**), symmetric (*ν*_NH_^s^) and antisymmetric NH stretch (*ν*_NH_^a^) modes of the NH_2_ group of Ama^+^ (**J_1‐3_**, **K_1‐3_**), and overtone bands of the NH bend (*β*_NH2_) and OH bend (*β*_OH2_) fundamentals (**H**, **I**, **V**). In the Ama^+^H_2_O spectrum, we also identify bound (*ν*_NH_^b^) and free NH stretch (*ν*_NH_^f^) modes (**L**, **M**), as well as symmetric and antisymmetric free OH stretch fundamentals of the H_2_O ligand (*ν*_OH_^s^, *ν*_OH_^a^) in the higher frequency range (**N**, **O**). Moreover, the fingerprint range (1200–1800 cm^−1^) investigated for Ama^+^Ar covers CH_*n*_ and NH_2_ bending (*β*), rocking (*ρ*), torsion (*τ*) and wagging (*γ*) modes as well as *ν*_CN_ fundamentals (**R**, **S**, **P**, **T**, **Q_1‐3_**). Several observed transitions exhibit systematic shifts as a function of cluster size and ligand type, which provide useful information about the ligand binding site and interaction strength. Clearly, the overall similar appearance of the Ama^+^Ar_*n*_ and Ama^+^N_2_ spectra arise from weak intermolecular interaction of Ama^+^ with Ar and N_2_, while the Ama^+^H_2_O spectrum differs strongly due to the strong NH…O ionic H‐bonds.^[24]^ In the following, we provide a rough mode assignments based on experimental data only.

The NH stretch range of the Ar and N_2_ clusters reveals six transitions within 3300 and 3500 cm^−1^, which are readily assigned to *ν*_NH_^s^ (**J_1‐3_**) and *ν*_NH_^a^ (**K_1‐3_**) fundamentals of the NH_2_ group of three Ama^+^ isomers (**I–III**) with rather different N−H bond properties.^[27]^ Clearly, the NH stretch modes show the largest spectral shifts, indicating that the ligands preferentially bind to the positively charged NH_2_ group of **I–III**. The shifts between the *n*=1 and *n*=3 spectra of Ar (≤12 cm^−1^) are somewhat smaller than those between Ar and N_2_ for *n*=1 (≤29 cm^−1^). Such small shifts are typical for weak van‐der‐Waals or H‐bonds, which do not decouple the two NH stretch normal modes of the NH_2_ group, justifying their notation as *ν*_NH_^s^ and *ν*_NH_^a^. In contrast, the strong NH…O ionic H‐bonds of all three Ama^+^H_2_O isomers decouple the NH stretch normal modes, leading to large red‐shifts for the bound local NH stretch modes (*ν*_NH_^b^, **L_1‐3_**), while the free NH stretch modes (*ν*_NH_^f^, **M_1‐3_**) occur between *ν*_NH_^s^ and *ν*_NH_^a^ of the bare Ama^+^ isomers.^[24]^ Significantly, while the intense *ν*_NH_^b/f^ bands of the cage isomer (**I**) were identified previously (**L_1_**, **M_1_**), the corresponding weaker bands assigned to the other two bicyclic isomers (**II**, **III**) are identified herein for the first time (**L_2/3_**, **M_2/3_**) due to the higher signal‐to‐noise ratio achieved.

The **N** and **O** bands at 3627 and 3717 cm^−1^ are solely observed for Ama^+^H_2_O and readily assigned to free *ν*_OH_^s/a^ modes of the H_2_O ligand.^[24]^ Their red‐shifts of 30 and 39 cm^−1^ from the corresponding transitions of bare H_2_O (3657 and 3756 cm^−1^) are typical for cation‐H_2_O complexes with charge‐dipole configuration, in which the O atom of H_2_O is pointing toward the positive charge.[^24^, ^26^] The relatively large width of these two transitions suggests that the *ν*_OH_^s/a^ modes of the assigned isomers **I–III** are not resolved.

Except for the Ama^+^H_2_O spectrum, the IRPD spectra of all Ama^+^L_*n*_ clusters have very similar appearance in the CH stretch range, with respect to both frequencies (to within 13 cm^−1^) and relative IR intensities for the *ν*_CH*n*_ stretch fundamentals of Ama^+^ (**A**‐**F**). The spectra are better resolved for Ama^+^Ar_2/3_ because larger clusters are colder. For Ama^+^H_2_O, the CH stretch range is strongly affected by the red‐shifted and broadened *ν*_NH_^b^ transition of the NH…O ionic H‐bond, which carries high IR oscillator strength and thus is involved in NH/CH stretch local mode mixing and anharmonic interactions (e. g., Fermi resonances with CH and CC overtones and combination bands).[^24^, ^26c^, ^26d^, ^26h^, ^31^]

Apart from the transitions readily attributed to NH, OH, and CH stretch fundamentals, the IRPD spectra display in the XH stretch range transitions with less obvious assignments. For example, band **H** near 3150 cm^−1^ for Ar shifts gradually to the blue for ligands with stronger interaction (3172 and 3187 cm^−1^ for N_2_ and H_2_O), and the same trend is observed for peak **I** (3267, 3282, 3318 cm^−1^ for Ar, N_2_, H_2_O), indicating that these bands are combination or overtone transitions including the bending, scissoring, or wagging motions of the NH_2_ group. H‐bonding with L generates an additional retarding force for these NH_2_ modes and thus to higher frequencies for stronger NH…L bonds. The weak and tentative band **U** (∼3103 cm^−1^) is only observed in the Ama^+^N_2_ spectrum, while the very weak feature **G** (∼3010 cm^−1^) is only resolved in the Ama^+^Ar and Ama^+^N_2_ spectra. Moreover, an additional peak **V** is observed only for H_2_O at 3213 cm^−1^, suggesting an assignment to a combination or overtone of the H_2_O bend (*β*_OH2_).

The fingerprint range is considered only for Ama^+^Ar, with the main purpose of confirming the number of Ama^+^ isomers inferred from the NH stretch range.^[27]^ Indeed, the fingerprint spectrum of Ama^+^Ar is dominated by three intense, narrow, and isolated peaks in the NH_2_ bending range (1599, 1664, 1717 cm^−1^) assigned to *β*_NH2_ (scissoring mode) of **I–III** (**Q1‐Q3**). There are four other, weaker, and broader peaks **R**, **S, P**, and **T** observed in the range of the *β*_CH2_, *γ*_CH2_, *τ*_CH2_, *ρ*_NH2_, and *ν*_CN_ modes, whose detailed assignment has to rely on the DFT calculations. As these bands turn out not to be isomer‐specific (apart from **T**), we do not consider them in detail further. Significantly, the fingerprint spectrum of Ama^+^Ar suggests **H** and **I** to be overtones of **Q1** and **Q2**, while the corresponding overtone of **Q3** is probably hidden in the dense NH stretch range.

## Computational Results and Discussion

The very similar IRPD spectra of Ama^+^Ar_*n*_ suggest only a minor impact of the Ar ligands on the properties of the Ama^+^ core ion. To this end, the Ama^+^Ar spectrum has previously been used to identify the three relevant Ama^+^ isomers **I–III**,^[27]^ mostly by the different properties of their NH_2_ group resulting in rather different *ν*_NH_ and *β*_NH2_ frequencies. Hence, we briefly summarize the computational results for **I–III** and compare them to the experimental Ama^+^Ar spectrum. Subsequently, we will discuss the effects of the ligands on the structural, vibrational, and energetic properties of **I–III** and finally evaluate the effect of H→NH_2_ substitution.

## Ama^+^ isomers I–III

A potential energy diagram for the ionization‐induced rearrangement reaction of Ama is shown in Figure 2. Vertical ionization of neutral Ama (^1^A’, *C*_s_) leads to the nascent cage isomer **I** of the Ama^+^ radical cation (^2^A’, *C*_s_, *E*_0_=45.9 kJ mol^−1^). This ionization process activates one of the C−C bonds, which readily breaks and then leads through cage opening to the formation of the bicyclic distonic isomer **II** (^2^A’, *C*_s_, *E*_0_=87.4 kJ mol^−1^) via transition state **TS(I–II)** located at a barrier of *V*_b_=54.1 kJ mol^−1^ for the forward reaction. A subsequent 1,2 H‐shift then forms via **TS(II–III)** at *V*_b_=81.6 kJ mol^−1^ the most stable isomer **III** (^2^A’, *C*_s_, *E*_0_=0 kJ mol^−1^), which is also a distonic ion and 45.9 kJ mol^−1^ more stable than the nascent cage isomer **I**. The structures along with the atomic numbering of neutral Ama and the three Ama^+^ isomer **I–III** are compared in Figure 1 (and S2), while partial charges and electron spin densities are listed in Figure S6 and S7. The computed IR spectra are plotted in Figure 4 (and S14), while relevant vibrational frequencies are listed in Tables S1 and S2.

In neutral Ama, the NH_2_ group is attached in a pyramidal configuration (sp^3^ hybridization of N) to the C_10_H_15_ cage via a C−N single bond (*r*_CN_=1.467 Å). H→NH_2_ substitution has little impact on the geometry of the C_10_H_15_ cage, with a minor but noticeable activation of the C1−C3 bond adjacent to the NH_2_ group in the *C*_s_ symmetry plane (1.544 vs. 1.538 Å, see Figure S4 for the structure of Ada). In general, the calculated structure and IR spectrum of Ama agree well with previous computational and experimental data.[^16b^, ^16c^, ^16d^, ^16e^] Ionization of Ama into its ground electronic state by removal of an electron from the N lone pair orbital of the NH_2_ group (HOMO, Figure S35) results in the formation of isomer **I** of Ama^+^. Due to orbital conjugation and charge delocalization, the partial charge on the NH_2_ group increases by 0.474 *e*, the C−N bond order increases from 1 to ∼1.5 (*r*_CN_=1.384 Å), the pyramidal NH_2_ group becomes essentially planar (similar to ionization of NH_3_), and the NH_2_ angle increases from 106° to 117° (sp^2^ hybridization of N). The N−H bonds contract by 3 mÅ, resulting in substantial blue‐shifts in the NH stretch frequencies upon ionization (Δ*ν*_NH_^s/a^∼40/80 cm^−1^, Figure S14). As a result of the higher positive partial charges on the NH protons (*q*_H_=0.405 *e*), the IR intensities of the *ν*_NH_ fundamentals are enhanced by 2–3 orders of magnitude. This effect is crucial because detection of the *ν*_NH_ bands of Ama^+^L_*n*_ is essential for isomer identification. Significantly, ionization of Ama has also a strong impact on the C−C bonds lying in the *C*_s_ symmetry plane due to conjugation with the NH_2_ lone pair. In particular, the C1−C3 bond adjacent to the NH_2_ group, already slightly activated by 6 mÅ in neutral Ama by H→NH_2_ substitution and eventually breaking when the cage opens to form isomer **II**, is further strongly destabilized and elongated by 153 mÅ upon ionization. The adjacent C3−C9 bond in the *C*_s_ plane shortens by merely 16 mÅ due to the increased distance from the N lone pair, while all other C−C bonds of the cage remain almost unchanged. Ionization increases the spread in the C−H bond lengths (Δ*r*_CH_=8 vs. 3 mÅ), resulting in a more pronounced splitting of the intense convoluted and mostly unresolved doublet band of the CH_*n*_ stretch modes of Ama (2890 and 2917 cm^−1^) into four better resolved single bands at 2840, 2920, 2955, and 3024 cm^−1^. The NH_2_ bending mode (*β*_NH2_) is slightly red‐shifted by 14 cm^−1^ to 1595 cm^−1^, and its IR intensity increases by a factor of 2. As a result, it becomes the most intense fundamental in the fingerprint range, and this aspect is again crucial because, in addition to *ν*_NH_, also the *β*_NH2_ mode provides a sensitive probe of the isomeric structure of Ama^+^. Due to substantial charge delocalization from NH_2_ to C_10_H_15_, the adamantyl cage carries most of the excess positive charge (*q*_C10H15_=0.687 *e*, *q*_NH2_=0.313 *e*), whereby the acidic NH protons (*q*_H_=0.405 *e*) still serve as attractive proton donors for H‐bonds with neutral ligands. Finally, the adiabatic ionization energy of IE=7.95 eV predicted for **I** is consistent with the rough estimation for the vertical value of 8.6 eV obtained from a low‐resolution electron momentum spectrum.^[16b]^

The bicyclic open‐cage isomer **II** is formed from **I** by breaking the strongly activated C1−C3 bond. At the same time, the cyclohexane ring is converted from the chair into the boat configuration. As a result, the C1…C3 distance of the broken bond increases to 4.650 Å, while the *θ*_C1C9C3_ angle in the *C*_s_ plane opens from 39.2° to 125.1°. This structural rearrangement forms a CH_2_⋅ radical site and a C=N double bond (*r*_CN_=1.295 Å), and is accompanied by a separation of charge and spin. The spin density of this distonic iminium ion is located on the CH_2_⋅ radical site (*s*_C3_=1.005), while the positive charge is mostly localized at the CNH_2_ charge site (*q*_CNH2_=0.754 *e*). While the C−C bonds to the CH_2_⋅ and CNH_2_ groups strongly contract (Δ*r*_C3C9_=−36, Δ*r*_C1C2_=−56, Δ*r*_C6C7_=−6 mÅ), the other C−C bonds are elongated upon the chair→boat transformation (Δ*r*_C2C7_=30, Δ*r*_C7C8_=17, Δ*r*_C8C9_=12 mÅ). The largest changes in the C−H bond lengths occur on the CH_2_⋅ group (+6 mÅ), while all others change less than 3 mÅ. As a result of cage opening, the *ν*_CH*n*_ modes of **II** are more spread than for **I**, with three convoluted bands at 2901, 2926, and 2972 cm^−1^ and weaker high‐frequency *ν*_CH2_^s/a^ modes of CH_2_⋅ at 3021 and 3121 cm^−1^. The *ν*_C9H_ mode is red‐shifted down to very low frequency (2821 cm^−1^) and has lost almost all IR activity. Due to the increase in C−N bond order, the CNH_2_^+^ group in **II** is even more planar than in **I**, and as a result *β*_NH2_ couples strongly with the blue‐shifted *ν*_CN_ mode, leading to a very intense and blue‐shifted *β*_NH2_ band at 1670 cm^−1^ and a weak *ν*_CN_ band at lower frequency (1542 cm^−1^). In line with the minor changes in the N−H bond lengths (≤1 mÅ), *ν*_NH_^s/a^ exhibit small shifts of +7/−5 cm^−1^. The slightly larger positive charge on the NH_2_ protons (*q_H_* =0.421 *e*) makes this binding site for neutral ligands even more attractive than in **I**.

The bicyclic open‐cage isomer **III** is formed from **II** by a 1,2 H‐shift from C9H to the terminal CH_2_⋅ group. As a result, the primary radical **II** (CH_2_⋅) is transformed into a more stable tertiary radical **III** (CR_3_⋅), which thus becomes by far the global minimum on the Ama^+^ potential (Figure 2). This CR_3_⋅ radical group becomes nearly planar (sp^2^ hybridization of C9) with bond angles of 121.2° and 115.8°, while the terminal CH_3_ group becomes pyramidal (sp^3^ hybridization of C3) with bond angles of 106.3° and 109.5°. Parallel to the H‐shift, the cyclohexane ring changes back from boat to chair. As a result, the C1…C3 distance contracts again to 3.346 Å and *θ*_C1C9C3_ decreases slightly to 106.1°. Interestingly, there is substantial orbital overlap between the 2*p*_z_ orbital of the C9R_3_⋅ radical center and the 2*pπ* orbital of the C=N double bond (as indicated by the HOMO, Figure S35), consistent with the short C1…C9 separation of 2.616 Å and the slightly pyramidal structure of the C9R_3_⋅ group. The C−C bonds of the CR_3_⋅ group and the C9−C3 bond to the new CH_3_ group contract by 51 and 4 mÅ, respectively. Upon boat→chair back transformation, the C−C bonds adjacent to the NH_2_ group as well as their corresponding parallel C−C bonds are stretched again (Δ*r*_C1C2_=11, Δ*r*_C6C7_=5 mÅ), while the other C−C bonds contract again (Δ*r*_C2C7_=−23, Δ*r*_C7C8_=−12 mÅ). The C−H bonds of the former CH_2_⋅ radical group are stretched again to a value typical for CH_2_ bonds (1.091 Å), and most other C−H bonds are also slightly elongated (Δ*r*_CH_=2 mÅ). As a result, the *ν*_CH_ and *ν*_CH2_ fundamentals move again closer together and form two weak bands at 2878 and 2926 cm^−1^ and one intense band at 2961 cm^−1^. Of course, the low‐frequency acidic *ν*_CH_ mode of **I/II** at 2821/2840 cm^−1^ disappears. Compared to **II**, the C=N double bond of **III** is slightly elongated to 1.315 Å, while the N−H bonds contract from 1.012 to 1.009 Å. As a result of the shortest N−H bonds of all three Ama^+^ isomers, the NH_2_ stretch modes have the highest frequencies (*ν*_NH_^s/a^=3358/3467 cm^−1^). Similar to **II**, *β*_NH2_ of **III** couples to *ν*_CN_, resulting in *β*_NH2_ at 1641 cm^−1^ and *ν*_CN_ at 1500 cm^−1^. Both modes gain, however, in IR intensity, and this effect is more pronounced for the *ν*_CN_ band (factor ∼20). The *β*_CH2_/*β*_CH3_ band at 1440 cm^−1^ also gains in intensity due to the newly formed CH_3_ group in **III**. Similar to **II**, **III** is also a distonic iminium ion, with spin density located on the CR_3_⋅ radical site (*s*_C9_=0.627) and positive charge localized at the CNH_2_ site (*q*_CNH2_=0.608 *e*). The similarly high positive partial charge on the NH_2_ protons (*q*_H_=0.411 *e*) makes this binding site of **III** also very attractive for neutral ligands.

Comparison of the experimental Ama^+^Ar spectrum to the IR spectra computed for the three monomer isomers **I–III** in Figure 4 demonstrates the presence of all three Ama^+^ isomers, in particular the observation of six *ν*_NH_ (**J_1‐3_**, **K_1‐3_**) and three *β*_NH2_ bands (**Q_1‐3_**). A detailed vibrational and isomer assignment is listed in Table S2. Based on relative intensities and frequencies, the three pairs **J_1_/K_1_**_,_ **J_2_/K_2_**, and **J_3_/K_3_** are readily assigned to *ν*_NH_^s/a^ of **I–III**, with a maximum, mean, and median deviation of 23, 14, and 16 cm^−1^ between measured and computed frequencies, respectively. These differences are consistent with the widths of the bands (12±4 cm^−1^) and possible effects of Ar‐tagging (computed to be ≤10 cm^−1^). Concerning the *β*_NH2_ bands (**Q_1‐3_**), a very good match is obtained for isomer **I** between experiment and prediction (Δ*β*_NH2_=4 cm^−1^ for **Q_1_**). The agreement for the distonic isomers **II** and **III** for the corresponding *β*_NH2_ modes is somewhat worse (23 and 47 cm^−1^ for **Q_3_** and **Q_2_**), probably due to the strong coupling between *β*_NH2_ and *ν*_CN_, which may not be accurately described by a single scaling factor for both types of modes (different anharmonicity and bond order for *ν*_CN_). Nonetheless, the direction and magnitude of these *β*_NH2_ band shifts upon this coupling are reproduced reasonably well. Actually, band **T** at 1551 cm^−1^ may be assigned to the intense *ν*_CN_ mode of **III** predicted at 1500 cm^−1^.

By considering the achieved signal‐to‐noise ratio and the calculated and observed IR intensities (peak area) of peaks **Q_1‐3_**, the relative population of the isomers **I**, **II**, and **III** can be roughly estimated as 50, 15, and 35 % (or 10 : 3 : 7), respectively. For this estimate, we assume similar Ar complexation efficiencies for **I–III**, which is justified by their similar Ar binding energies. Such a population ratio is also consistent with the relative intensities in other spectral ranges, although these are more congested and thus less suitable for a reliable quantitative analysis.

In contrast to the range of the rather isolated *ν*_NH_ and *β*_NH2_ modes, where a clear‐cut assignment to the individual isomers **I–III** is feasible due to their rather different NH_2_ properties, the isomer assignment in the other spectral ranges is less obvious due to similar vibrational frequencies and increased spectral congestion. Nonetheless, the observed IRPD spectrum is consistent with the presence of all three Ama^+^ isomers. For example, in the CH stretch range, bands **B**–**D** (2864, 2920, 2947 cm^−1^) can be attributed to the single and isolated *ν*_CH_ mode and overlapping *ν*_CH2_ modes predicted at 2840, 2920, and 2955 cm^−1^ for the predominant **I** isomer, respectively. Similarly, **E** (2959 cm^−1^) and **F** (2978 cm^−1^) can be interpreted as *ν*_CH2_ modes (2961 and 2971 cm^−1^) of the convoluted peak at 2955 cm^−1^, while band **G** (3008 cm^−1^) is assigned to the isolated single *ν*_CH2_ mode (3024 cm^−1^) of the special CH_2_ group adjacent to the elongated C1−C3 bond of **I**. Thus, while most CH stretch bands may readily be explained by the most abundant isomer **I**, there are several indications for the presence of **II** and **III** in this spectral range as well. For example, the weak peak **A** at 2842 cm^−1^ provides strong evidence for isomer **II**, because for **I** and **III** no such low‐frequency CH stretch band is predicted. In addition, the high relative intensity of band **D** is not reproduced by the simulation for **I** and is thus indicative for a significant population of **III**. While the appearance of bands **R**, **S**, and **P** in the Ama^+^Ar spectrum is consistent with the predictions of all three isomers, the well isolated transitions **H** and **I** at 3151 and 3267 cm^−1^ are not reproduced by the calculated spectra of any of the isomers. Hence, they are attributed to overtones and combination bands of modes occurring in the fingerprint range, which are not included in the (scaled) harmonic calculations. Indeed, strong overtone transitions of the scissoring mode (2*β*_NH2_) have been detected in the IR spectra of a variety of amine radical cations, such as (substituted) anilines.[^25^, ^32^] Following this likely scenario, bands **H** and **I** at 3151 and 3267 cm^−1^ are attributed to 2*β*_NH2_ of isomers **I** and **III**, with their *β*_NH2_ fundamentals being bands **Q1** and **Q2** (1599 and 1664 cm^−1^), respectively. The corresponding 2*β*_NH2_ overtone of isomer **II** is estimated near 3400 cm^−1^ from the fundamental band at 1717 cm^−1^ (**Q3**) and thus hidden by the dense and intense NH stretch bands. Anharmonic calculations actually predict significant intensities of such 2*β*_NH2_ bands for **I–III** and thus support this assignment (Tables S2‐S3).

## Ama^+^Ar_*n*_ clusters

The assignment of the Ama^+^Ar spectrum by direct comparison to the spectra computed for the **I–III** monomers is justified by the minor impact of successive Ar solvation on the appearance of the Ama^+^Ar_*n*_ spectra. Hence, the IRPD spectra suggest that the weak interaction with Ar causes only a small but noticeable perturbation on both the structure and IR spectra of **I–III**. This perturbation is most pronounced for the *ν*_NH_ frequencies, which indicates that the NH_2_ group with its acidic and highly charged NH protons are the most attractive binding sites for Ar (and also other neutral ligands). To this end, we have calculated a large variety of Ama^+^Ar_*n*_ isomers for each core isomer (7–13), and their structures, binding energies, IR spectra, and charge distributions are given in Supporting Information. For all considered **I‐Ar**, **II‐Ar**, and **III‐Ar** isomers, the effect of Ar complexation is less than 9 (**I**), 5 (**II**), and 3 cm^−1^ (**III**) for the most sensitive *ν*_NH_ and *β*_NH2_ modes. Similar small effects are predicted for the clusters with two and three Ar ligands. The Ar ligands are bound via weak dispersion and induction forces to the acidic NH_2_ group and/or the C_10_H_15_ moiety with total binding energies of *D*_0_=4.1–8.9 kJ mol^−1^ (*n*=1), 11.2–18.2 kJ mol^−1^ (*n*=2), and 19.8–27.4 kJ mol^−1^ (*n*=3). The energy differences between individual Ama^+^Ar_*n*_ isomers are small and less than Δ*E*_0_=6 kJ mol^−1^ from the global minimum, so that it is difficult to exclude any of them. Hence, we will discuss here briefly only the global minima of Ama^+^Ar_*n*_. The only experimental information about the binding energies of Ama^+^Ar_*n*_ comes from the observation that single‐photon IRPD is sufficient to evaporate all three Ar ligands of Ama^+^Ar_3_ in the XH stretch range (≥2800 cm^−1^), providing an upper limit for the average Ar binding energy of the order of 1000 cm^−1^ (or 12 kJ mol^−1^), which is fully consistent with the computed Ar binding energies.

The preferred binding site of the first Ar ligand in the most stable **I‐Ar(I)**, **II‐Ar(I)**, and **III‐Ar(I)** global minima for each isomer **I–III** is in all cases the NH_2_ group (Figure 1). However, due to substantial charge delocalization into the C_10_H_15_ moiety, the NH…Ar H‐bonds are rather weak (*R*_NH…Ar_=2.64–2.75 Å) and substantially supported by additional van‐der‐Waals type contacts to the adjacent CH_2_/CH_3_ groups (*R*_CH…Ar_=2.97–3.17 Å). As a result, the NH…Ar H‐bonds are strongly nonlinear (*ϕ*_NHAr_=142.9–152.7°), with binding energies of *D*_0_=8.4–8.9 kJ mol^−1^. Upon Ar attachment, the N−H proton donor bonds are slightly elongated (by 1 mÅ) with small red‐shifts in the *ν*_NH_ modes (<9 cm^−1^), while the involved C−H bonds slightly contract (up to 1 mÅ) resulting in minor blue‐shifts in the corresponding *ν*_CH2_ modes (<7 cm^−1^). The charge transfer from Ama^+^ to Ar is also small (6–7 m*e*) upon complexation, and most other bonds are nearly unaffected. The **I‐Ar(II)** isomer with a nearly linear NH…Ar bond but no CH…Ar contact is only slightly higher in energy (Δ*E*_0_=0.7 kJ mol^−1^, *D*_0_=7.7 kJ mol^−1^, *R*_NH…Ar_ =2.59 Å, *ϕ*_NHAr_=167.9°, Figure S16), indicating the subtle competition between the weak NH…Ar H‐bonds and the CH…Ar van‐der‐Waals contacts in these clusters. Nonetheless, also for this stronger NH…Ar H‐bond, the perturbation of the NH_2_ group is still minor (Δ*r*_NH_=1 mÅ). The other less stable Ar binding motifs in **I‐Ar(III–VIII)**, **II‐Ar(II–VII)**, and **III‐Ar(II–VII)** are mainly weak van‐der‐Waals contacts to the C_10_H_15_ moiety further away from the NH_2_ group, with binding energies of *D*_0_=4.1–8.0 kJ mol^−1^, which have even less impact on the monomer structures than isomers with Ar binding to the NH_2_ group.

**Figure 5:**
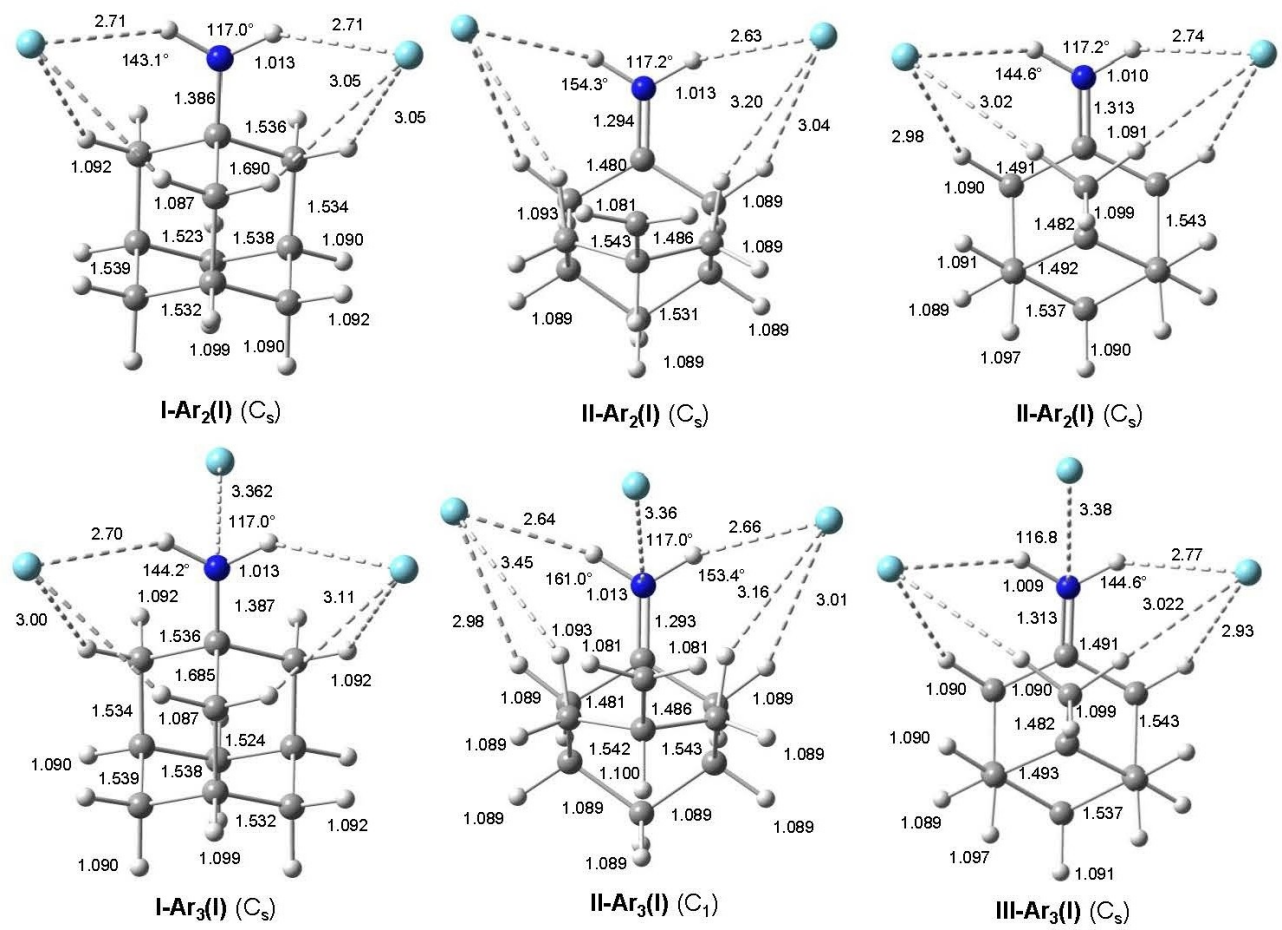
Calculated equilibrium structures (in Å and degrees) of **(I–III)‐Ar_2,3_(I)** in their ground electronic state (B3LYP−D3/cc‐pVTZ).

The preferred binding site of the second Ar ligand in the most stable **I‐Ar_2_(I)**, **II‐Ar_2_(I)**, and **III‐Ar_2_(I)** minima is the mirror image position of the first Ar ligand, thereby restoring *C*_s_ symmetry again (Figure 5). The total binding energies of *D*_0_=16.9–18.2 kJ mol^−1^ are essentially twice that of the corresponding **(I–III)‐Ar(I)** dimers, due the equivalent binding sites offered by the two NH proton donor sites of the NH_2_ group. Apparently, nonadditive three‐body contributions to the intermolecular interactions are very minor. Consequently, the properties of the two nonlinear intermolecular NH…Ar bonds and CH…Ar contacts (*r*_NH…Ar_=2.63–2.74 Å, *ϕ*_NHAr_=143.1°–154.3°, *r*_CH…Ar_=2.98–3.20 Å) are similar to those of the **(I–III)‐Ar(I)** minima with one Ar ligand. The total charge transfer to the ligands (Δ*q*=12‐14 m*e*) increases also linearly with the number of Ar atoms. Overall, the effects on the monomer structures and IR spectra are rather minor also for attaching the second Ar ligand. For all considered Ama^+^Ar_2_ isomers, **I‐Ar_2_(I–VIII)**, **II‐Ar_2_(I–IX)**, and **III‐Ar_2_(I–X)**, the total frequency shifts of the *ν*_NH2_ modes are below 6, 10, and 3 cm^−1^, respectively.

In the most stable **I‐Ar_3_(I)**, **II‐Ar_3_(I)**, and **III‐Ar_3_(I)** isomers, the three ligands are arranged around the NH_2_ group such that the first two Ar atoms form nonlinear NH…Ar H‐bonds with similar binding properties (*R*_NH…Ar_=2.62–2.64 Å, *ϕ*_ArHN_=144.2–161.0°, *R*_CH…Ar_=2.93–3.16 Å) as in **(I–III)‐Ar_2_(I)**, while the third ligand is attached directly to the N atom at *R*_Ar…N_=3.36‐3.38 Å (Figure 5). The N atom also carries substantial positive charge due to electron removal from the N lone pair upon ionization (either a 2*p*_z_ orbital of N in **I** or a 2*pπ* orbital of the C=N group in **II/III**). The total binding energies of *D*_0_=24.7–27.4 kJ mol^−1^ are somewhat less than three times those of **(I–III)‐Ar(I)**, because the N site is less attractive than the two NH sites. As a result of the weaker third intermolecular bond, the total charge transfer to the ligands increases only slightly (Δ*q*=15, 18, 19 m*e*). Also the third Ar ligand has a vanishingly small impact on the monomer structures and their vibrational modes (Δ*ν*_NH2_<6, 10, and 3 cm^−1^ for **I–III**).

As expected, comparison of the IRPD spectra of Ama^+^Ar_1‐3_ with the calculated IR spectra of the various cluster isomers yields the same assignment (Table S3) as the comparison with the spectra computed for the monomers **I–III** because of the weak Ama^+^…Ar interaction. Significantly, the effects of Ar on the *ν*_NH_ modes is much smaller than the splitting observed in the experimental IRPD spectra arising from different isomers, indicating that the bands **J_1‐3_** and **K_1‐3_** do not arise from three distinct Ar binding sites of a single Ama^+^ isomer but from three different Ama^+^ isomers. Specifically, all frequencies of the calculated modes of the most stable **(I–III)‐Ar(I)** isomers differ from those of the **I–III** monomers by less than 4 cm^−1^ in the fingerprint range, 9 cm^−1^ in the *ν*_CH_ range, and 5 cm^−1^ in the *ν*_NH_ stretch range. Anharmonic calculations confirm the given assignment and show very good correspondence between bands **H** and **I** (3151 and 3267 cm^−1^) with 2*β*_NH2_ of **I‐Ar(I)** and **III‐Ar(I)** (3150 and 3254 cm^−1^), respectively. Significantly, band **I** cannot be assigned to a *ν*_NH_ fundamental which is red‐shifted by Ar‐tagging but must be explained by an 2*β*_NH2_ overtone band because of its blue‐shift upon sequential Ar solvation and when going from Ar to N_2_. In summary, the IRPD spectra of Ama^+^Ar_1‐3_ show the clear signature of at least three different monomers in the *ν*_NH_ range and provide a very good approximation of the IR absorption spectrum of bare Ama^+^. Overall, the largest complexation shifts in the IRPD spectra of Ama^+^Ar_*n*_ occur for *n*=1 and 2, and this result is confirmed by the calculations with the two H‐bonded Ar ligands for *n*=2. As expected, the less strongly N‐bonded third Ar ligand has less impact on both the measured and computed IR spectra.

## Ama^+^N_2_ dimer

**Figure 6:**
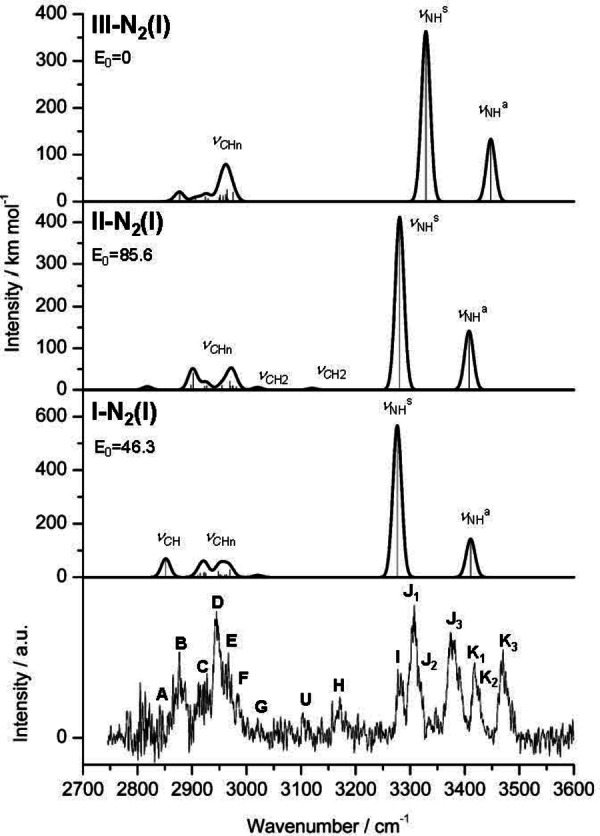
IRPD spectrum of Ama^+^N_2_ in the XH stretch range compared to linear IR absorption spectra of **(I–III)‐N_2_** (**I**) calculated at the B3LYP−D3/cc‐pVTZ level. The positions of the transition observed and their vibrational and isomer assignment are listed in Table S4. Relative energies (*E*_0_) are given in kJ mol^−1^.

Unlike Ar ligands, attachment of N_2_ has a stronger effect on the monomer structures due to its larger proton affinity (PA=494 vs. 369 kJ mol^−1^) and additional quadrupole moment.^[33]^ Because of the anisotropy of the charge‐quadrupole and charge‐induced dipole interaction, N_2_ ligands approach a positive charge in a linear configuration.[^25a^, ^25c^, ^32^] Due to the higher proton affinity, the trend of N_2_ to form nearly linear NH…N_2_ H‐bonds to the NH_2_ group is much more pronounced than for Ar. As a consequence, these H‐bonded isomers are the global minima on the **(I–III)‐N_2_** potentials, with *D*_0_=13.4, 15.7, and 13.9 kJ mol^−1^ for **(I–III)‐N_2_(I)**. These are substantially more stable than those isomers in which N_2_ simply binds to the C_10_H_15_ moiety, with *D*_0_=6.6–9.3, 8.4–10.1, and 7.0‐9.8 kJ mol^−1^ for **I‐N_2_(II–V)**, **II‐N_2_(II–V)**, and **III‐N_2_(II–VII)**, respectively. Thus, the H‐bonded global minima are well separated from the van‐der‐Waals local minima, with energy gaps of Δ*E*_0_=4.1, 5.6, and 4.1 kJ mol^−1^ for **I–III**, respectively. To this end, we discuss here only the most stable minima **(I–III)‐N_2_(I)** (Figure 1) and their IR spectra are compared in Figure 6 to the measured IRPD spectrum (Table S4).

Compared to Ar, N_2_ is bound to the NH_2_ group via stronger and more linear intermolecular H‐bonds (*R*_NH…N_=2.162, 2.144, 2.144 Å and *ϕ*_NHN_=179.1°, 178.3°, 170.5° for **I–III**) and larger charge transfer to the ligand (Δ*q*=14, 14, 11 m*e*). The partial charge on the NH proton donor atoms is increased (by 12, 10, 11 m*e*), leading to a substantial increase of the IR intensities in the *ν*_NH_ modes by a factor of 2–3, an effect typical for H‐bonding. The N−H proton donor bonds are elongated by 3–4 mÅ, resulting in red‐shifts in the *ν*_NH_^s^ (46, 48, 30 cm^−1^) and *ν*_NH_^a^ modes (28, 25, 19 cm^−1^). These predicted red‐shifts are somewhat larger than those observed experimentally with respect to the Ama^+^Ar spectrum (Δ**J_1‐3_** =14, 29, 9 cm^−1^; Δ**K_1‐3_** =8, 24, 10 cm^−1^) but reproduce the qualitative trend. Part of this discrepancy arises from the neglected experimental shifts upon Ar complexation, while the remaining part may come from an overestimation of the computed harmonic shifts.[^26f^, ^26g^, ^26i^, ^26j^] According to the given assignment to all three isomers **I–III**, the two bands **J_2_** and **K_2_** assigned to **II** are difficult to observe due to their larger red‐shift into the shoulders of **J_1_** and **K_1_**. When considering the widths and asymmetric band profiles of these bands and the larger discrepancy between observed and predicted shift, it would be also conceivable that isomer **II** is not present in the Ama^+^N_2_ spectrum at all. However, the very weak band **U** at 3103 cm^−1^, which is not observed in the Ama^+^Ar_*n*_ spectra, agrees well with the *ν*_CH2_^a^ mode of **II‐N_2_(I)** predicted at 3120 cm^−1^ and thus may be considered as a marker for the presence of **II**. Due to the larger retarding force, H‐bonded N_2_ causes a larger blue‐shift in *β*_NH2_ upon complexation than Ar (by 6, 7, 8 cm^−1^ for **I–III**), and this result is nicely reproduced by the measured blue‐shifts of the 2*β*_NH2_ overtones of isomers **I** and **III**, assigned to bands **H** (+21 cm^−1^) and **I** (+15 cm^−1^). As the *ν*_CH*n*_ modes are nearly identical to the corresponding monomer modes, peaks **A‐G** of the N_2_ spectrum can be identified analogous to the Ar spectrum.

## Ama^+^H_2_O dimer

**Figure 7:**
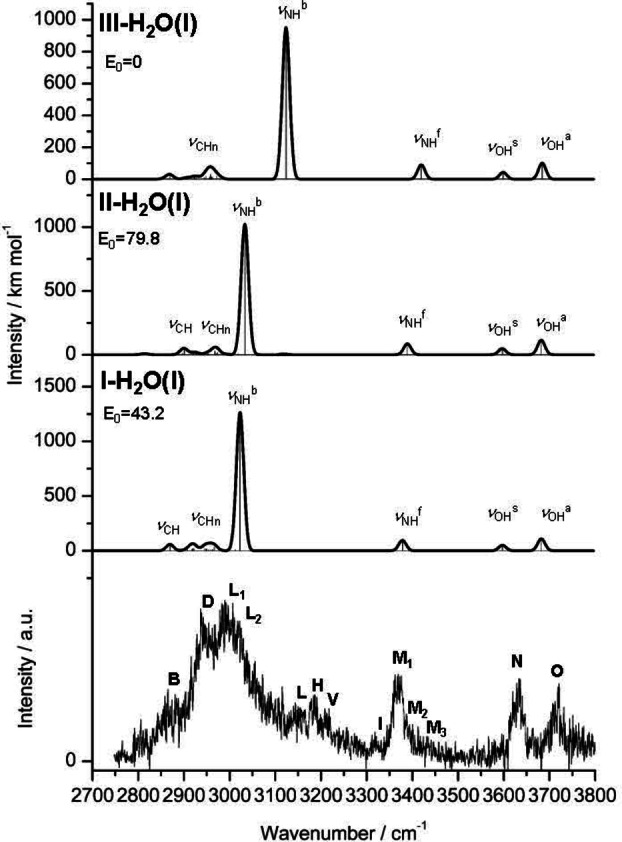
IRPD spectrum of Ama^+^H_2_O in the XH stretch range compared to linear IR absorption spectra of **(I–III)‐H_2_O(I)** calculated at the B3LYP−D3/cc‐pVTZ level. The positions of the transition observed and their vibrational and assignment are listed in Table S5. Relative energies (*E*_0_) are given in kJ mol^−1^.

The interaction between Ama^+^ and H_2_O is much stronger than with Ar and N_2_, because H_2_O has a much larger proton affinity (PA=691 kJ mol^−1^)^[33]^ and an additional large dipole moment, giving rise to strong electrostatic cation‐dipole attraction. Again, H_2_O prefers binding to the acidic NH_2_ group rather than to the C_10_H_15_ moiety, which has already been reported for the cage isomer **I** in our previous study of microhydrated Ama^+^(H_2_O)_1‐3_ clusters.^[24]^ Binding of H_2_O to isomers **II** and **III** is reported here for the first time and compared with new experimental IRPD data (Figure 7). Calculated energies of all considered Ama^+^H_2_O isomers are listed in Table S12 and their IR spectra are shown in Figure S31–S33. All isomers have a favorable charge‐dipole configuration with the electronegative O atom of H_2_O pointing toward the positive charge of **I–III**. Like for N_2_, the binding energies of the H‐bonded global minima, *D*_0_=61.4, 66.4, and 58.7 kJ mol^−1^ for **(I–III)‐H_2_O(I)**, are much higher than those with H_2_O attached to the C_10_H_15_ moiety, *D*_0_=33.6, 42.0, and 39.1 kJ mol^−1^ for **(I–III)‐H_2_O(II)**, leading to large energy gaps between NH‐bonded and CH‐bonded isomers (Δ*E*_0_=27.8, 24.4, 19.5 kJ mol^−1^ for **I–III**). Hence, we discuss only the **(I–III)‐H_2_O(I)** global minima with the strong NH…O ionic H‐bonds further (Figure 1), and their IR spectra are compared in Figure 7 to the measured IRPD spectrum (Table S5).

In all **(I–III)‐H_2_O(I)** global minima, H_2_O forms a nearly linear and very strong and short NH…O ionic H‐bond to the NH_2_ group (*R*_NH…O_=1.77, 1.77, 1.82 Å and *ϕ*_NHO_=170.5°, 171.6°, 169.7° for **I–III**). As a result of the very strong H‐bonds, the *ν*_NH_^s/a^ normal modes of the NH_2_ group of the monomers decouple into free and bound NH stretch modes (*ν*_NH_^f/b^) upon monohydration. The N−H proton donor bonds of **I** and **II** are substantially elongated (Δ*r*_NH_=21 and 20 mÅ), leading to massive red‐shifts in the *ν*_NH_^b^ frequencies down to 3023 and 3033 cm^−1^, along with drastic enhancements in the IR intensities (factor 6 and 8 compared to *ν*_NH_^s^). The N−H bond of **III** is less stretched than in **I** and **II** (Δ*r_NH_* =16 mÅ), leading to a smaller red‐shift down to *ν*_NH_^b^=3123 cm^−1^ with an intensity increase of factor 5. The free N−H bonds hardly change, resulting in less intense free *ν*_NH_^f^ modes at 3378, 3389, and 3420 cm^−1^ for **I–III**. Charge transfer to H_2_O is significantly higher than for N_2_ and Ar (Δ*q*=38, 41, 33 m*e*). However, due to the low ionization energy of Ama (IE=8.6 vs. 12.6 eV)[^16b^, ^16c^] and its open‐cage isomers, and the higher proton affinity of the amantadinyl radical and its open‐cage isomers (PA=960, 980, 1010 kJ mol^−1^ for removing H^+^ from the NH_2_ group) as compared to H_2_O (PA=691 kJ mol^−1^),^[33]^ the positive charge remains mostly on the C_10_H_15_NH_2_ moiety and no proton transfer to H_2_O occurs upon monohydration. As already noted previously, H_2_O attachment to **I** has a significant impact also on the structure of the adamantyl cage.^[24]^ For example, the C−N bond is slightly elongated by 8 mÅ and the C1−C3 bond contracts by 39 mÅ. As a result, the lowest *ν*_CH_ mode is blue‐shifted by 29 cm^−1^ to 2869 cm^−1^, while the other *ν*_CH/CH2_ frequencies do not change much. The impact of H_2_O on structure **II** is much smaller. The C=N double bond contracts by 4 mÅ and the C1…C3 distance of the broken bond shortens by 10 mÅ. The *ν*_CH_ mode is only slightly red‐shifted to 2814 cm^−1^, and the remaining *ν*_CH/CH2_ frequencies hardly change. H_2_O attachment to **III** results in a contraction of the C=N double bond by 8 mÅ and a larger increase in the C1…C3 distance by 51 mÅ, but again the *ν*_CH/CH2_ frequencies change only slightly.

As discussed previously, comparison of the IRPD spectrum of Ama^+^H_2_O with the spectrum predicted for **I‐H_2_O(I)** demonstrates that all major bands in the experimental spectrum can be explained by this isomer.^[24]^ Briefly, the red‐shifted *ν*_NH_^b^ mode (3023 cm^−1^) and free *ν*_NH_^f^ mode (3378 cm^−1^) of **I‐H_2_O**(**I**) are clearly observed at 2990 (**L_1_**) and 3368 cm^−1^ (**M_1_**). Band **B** at 2865 cm^−1^ is assigned to *ν*_CH_ (2869 cm^−1^) and band **D** at 2937 cm^−1^ to *ν*_CH2_ modes (2919 and 2957 cm^−1^). Peaks **N** and **O** at 3627 and 3717 cm^−1^ in the free OH stretch range are readily assigned to *ν*_OH_^s^ (3597 cm^−1^) and *ν*_OH_^a^ (3682 cm^−1^) of **I‐H_2_O**(**I**). The bands **H** and **V** at 3187 and 3213 cm^−1^ are probably arising from 2*β*_NH2_ and 2*β*_OH2_ overtones of **I‐H_2_O(I)** predicted at 3176 and 3238 cm^−1^. An anharmonic calculation supports this assignment with calculated frequencies of 3187 and 3211 cm^−1^, respectively.

For the remaining bands in the Ama^+^H_2_O spectrum two scenarios emerge. In scenario I, all bands are assigned to H_2_O clusters of isomer **I**. Then, bands **I** (3318 cm^−1^), **M_2_** (3387 cm^−1^), and **M_3_** (3433 cm^−1^) may be assigned to the free *ν*_NH_ modes of **I‐H_2_O** isomers with a completely free NH_2_ group, such as **I‐H_2_O**(**II**) and **I‐H_2_O**(**III**) with *ν*_NH_^s^=3329 and 3326 cm^−1^ and *ν*_NH_^a^=3445 and 3326 cm^−1^, respectively (Figure S31). Assuming that only the two most stable isomers **I‐H_2_O(I)** and **I‐H_2_O(II)** are present, one can roughly estimate their population ratio as 10 : 1 from the integrated peak intensities of peaks **M_1_** and **I** and the calculated IR cross sections, and assuming similar dissociation efficiencies for H_2_O elimination. Although we cannot exclude scenario I, we consider it unlikely because of the high relative energy gap between **I‐H_2_O**(**I**) and **I‐H_2_O(II/III)** of 27.8 and 30.9 kJ mol^−1^, and the clear detection of the open‐cage isomers **II** and **III** in the IRPD spectra of Ama^+^Ar_1‐3_ and Ama^+^N_2_.

Following our favored scenario II, we attribute bands **L_2_** and **M_2_** at 3021 and 3387 cm^−1^ to *ν*_NH_^b^ and *ν*_NH_^f^ of **II**‐**H_2_O**(**I**) predicted at 3033 and 3389 cm^−1^. Moreover, the broad and less intense features **L_3_** and **M_3_** at 3147 and 3433 cm^−1^ are indicative of a small population of **III‐H_2_O(I**), with computed values of *ν*_NH_^b^=3123 cm^−1^ and *ν*_NH2_^f^=3420 cm^−1^. The population of **II‐H_2_O(I)** and **III‐H_2_O(I)** can then be roughly estimated as ∼30 % and ∼10 % of **I‐H_2_O(I)** by considering the calculated and observed IR intensities of peaks **M_1‐3_**. The overall population ratio for **I–III** of 10 : 3 : 1 for the H_2_O‐tagged cations may be compared to that derived for the Ar‐tagged cations (10 : 3 : 7), indicating similar relative abundance for **II** but a rather reduced population for **III**, a conclusion rationalized below by considering the potential energy diagram for ring opening and 1,2 H‐shift. In scenario II, the bands **B**, **D**, **N**, and **O** mostly assigned to **I‐H_2_O(I**) will also contain contributions from the corresponding modes of **II/III‐H_2_O(I**). Compared to Ar and N_2_, bands **H** (3187 cm^−1^) and **I** (3318 cm^−1^) of the H_2_O spectrum are shifted further to the blue, coherent with their assignment to 2*β*_NH2_ of **I‐H_2_O(I)** and **I‐H_2_O(III)**, respectively. Finally, band **V** (3213 cm^−1^) can be assigned to 2*β*_OH2_ of **(I–III)‐H_2_O(I)** predicted at 3238, 3204 and 3203 cm^−1^, which explains why this peak **V** is observed only in the Ama^+^H_2_O spectrum.

The computed binding energies of *D*_0_∼60 kJ mol^−1^ (∼5000 cm^−1^) for the assigned Ama^+^H_2_O isomers substantially exceed the IR photon energy employed for single‐photon IRPD (<4000 cm^−1^). Consequently, the measured spectrum is produced by internally hot cluster ions that contain an internal energy of at least 1000 cm^−1^. As a result, the IRPD spectrum of Ama^+^H_2_O does not reflect the fundamental transitions (*ν*) of cold clusters but sequence hot bands of the type *ν*+*ν*_x_←*ν*_x_, where *ν*_x_ are low‐frequency inter‐ and intramolecular modes. This effect causes the widths of the Ama^+^H_2_O transitions to be broader than those of Ama^+^Ar_*n*_ and Ama^+^N_2_ for which h*ν*>*D*_0_ (Table 1, Figure 3).

## Comparison between Ama^+^L

**Figure 8:**
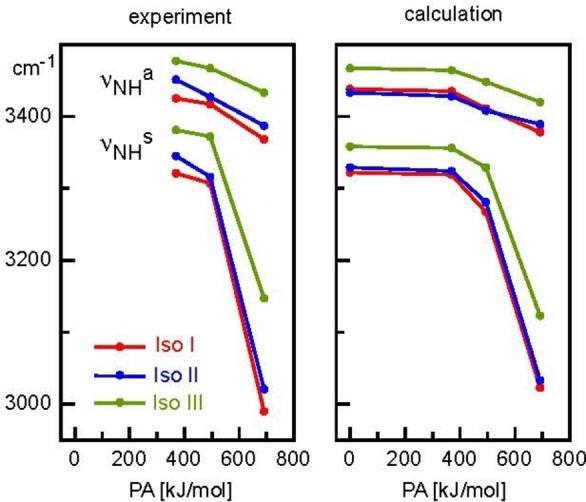
Experimental and calculated frequencies (in cm^−1^, B3LYP−D3/cc‐pVTZ) of the *ν*_NH_^s/a^ and *ν*_NH_^b/f^ modes of **I–III** and **(I–III)‐L** with L=Ar, N_2_, and H_2_O as a function of the proton affinity of the ligands.

In addition to the nascent cage isomer **I** of Ama^+^, the analysis of the IRPD spectra of Ama^+^Ar_1‐3_ by DFT calculations provides clear evidence for the formation of the open‐cage structures **II** and **III**, when using an electron ionization energy of up to 200 eV. The presence of six peaks in the *ν*_NH_ range (**J_1‐3_**, **K_1‐3_**) and three intense bands in the *β*_NH2_ range (**Q_1‐3_**) clearly indicates the existence of (at least) three different monomer isomers. Moreover, **I–III** can also be assigned in the Ama^+^L spectra with L=N_2_ and H_2_O. All three isomers differ strongly by the structural and vibrational properties of their NH_2_ groups, which serve as sensitive probe of their structure. The B3LYP−D3 calculations identify **I** as the canonical cage radical cation, while **II** and **III** are bicyclic distonic iminium ions. The IRPD spectra clearly indicate that the NH_2_ group is preserved in all three isomers upon ionization and structural rearrangements. This acidic and partially positively charged NH_2_ group is in all three isomers the preferred binding site for neutral ligands, while the C_10_H_15_ moiety is less attractive. The preferred cluster growth is thus solvation of the NH_2_ group by formation of one or two NH…L ionic H‐bonds. For L=Ar, these H‐bonds are very weak and rather nonlinear because they compete with weak CH…L van‐der‐Waals contacts to the C_10_H_15_ moiety. In contrast, the NH…N and NH…O H‐bonds are much stronger because of the additional electrostatic interactions arising from quadrupole and dipole moments of N_2_ and H_2_O, respectively. As a result, these H‐bonds are rather linear, with no or little interaction of L with the C_10_H_15_ unit. For example, the interaction strength in **I–L** increases in the order Ar < N_2_ < H_2_O as *D*_0_=8.4<13.4<61.4 kJ mol^−1^, in line with the proton affinity of the ligands (PA=369<494<691 kJ mol^−1^). As a result, the intermolecular NH…L bonds get shorter (2.72>2.14>1.77 Å) and charge transfer from **I** to L increases (6<14<38 m*e*). Because of the stronger intermolecular bond, the perturbation on the NH_2_ group gets stronger, with larger N−H bond elongations (Δ*r*_NH_=1<4<21 mÅ), and the proton donor NH stretch frequency experiences increasing red‐shifts down to 3322, 3276, and 3023 cm^−1^. In general, the interaction of **II** with L is slightly stronger than with **I** and **III** for all three ligands (*D*_0_=8.4<8.9>8.8, 13.4<15.7>13.9, 61.4<66.4>58.7 kJ mol^−1^ for Ar, N_2_, H_2_O), but overall the variation of the ligand binding affinities is small. This is fully consistent with the charge on the acidic NH protons (*q*_H_=0.405<0.421>0.411 *e*) and the NH…L bond lengths. Figure 8 compares the computed and experimental NH stretch frequencies for all investigated Ama^+^L clusters and the overall good qualitative and quantitative agreement supports the given vibrational and isomer assignments. When compared to other NH…L ionic H‐bonds, such as those in clusters of small ions (e. g., NH_2‐4_^+^, N_2_H^+^)^[34]^ or aromatic amines (e. g., anilines, protonated pyrimidine),[^25^, ^26a^, ^26b^, ^26f^, ^26g^, ^26i^, ^26j^] the NH…L bonds in **(I–III)‐L** are significantly weaker and less directional, mainly due to the lower acidity of the NH_2_ group resulting from the large charge delocalization into the C_10_H_15_ moiety and the high PA of the C_10_H_15_NH radicals.

## Potential energy profile for cage opening in Ama^+^ and Ama^+^L

In the following, we discuss the process of ionization, cage‐opening reaction, subsequent 1,2 H‐shift, the corresponding potential energy surface and its dependence on the solvent. Neutral Ama is ionized in the high‐pressure region of the supersonic plasma expansion of the EI source by electron and/or chemical ionization. To this end, electrons hit the expansion gas with a kinetic energy of up to 200 eV, leading to a variety of processes including elastic and inelastic scattering, where the electrons may lose kinetic energy and also produce secondary electrons. At some point, direct EI of Ama (IE<10 eV)^[16b]^ with primary or secondary electrons occurs, which may compete with EI or electronic excitation of carrier gas atoms and ligand molecules which may then ionize Ama in collisions by exothermic charge transfer and/or Penning ionization. When Ama is ionized by fast electrons, higher excited states may be populated, which decay very fast by internal conversion into the ground electronic state or fragment. Part of the produced nascent Ama^+^ population (isomer **I** will be initially produced due to vertical ionization processes) will then cool down by collisions in the expansion to form cold ions **I**. Some part has enough energy to overcome the barriers for cage opening and 1,2 H‐shift and will then cool down to generate cold ions **II** and **III**. As a final step, the cold ions will form clusters by three‐body aggregation. Cluster formation may also take place during the isomerization reaction and the cooling process. A further route, which is particularly feasible for H_2_O, is the formation of larger neutral Ama(H_2_O)_*n*_ clusters in the expansion, which are then ionized and cool down via collisions and/or ligand evaporation. For the latter pathway, it is also necessary to consider the potential of the clusters for the ionization‐induced rearrangement reaction. To summarize, experimentally the energetics and kinetics of the processes occurring in the plasma expansion are not well defined.

The potential for the reaction of the monomer is shown in Figure 2. Briefly, Ama is vertically ionized by electron removal from the N lone pair (HOMO) to produce the radical cation **I** (*E*_0_=45.9 kJ mol^−1^). As a result, the pyramidal NH_2_ group becomes planar and due to increased delocalization of the N lone pair orbital, the C−N bond order increases from 1 to ∼1.5, and the adjacent C1−C3 bond is slightly activated, which is the first step for opening the cage. The HOMO of **I** is delocalized over the whole cation (Figure S35), while most charge remains on the CNH_2_ group (0.528 *e*). For highly energetic **I**, it is possible to overcome the barrier for breaking the C1−C3 bond and opening the cage via **TS(I–II)** (*V*_b_=54.1 kJ mol^−1^, *E*_0_=100.0 kJ mol^−1^), accompanied by a chair→boat conversion of the cyclohexane ring carrying the NH_2_ group. This process results in a separation of charge and spin, so that the resulting shallow minimum **II** is a bicyclic distonic iminium ion (*E*_0_=87.4 kJ mol^−1^), with most of the positive charge located on the CNH_2_ group (0.754 *e*) and the spin located on the primary CH_2_⋅ radical center at C3 (*s*_C3_=1005). The HOMO of **II** is mostly the 2*p*_z_ orbital of the CH_2_⋅ group with an sp^2^ C atom (Figure S35). In the final 1,2 H‐shift to the CH_2_⋅ center, the much more stable tertiary CR_3_⋅ radical center (*s*_C9_=0.627) is formed via a barrier of *V*_b_=81.6 kJ mol^−1^ (*E*_0_=169.0 kJ mol^−1^), accompanied by a boat→chair back conversion of the cyclohexane ring, resulting in maximum stabilization of the radical in the distonic bicyclic iminium ion **III** (*E*_0_=0), which is by far the global minimum on the Ama^+^ potential. The HOMO of **III** is almost completely delocalized again over the entire cation, with some substantial orbital overlap between the 2*p*_z_ orbital of the C9 radical center and the 2*pπ* orbital of the C=N double bond (Figure S35). The partial charge of the CNH_2_ group is reduced to 0.608 *e*, because some of the charge is transferred to the tertiary C9 atom, but it is still slightly higher than in the initial structure **I** (0.528 *e*).

As the Ar and N_2_ binding energies are very small (<16 kJ mol^−1^) compared to the energies involved in the potential energy profile with respect to barriers and relative energies of minima (Figure S34), these ligands are considered to have negligible influence on reaction efficiencies and the final population of the isomers **I–III**. In particular, they do not change the energetic order of **I–III**. As the ligand binding energies are similar for each **I–III** isomer, the potential energy profile for the Ama^+^Ar dimers are very similar to that of bare Ama^+^, and we may reliably estimate from the relative isomer population in these clusters directly the isomer abundances of the monomers **I–III**. The population ratio of 10 : 3 : 7 for **I–III** derived from the *β*_NH2_ intensities of the Ama^+^Ar spectrum are fully consistent with the potential energy profile predicted for the Ama^+^ monomer. The high abundance of **I** is due to its initial preparation by vertical ionization and the high barrier required for isomerization toward **II** and **III** of 54.1 and 123.1 kJ mol^−1^, respectively. Thus, it is likely that a substantial population of Ama^+^ is trapped in the deep initial well by rapid collisional cooling despite of its high relative energy. The minor population of **II** is attributed to its shallow potential well (12.6 kJ mol^−1^), while the higher population of **III** is rationalized by its highest stability. In addition, tunneling of H through the short barrier between **II** and **III** may also enhance the population of **III**. As illustrated in Figure 2, the potential energy profile computed for Ama^+^H_2_O differs strongly from that of Ama^+^ (and Ama^+^Ar/N_2_). While the relative energies of the **I–III** minima and **TS(I–II)** are similar, **TS(II–III)** is much higher for Ama^+^H_2_O (*E*_0_=213 vs. 169 kJ mol^−1^), which suppresses the formation of **III**. Indeed, comparison of the population ratio inferred from the Ama^+^H_2_O spectrum (10 : 3 : 1 for **I–III**) compared to that derived from Ama^+^Ar (10 : 3 : 7) fully confirms this view.

## Comparison to Ada^+^

It is instructive to compare the properties of Ama^+^ with those of Ada^+^ to extract the impact of H→NH_2_ substitution on the ionization‐triggered cage‐opening reaction. To this end, we calculated the structures, energies, charge and spin distributions, HOMO orbitals, and IR spectra of the three Ada^+^ isomers **I–III**, along with the potential for the rearrangement reaction at the same computational level for direct comparison with Ama^+^ (Figure S4, S36–37). Our results for the Ada^+^ potential are consistent with previous calculations,^[19e]^ and the potential is compared in Figure 2 to the one of Ama^+^. As can be seen, while the energetic order of the isomers **I–III** is the same for both Ama^+^ and Ada^+^, their relative energies and reaction barriers change drastically. Significantly, substitution of H by the NH_2_ group qualitatively changes the ionization process. While in Ada^+^, ionization occurs from a bonding CC σ orbital completely delocalized over the whole C_10_H_16_ cage,^[19g]^ ionization of Ama^+^ occurs from the N lone pair of the NH_2_ group, which is only partly delocalized into the cage. As a result, the ionization energy of Ama is largely reduced compared to that of Ada (exp: 8.6 vs. 9.25 eV, B3LYP−D3: 7.88 vs. 8.81 eV).[^16b^, ^16c^] Isomer **I** of Ada^+^ (*E*_0_=5.4 kJ mol^−1^) undergoes Jahn‐Teller distortion from *T*_d_ to *C*_3v_ symmetry, which leads to an elongation of the three C−C bonds parallel to the *C*_3_ axis and a substantial activation of the C−H bond lying on this axis. This very acidic C−H bond with its high positive charge (*q*_H_=0.354 *e*) is an attractive proton donor for H‐bonding with neutral ligands, such as He, Ar, N_2_, and H_2_O.[^19g^, ^20^] Significantly, while the electron‐donating NH_2_ group in isomer **I** of Ama^+^ strongly activates the C1−C3 bond (*r*_C1C3_=1.697 Å), which eventually breaks, no such activation is observed for ionization of Ada^+^ (*r*_C1C3_=1.511 Å). Hence, the barrier for breaking this bond is much higher for Ada^+^ than for Ama^+^ (*V*_b_=156.7 vs. 54.1 kJ mol^−1^), i. e., H→NH_2_ substitution strongly facilitates cage opening. Similar to Ama^+^, isomer **II** of Ada^+^ is located in a shallow potential well (9 vs. 13 kJ mol^−1^) and thus much higher in relative energy (153.0 vs. 87.4 kJ mol^−1^). It is also a distonic ion with a CH_2_⋅ radical group (*s*=0.797) separated from the positive charge (Figure S11–S12), mostly located on the opposite CH_2_‐CH‐CH_2_ moiety (*q*_CH_=0.623 *e*). The energy barrier for the 1,2 H‐shift at **TS(II–III**) is much lower compared to Ama^+^ (*V*_b_=37.8 vs. 81.6 kJ mol^−1^) even though the relative energy to the global minimum **III** is higher (*E*_0_=190.8 vs. 169.0 kJ mol^−1^). Similar to **III** of Ama^+^, the HOMO of the global minimum **III** of Ada^+^ is again delocalized over the entire cation (Figure S36). The charge of the CH_2_‐‐CH‐‐CH_2_ group is reduced to 0.337 *e*, because a large amount of charge is now located at the tertiary radical C‐atom (*q*_C_=0.410 *e*).

In summary, H→NH_2_ substitution of Ada has a drastic impact on the potential for the cage‐opening reaction and subsequent 1,2 H‐shift. The barrier for this reaction is drastically reduced for Ama^+^ and thus all three isomers **I–III** are detected in the IRPD spectra of Ama^+^L with L=Ar, N_2_, and H_2_O. In contrast, the high barrier for this process in Ada^+^ explains why such a reaction was not detected in previous IRPD spectra of Ada^+^L with the same ligands.[^19g^, ^20^] In previous work on Ada^+^L, cage opening was not considered but the comparison of the IR spectra computed for **I–III** of Ada^+^ with the IRPD spectrum of Ada^+^He_2_ in the CH stretch range (Figure S37) clearly demonstrates that the whole spectrum can be assigned to **I**, while the population of **II** and **III** are clearly below the detection limit, in accordance with the potential shown in Figure 2.

## Concluding Remarks

In summary, the structural isomers of the Ama^+^ cation and their solvated clusters with Ar, N_2_, and H_2_O are probed in a molecular beam by IRPD in the sensitive XH stretch and fingerprint ranges. The analysis of the IRPD spectra by DFT calculations provides direct evidence for the ionization‐induced opening of the adamantyl cage followed by an 1,2 H‐shift, and three distinct minima **I–III** are identified on the potential energy profile by the unique *ν*_NH_ and *β*_NH2_ modes of their rather different NH_2_ groups. Vertical electron ionization of Ama produces the canonical nascent cage isomer **I** of Ama^+^, which then via substantial barriers can react in a unimolecular reaction toward two distonic bicyclic iminium ions **II** and **III**. Significantly, isomer **III** with a tertiary radical center is the global minimum and thus more stable than the canonical cage structure **I**. In addition to the geometric and vibrational properties of the three isomers, details of the potential energy profile are revealed. The relative abundance of **I–III** are fully consistent with the shape of this potential energy profile, with respect to both the relative energies of the minima and the barriers for reaction. Although only a local minimum on the potential, the nascent isomer **I** is most abundant because it is directly produced by vertical ionization of the neutral cage molecule and largely trapped in its deep potential by collisional cooling. Part of the energetic population of **I** can overcome the high barriers toward **II** and **III**, whereby the well of **II** is quite shallow leading to its smallest population, while the substantial population of **III** is consistent with its highest stability. Tunneling of H through the short barrier between **II** and **III** may also enhance the population of **III**, a hypothesis which may be tested using partially deuterated samples in the future. Solvation of the ions with Ar and N_2_ has little impact on the potential energy profile and isomer population, while the production of **III** is strongly suppressed for solvation with H_2_O due to a large increase in the barrier for the **II**→**III** reaction. Comparison between Ama^+^ and Ada^+^ reveals that H→NH_2_ substitution has a drastic impact on the geometric and electronic structure of the three isomers **I–III** due to the strong electron‐donating character of the NH_2_ group. The resulting orbital conjugation drastically reduces the barriers on the potential compared to Ada^+^, for which the much higher barriers have prevented the detection of the cage‐opening reaction in previous related experiments. To test this hypothesis, experiments with cyano‐adamantane are currently underway to study the effects of an electron‐withdrawing substituent (CN) on the cage‐opening potential energy profile. Significantly, the presented IRPD spectra of Ama^+^L_*n*_ provide the first experimental evidence for the cage‐opening and 1,2 H‐shift reaction for any diamondoid cation and thus yield valuable insight into their chemical reactivity. As the radical cations of diamondoids are frequently invoked in the mechanism for (substitution) reactions in polar solvents, the results of the current study provide valuable insight for developing new routes in organic synthesis in diamondoid chemistry. In addition, the intermediates **II** and **III** may also be used in novel routes of iminium ion chemistry in solution, a strategy which has not been exploited so far. The acidic NH_2_ group serves as the most attractive ligand binding site for all three ions **I–III** and the ligand‐ and size‐dependent frequency shifts of the *ν*_NH_ and 2*β*_NH2_ modes provide a quantitative probe of the type and strength of the Ama^+^…L interaction and the sequence of the cluster growth. In general, the first two ligands form equivalent NH…L ionic H‐bonds to the two available NH protons before other less stable binding sites are occupied.^[24]^ In all cases, the interaction with **II** is stronger than with **I** and **III** which correlates with the acidity of the NH_2_ group. For Ar ligands, the NH…Ar bonds are very weak and thus rather nonlinear because of competing CH…Ar contacts based on dispersion and induction forces. As a result, the Ama^+^Ar spectrum provides a very good approximation to the spectrum of bare Ama^+^. In contrast, stronger NH…N and NH…O ionic H‐bonds are present for clusters with N_2_ and H_2_O due to the additional electrostatic forces arising from the nonvanishing quadrupole and dipole moments, respectively. To this end, the choice of solvent has a drastic impact on the reactivity of the isomeric Ama^+^ ions in organic synthesis.

## Conflict of interest

There are no conflicts of interest to declare.

1

## Supporting information

As a service to our authors and readers, this journal provides supporting information supplied by the authors. Such materials are peer reviewed and may be re‐organized for online delivery, but are not copy‐edited or typeset. Technical support issues arising from supporting information (other than missing files) should be addressed to the authors.

Supporting Information

## Data Availability

The data that support the findings of this study are available in the supplementary material of this article.
