# Supplementary material for: Opening of the Diamondoid Cage upon Ionization Probed by Infrared Spectra of the Amantadine Cation Solvated by Ar, N2, and H2O
Source: Chemistry. 2022 Jun 20;28(44):e202200577. doi: 10.1002/chem.202200577 (PMC9400954; doi:10.1002/chem.202200577)
Supplement: Supplementary file 1 — Supporting Information [file CHEM-28-0-s001.pdf]

Chemistry–A European Journal

Supporting Information

Opening of the Diamondoid Cage upon Ionization Probed by Infrared Spectra of the Amantadine Cation Solvated by Ar, N_2, and H_2O

Martin Andreas Robert George and Otto Dopfer*

Table of Contents

Table S1. Computed vibrational frequencies (in cm^{-1}, B3LYP-D3/cc-pVTZ) of Ama compared to calculated vibrational frequencies of Ama^+(**I-III**). In bold are the convoluted peaks.

Table S2-S5. Computed vibrational frequencies (in cm^{-1}, B3LYP-D3/cc-pVTZ) compared to experimental values of Ama^+L (L=Ar, N_2, H_2O). In bold are the convoluted calculated band. The experimental values are given with peak width (fwhm in parenthesis) and are assigned to the most dominant vibrations. **S2**) Ama^+(**I-III**) (Figure 4); **S3**) Ama^+(**I-III**)-Ar(**I**) (Figure S15); **S4**) Ama^+(**I-III**)-N_2(**I**) (Figure 6); **S5**) Ama^+(**I-III**)-H_2O(**I**) and H_2O (Figure 7).

Table S6. Computed vibrational frequencies (in cm^{-1}, B3LYP-D3/cc-pVTZ) of $\nu_{NH}^{s/a}$ and $\nu_{NH}^{b/f}$ modes of Ama^+(**I-III**) and Ama^+(**I-III**)-L (L=Ar_{1-3}, N_2, H_2O) (Figure 8). IR intensities in km/mol are given in parentheses.

Table S7-S13. Various energies (kJ mol^{-1}) calculated at the B3LYP-D3/cc-pVTZ level. **S7**) Ama^+(**I-III**)-Ar, Ama^+(**I-III**)-N_2 and Ama^+(**I-III**)-H_2O; **S8**) Ama^+(**I-III**)-Ar_1(**I-VIII**); **S9**) Ama^+(**I-III**)-Ar_2(**I-VIII**); **S10**) Ama^+(**I-III**)-Ar_3(**I-VIII**); **S11**) Ama^+(**I-III**)-N_2(**I-VII**); **S12**) Ama^+(**I-III**)-H_2O(**I-IV**); **S13**) Ada^+(**I-III**).

Figure S1. Mass spectra of the EI ion source for an expansion of Ama seeded in 10-20 bar of Ar/N_2, along with assignments and Collision-induced dissociation (CID) spectra of size-selected Ama^+L to illustrate the loss of L as major fragmentation channel. Major fragment ions of Ama (m/z 151) at m/z 136, 108, 94, and 57 are indicated (F). a) Mass spectrum (10-100 u). b) Mass spectrum (100-200 u). c) CID spectrum of x→Ama^+. d) CID spectrum of Ama^+Ar →x. e) CID spectrum of Ama^+N_2 →x. f) CID spectrum of Ama^+H_2O →x.

Figure S2-S5. Calculated equilibrium structures (in Å and degrees) in their ground electronic state (B3LYP-D3/cc-pVTZ). **S2**) Ama and Ama^+(**I-III**); **S3**) Ama^+(**I-III**)-N_2(**I**), Ama^+(**I-III**)-H_2O(**I**) and H_2O; **S4**) Ada, Ada^+(**I-III**) and their transition states; **S5**) transition states of Ama^+ and Ama^+H_2O.

Figure S6-S13. NBO charge distribution (in me) or spin density distribution in their ground electronic states calculated at the B3LYP-D3/cc-pVTZ level. **S6**) Ama and Ama^+(**I-III**); **S7**) Ama and Ama^+(**I-III**) (spin); **S8**) Ama^+(**I-III**) and Ama^+(**I-III**)-Ar(**I**); **S9**) Ama^+(**I-III**)-Ar_2(**I**) and Ama^+(**I-III**)-Ar_3(**I**); **S10**) Ama^+(**I-III**)-N_2(**I**), Ama^+(**I-III**)-H_2O(**I**) and H_2O; **S11**) Ada, Ada^+(**I-III**) and their transition states; **S12**) Ada, Ada^+(**I-III**) and their transition states (spin); **S13**) transition states of Ama^+ and Ama^+-H_2O.

Figure S14. Calculated IR spectrum of Ama compared to the calculated IR spectra of Ama^+(**I-III**). Relevant modes are listed in Table S1.

Figure S15-S33. IRPD spectra of Ama^+L (L= Ar_{1-3}, N_2, H_2O) compared to linear IR absorption spectra of Ama^+(**I-III**)-L calculated at the B3LYP-D3/cc-pVTZ level. Differences in relative energy (E_0) are given in kJ mol^{-1}. **S15**) Ama^+(**I-III**)-Ar(**I**); **S16**) Ama^+(**I**)-Ar(**I-VIII**); **S17**) Ama^+(**II**)-Ar(**I-VIII**); **S18**) Ama^+(**III**)-Ar(**I-VIII**); **S19**) Ama^+(**I**)-Ar_2(**I-VIII**); **S20**) Ama^+(**II**)-Ar_2(**I-IX**); **S21**) Ama^+(**III**)-Ar_2(**I-X**); **S22**) Ama^+(**I**)-Ar_3(**I-VI**); **S23**) Ama^+(**I**)-Ar_3(**I-XIII**); **S24**) Ama^+(**II**)-Ar_3(**I-VI**); **S25**) Ama^+(**II**)-Ar_3(**I-XII**); **S26**) Ama^+(**III**)-Ar_3(**I-VI**); **S27**) Ama^+(**III**)-Ar_3(**I-XIII**); **S28**) Ama^+(**I**)-N_2(**I-V**); **S29**) Ama^+(**II**)-N_2(**I-V**); **S30**) Ama^+(**III**)-N_2(**I-VII**); **S31**) Ama^+(**I**)-H_2O(**I-III**); **S32**) Ama^+(**II**)-H_2O(**I-V**); **S33**) Ama^+(**III**)-H_2O(**I-III**).

Figure S34. Potential energy surfaces (minima and transition states) of Ama^+ (red), Ama^+Ar (green), Ama^+H_2O (blue) and Ama^+N_2 (violett) for the cage-opening reaction upon ionization of Ama and Ada, respectively. Differences in relative energy (E_0) are given in kJ mol^{-1}.

Figure S35-S36. HOMO orbitals of evaluated at the B3LYP-D3/cc-pVTZ level. **S35**) Ama and Ama^+(**I-III**) **S36**) Ada and Ada^+(**I-III**).

Figure S37. IRPD spectrum of Ama^+He_2 (2500-3300 cm^{-1}) compared to linear IR absorption spectra of Ada^+(**I-III**) calculated at the B3LYP-D3/cc-pVTZ level. Differences in relative energy (E_0) are given in kJ mol^{-1}.

Cartesian coordinates and energies of all structures

Table S1. Computed vibrational frequencies (in cm^{-1}, B3LYP-D3/cc-pVTZ) of Ama compared to calculated vibrational frequencies of Ama^+(**I-III**).[a] In bold are the convoluted peaks.

<table>
<tr><th>Mode of Ama [b]</th><th>Ama</th><th>Mode of Ama+ [d]</th><th>Ama+(I)</th><th>Ama+(II)</th><th>Ama+(III)</th></tr>
<tr><td rowspan="3">τ_{CH2}, γ_{CH2}</td><td rowspan="3">1256
1256 (4)
1274 (0.0)
1277 (0.2)
1279 (0.0)</td><td>τ_{CH2}, γ_{CH2}</td><td></td><td></td><td>1226
1218 (2)
1227 (10)</td></tr>
<tr><td>ρ_{NH2}, τ_{CH2}, γ_{CH2}</td><td>1226
1226 (22)
1243 (0.001)</td><td></td><td></td></tr>
<tr><td>γ_{CH2}</td><td></td><td>1247 (22)</td><td></td></tr>
<tr><td rowspan="2">γ_{CH2}</td><td rowspan="2">1308
1308 (5)
1311 (1)
1313 (1)
1319 (0.0)
1320 (0.0)</td><td>τ_{CH2}, γ_{CH2}</td><td>1274
1254 (1.3)
1271 (0.01)
1273 (0.1)
1274 (26)
1296 (5)
1306 (0.4)</td><td>1285
1283 (1)
1285 (4)</td><td>1301
1261 (1)
1280 (3)
1292 (1)
1301 (14)
1316 (5)</td></tr>
<tr><td>γ_{CH2}</td><td>1346
1320 (0.1)
1325 (2)
1345 (1)
1345 (2)
1352 (2)
1362 (0.2)</td><td>1310
1306 (3)
1311 (2)
1323 (1)
1330 (0.1)
1333 (0.2)
1350 (1)
1360 (0.2)</td><td>1336
1332 (4)
1338 (7)
1348 (2)</td></tr>
<tr><td rowspan="2">τ_{CH2}, γ_{CH2}, τ_{NH2}</td><td rowspan="2">1360
1342 (0.2)
1351 (1)
1358 (3)
1364 (2)
1373 (0.1)</td><td>ρ_{NH2}, γ_{CH2}, γ_{CH3}</td><td></td><td></td><td>1372
1363 (15)
1378 (12)
1380 (4)</td></tr>
<tr><td>ρ_{NH2}</td><td></td><td>1397 (10)</td><td></td></tr>
<tr><td>β_{CH2}</td><td>1461
1446 (0.0)
1448 (0.0)
1461 (8)
1461 (10)
1463 (8)
1486 (0.1)</td><td>β_{CH2}, β_{CH3}</td><td>1458
1435 (5)
1439 (1)
1450 (15)
1460 (12)
1463 (14)
1484 (1)</td><td>1441
1430 (1)
1438 (17)
1445 (20)

1469
1464 (3)
1469 (17)
1473 (3)</td><td>1440
1421 (6)
1430 (0)
1431 (16)
1442 (11)
1442 (28)
1447 (5)
1467 (5)</td></tr>
<tr><td rowspan="2">β_{NH2}</td><td rowspan="2">1609 (34)</td><td>ν_{CN}</td><td></td><td>1542 (4)</td><td>1500 (93)</td></tr>
<tr><td>β_{NH2}</td><td>1595 (70)</td><td>1670 (208)</td><td>1641 (216)</td></tr>
<tr><td rowspan="4">ν_{CH}, ν_{CH2}</td><td rowspan="4">2890
2880 (15)
2885 (28)
2890 (60)
2891 (4)
2892 (19)
2893 (28)

2917
2913 (113)</td><td>ν_{CH}</td><td>2840 (75)</td><td>2821 (8)</td><td></td></tr>
<tr><td>ν_{CH3}</td><td></td><td></td><td>2858 (0.3)</td></tr>
<tr><td>ν_{CH}, ν_{CH2}</td><td>2920
2908 (4)
2912 (16)
2921 (16)
2923 (20)
2925 (10)</td><td>2901
2898 (10)
2901 (40)</td><td>2878
2873 (2)
2878 (16)</td></tr>
<tr><td>ν_{CH}, ν_{CH2}</td><td>2955
2949 (5)
2950 (28)
2951 (1)
2952 (10)
2961 (6)
2962 (11)
2966 (4)
2971 (27)</td><td>2926
2916 (0.5)
2921 (8)
2929 (11)</td><td>2905
2898 (0)
2905 (9)</td></tr>
</table>

	2914 (68)<br>2916 (92)<br>2922 (28)<br>2926 (12)<br>2926 (46)<br>2932 (100)<br>2936 (5)<br>2941 (81)	ν_{CH3}			**2926**<br>2925 (10)<br>2930 (6)
		ν_{CH2}, ν_{CH3}		**2972**<br>2955 (0)<br>2956 (13)<br>2970 (25)<br>2971 (5)<br>2974 (8)<br>2974 (9)<br>2976 (8)	**2961**<br>2951 (5)<br>2953 (11)<br>2957 (11)<br>2959 (20)<br>2963 (7)<br>2965 (28)<br>2966 (4)<br>2977 (17)
		ν_{CH2}	**3024** (6)	**3021** (6)	
		ν_{CH2}		**3121** (4)	
ν_{NH}^{s}	**3285** (3)	ν_{NH}^{s}	**3322** (223)	**3329** (131)	**3358** (173)
ν_{NH}^{a}	**3359** (0.2)	ν_{NH}^{a}	**3438** (68)	**3433** (76)	**3467** (63)

[a] IR intensities in km mol^{-1} are given in parentheses. [b] Stretching (ν), bending (β), torsing (τ), wagging (γ), rocking (ρ).

Table S2. Computed vibrational frequencies (in cm^{-1}, B3LYP-D3/cc-pVTZ) of Ama^+(**I-III**) compared to experimental values of Ama^+Ar (Figure 4).[a] In bold are the convoluted calculated band. The experimental values are given with peak width (fwhm in parenthesis) and are assigned to the most dominant vibrations.

Mode [d]	Ama^+(**I**)	Ama^+(**II**)	Ama^+(**III**)	Ama^+-Ar Exp.
ρ_{NH2}, τ_{CH2}, γ_{CH2},	**1226**<br>1226 (22)<br>1243 (0.001)		**1226**<br>1218 (2)<br>1227 (10)	**R** 1228 (15)
γ_{CH2}		**1247** (22)		
τ_{CH2}, γ_{CH2}	**1274**<br>1254 (1.3)<br>1271 (0.01)<br>1273 (0.1)<br>1274 (26)<br>1296 (5)<br>1307 (0.4)	**1285**<br>1283 (1)<br>1285 (4)	**1301**<br>1261 (1)<br>1280 (3)<br>1292 (1)<br>1301 (14)<br>1316 (5)	
γ_{CH2}	**1346**<br>1320 (0.1)<br>1325 (2)<br>1345 (2)<br>1345 (1)<br>1352 (2)<br>1362 (0.2)	**1310**<br>1306 (3)<br>1311 (2)<br>1323 (1)<br>1330 (0.1)<br>1333 (0.2)<br>1350 (1)<br>1360 (0.2)	**1336**<br>1332 (4)<br>1338 (7)<br>1348 (2)	
ρ_{NH2}, γ_{CH2}, γ_{CH3}		**1397** (10)	**1372**<br>1363 (15)<br>1378 (12)<br>1380 (4)	**S** 1368 (35)
β_{CH2}, β_{CH3}	**1458**<br>1435 (5)<br>1439 (1)<br>1450 (15)<br>1460 (12)<br>1463 (14)<br>1484 (1)	**1441**<br>1430 (1)<br>1438 (17)<br>1445 (20)<br>**1469**<br>1464 (3)<br>1469 (17)<br>1473 (3)	**1440**<br>1421 (6)<br>1430 (0)<br>1431 (16)<br>1442 (11)<br>1442 (28)<br>1447 (5)<br>1467 (5)	**P** 1458 (25)
ν_{CN} [e]			**1500** (93)	**T** 1551 (7)
ν_{CN} [e]		**1542** (4)		
β_{NH2}	**1595** (70)			**Q_1** 1599 (6)
β_{NH2} [e]			**1641** (216)	**Q_2** 1664 (13)
β_{NH2} [e]		**1670** (208)		**Q_3** 1717 (7)
ν_{CH}		**2821** (8)		**A** 2842 (4)
ν_{CH}	**2840** (75)			**B** 2864 (11)
ν_{CH2}, ν_{CH3}			**2878**<br>2858 (0.3)<br>2873 (2)<br>2878 (16)	**B** 2864 (11)
ν_{CH2}, ν_{CH3}	**2920**<br>2908 (4)<br>2912 (16)<br>2921 (16)<br>2923 (20)<br>2925 (10)	**2901**<br>2898 (10)<br>2901 (40)<br>**2926**<br>2916 (0.3)<br>2921 (8)<br>2929 (11)	**2905**<br>2898 (0)<br>2905 (9)<br>**2926**<br>2925 (10)<br>2930 (6)	**C** 2920 (6)
ν_{CHn}	**2955**<br>2949 (5)<br>2950 (28)<br>2951 (1)<br>2952 (10)	**2972**<br>2955 (0)<br>2956 (13)<br>2970 (25)<br>2971 (5)	**2961**<br>2951 (5)<br>2953 (11)<br>2957 (11)<br>2959 (20)	**D** 2947 (25)<br>**E** 2959 (6)<br>**F** 2978 (4)

	2961 (6)<br>2962 (11)<br>2966 (4)<br>2971 (27)	2974 (8)<br>2974 (9)<br>2976 (8)	2963 (7)<br>2965 (28)<br>2966 (4)<br>2977 (17)	
ν_{CH2}	3024 (6)	3021 (6)		**G** 3008 (6)
ν_{CH2}		3121 (4)		
$2\beta_{NH2}$	3190[b]<br>3200 (12)[c]			**H** 3151 (9)
$2\beta_{NH2}$ [e]			3282[b]<br>3262 (0.7)[c]	**I** 3267 (8)
$2\beta_{NH2}$ [e]		3339[b]<br>3289 (0.3)[c]		
ν_{NH}^{s}	**3322** (223)			$\mathbf{J_1}$ 3321 (16)
ν_{NH}^{s}		**3329** (131)		$\mathbf{J_2}$ 3345 (8)
ν_{NH}^{s}			**3358** (173)	$\mathbf{J_3}$ 3381 (17)
ν_{NH}^{a}	**3438** (68)			$\mathbf{K_1}$ 3425 (9)
ν_{NH}^{a}		**3433** (76)		$\mathbf{K_2}$ 3451 (7)
ν_{NH}^{a}			**3467** (63)	$\mathbf{K_3}$ 3477 (11)

[a] IR intensities in km mol^{-1} are given in parentheses. [b] Harmonic value. [c] Result of anharmonic calculation.

[d] Stretching (ν), bending (β), torsing (τ), wagging (γ), rocking (ρ). [e] coupled β_{NH2}/ν_{CN} modes

Table S3. Computed vibrational frequencies (in cm^{-1}, B3LYP-D3/cc-pVTZ) of Ama^+(**I-III**)-Ar(**I**) compared to experimental values of Ama^+Ar (Figure S15).[a] In bold are the convoluted calculated band. The experimental values are given with peak width (fwhm in parenthesis) and are assigned to the most dominant vibrations.

Mode [d]	Ama^+(**I**)-Ar(**I**)	Ama^+(**II**)-Ar(**I**)	Ama^+(**III**)-Ar(**I**)	Ama^+-Ar Exp.
ρ_{NH2}, τ_{CH2}, γ_{CH2},	**1227**<br>1227 (20)<br>1246 (0.4)		**1227**<br>1220 (2)<br>1228 (9)	**R** 1228 (15)
γ_{CH2}		**1246** (21)		
τ_{CH2}, γ_{CH2}	**1275**<br>1255 (2)<br>1272 (3)<br>1274 (0.1)<br>1276 (22)<br>1296 (3)<br>1309 (1)	**1285**<br>1283 (1)<br>1285 (5)	**1302**<br>1260 (1)<br>1281 (3)<br>1292 (1)<br>1302 (14)<br>1316 (6)	
γ_{CH2}	**1343**<br>1319 (0.1)<br>1327 (2)<br>1345 (1)<br>1346 (2)<br>1352 (1)<br>1362 (0.4)	**1310**<br>1306 (3)<br>1311 (2)<br>1322 (1)<br>1330 (0.2)<br>1332 (0.3)<br>1349 (1)<br>1359 (0.5)	**1337**<br>1332 (4)<br>1338 (7)<br>1347 (1)	**S** 1368 (35)
ρ_{NH2}, γ_{CH2}, γ_{CH3}		**1398** (10)	**1374**<br>1365 (12)<br>1378 (12)<br>1381 (4)	**S** 1368 (35)
β_{CH2}, β_{CH3}	**1460**<br>1436 (5)<br>1440 (2)<br>1453 (13)<br>1461 (12)<br>1465 (12)<br>1487 (2)	**1443**<br>1432 (1)<br>1440 (15)<br>1448 (18)<br>**1470**<br>1467 (4)<br>1470 (18)<br>1474 (1)	**1441**<br>1421 (6)<br>1430 (14)<br>1431 (0.1)<br>1442 (21)<br>1444 (13)<br>1448 (4)<br>1468 (5)	**P** 1458 (25)
ν_{CN}			**1502** (95)	**T** 1551 (7)
ν_{CN}		**1545** (7)		
β_{NH2}	**1597** (54)			**Q$_1$** 1599 (6)
β_{NH2}			**1642** (200)	**Q$_2$** 1664 (13)
β_{NH2}		**1672** (187)		**Q$_3$** 1717 (7)
ν_{CH}		**2819** (8)		**A** 2842 (4)
ν_{CH}	**2844** (72)			**B** 2864 (11)
ν_{CH2}, ν_{CH3}			**2876**<br>2858 (0.4)<br>2871 (2)<br>2877 (17)	**B** 2864 (11)
ν_{CH2}, ν_{CH3}	**2922**<br>2908 (7)<br>2916 (11)<br>2922 (15)<br>2923 (21)<br>2925 (12)	**2904**<br>2898 (19)<br>2905 (30)<br>**2926**<br>2918 (0.8)<br>2924 (8)<br>2929 (10)	2899 (0.2)<br>2906 (8)<br>**2926**<br>2925 (11)<br>2935 (5)	**C** 2920 (6)
ν_{CHn}	**2960**<br>2948 (14)<br>2950 (15)<br>2951 (6)	**2972**<br>2955 (1)<br>2956 (12)<br>2970 (21)	**2961**<br>2951 (8)<br>2954 (13)<br>2957 (15)	**D** 2947 (25)<br>**E** 2959 (6)<br>**F** 2978 (4)

	2957 (7)<br>2962 (7)<br>2963 (11)<br>2967 (4)<br>2971 (28)	2971 (8)<br>2975 (10)<br>2975 (8)<br>2984 (6)	2963 (9)<br>2963 (19)<br>2965 (15)<br>2970 (9)<br>2983 (13)	
ν_{CH2}	**3026** (6)	3020 (6)		**G** 3008 (6)
ν_{CH2}		3120 (4)		
$2\beta_{NH2}$	3194[b]<br>3150 (12)[c]			**H** 3151 (9)
$2\beta_{NH2}$ [e]			3284[b]<br>3254 (0.7)[c]	**I** 3267 (8)
$2\beta_{NH2}$ [e]		3344[b]<br>3375 (0.3) [c]		
ν_{NH}^{s}	**3319** (241)			**J₁** 3321 (16)
ν_{NH}^{s}		**3324** (164)		**J₂** 3345 (8)
ν_{NH}^{s}			**3356** (176)	**J₃** 3381 (17)
ν_{NH}^{a}	**3435** (96)			**K₁** 3425 (9)
ν_{NH}^{a}		**3428** (120)		**K₂** 3451 (7)
ν_{NH}^{a}			**3464** (86)	**K₃** 3477 (11)

[a] IR intensities in km mol^{-1} are given in parentheses. [b] Harmonic value. [c] Result of anharmonic calculation.

[d] Stretching (ν), bending (β), torsing (τ), wagging (γ), rocking (ρ). [e] coupled β_{NH2}/ν_{CN} modes.

Table S4. Computed vibrational frequencies (in cm^{-1}, B3LYP-D3/cc-pVTZ) of Ama^+(**I-III**)-N_2(**I**) compared to experimental values of Ama^+N_2 (Figure 6).[a] In bold are the folded calculated peaks. The experimental values are given with peak width (fwhm in parenthesis) and are assigned to the most dominant vibrations.

Mode [b]	Ama^+(**I**)-N_2(**I**)	Ama^+(**II**)-N_2(**I**)	Ama^+(**III**)-N_2(**I**)	Ama^+-N_2 Exp.
ν_{CH}		**2818** (8)		**A** 2841 (7)
ν_{CH}	**2852** (70)			**B** 2876 (16)
ν_{CH2}, ν_{CH3}			**2877**<br>2858 (0.8)<br>2872 (2)<br>2877 (19)	**B** 2876 (16)
ν_{CH2}, ν_{CH3}	**2921**<br>2910 (6)<br>2915 (15)<br>2922 (15)<br>2923 (20)<br>2925 (15)	**2902**<br>2898 (12)<br>2902 (40)<br>**2925**<br>2917 (0.3)<br>2923 (9)<br>2927 (10)	2900 (0.1)<br>2907 (9)<br>**2926**<br>2924 (11)<br>2929 (7)	**C** 2923 (11)
ν_{CHn}	**2957**<br>2948 (21)<br>2951 (7)<br>2951 (11)<br>2954 (7)<br>2960 (8)<br>2962 (11)<br>2966 (4)<br>2970 (29)	**2972**<br>2954 (3)<br>2955 (10)<br>2969 (21)<br>2970 (9)<br>2974 (10)<br>2975 (9)<br>2982 (8)	**2962**<br>2950 (8)<br>2952 (14)<br>2957 (12)<br>2962 (15)<br>2963 (10)<br>2964 (26)<br>2966 (5)<br>2975 (20)	**D** 2944 (11)<br>**E** 2966 (7)<br>**F** 2984 (5)
ν_{CH2}	3021 (8)	3020 (6)		**G** 3020 (4)
ν_{CH2}		3120 (4)		**U** 3103 (5)
$2\beta_{NH2}$	3207			**H** 3172 (14)
$2\beta_{NH2}$ [c]			3300	**I** 3282 (9)
$2\beta_{NH2}$ [c]		3322		
ν_{NH}^{s}	**3276** (567)			**J_1** 3307 (15)
ν_{NH}^{s}		**3281** (413)		**J_2** 3316 (3)
ν_{NH}^{s}			**3329** (363)	**J_3** 3372 (18)
ν_{NH}^{a}	**3411** (143)			**K_1** 3417 (12)
ν_{NH}^{a}		**3408** (142)		**K_2** 3427 (2)
ν_{NH}^{a}			**3448** (133)	**K_3** 3467 (13)

[a] IR intensities in km/mol are given in parentheses. [b] Stretching (ν), bending (β). [c] coupled β_{NH2}/ν_{CN} modes.

Table S5. Computed vibrational frequencies (in cm^{-1}, B3LYP-D3/cc-pVTZ) of Ama^+(**I-III**)-H_2O(**I**) and H_2O compared to experimental values of Ama^+H_2O (Figure 7).[a] In bold are the convoluted calculated peaks. The experimental values are given with peak width (fwhm in parenthesis) and are assigned to the most dominant vibrations.

Mode [d]	Ama^+(**I**)-H_2O (**I**)	Ama^+(**II**)-H_2O (**I**)	Ama^+(**III**)-H_2O (**I**)	Ama^+- H_2O Exp.	H_2O
ν_{CH}		**2814** (9)			
ν_{CH}	**2869** (59)			**B** 2865 (10)	
ν_{CH2}, ν_{CH3}			**2869**<br>2856 (3)<br>2864 (4)<br>2869 (27)	**B** 2865 (10)	
ν_{CH2}, ν_{CH3}	**2919**<br>2907 (11)<br>2918 (12)<br>2919 (14)<br>2920 (22)<br>2923 (14)	**2900**<br>2897 (11)<br>2901 (41)<br>**2925**<br>2916 (1)<br>2922 (7)<br>2927 (12)	**2906**<br>2899 (0.3)<br>2906 (10)<br>**2924**<br>2923 (12)<br>2926 (8)	**D** 2937 (35)	
ν_{CHn}	**2957**<br>2945 (23)<br>2948 (14)<br>2950 (14)<br>2954 (7)<br>2958 (5)<br>2962 (7)<br>2963 (12)<br>2967 (36)	**2969**<br>2952 (4)<br>2953 (12)<br>2967 (27)<br>2968 (10)<br>2971 (10)<br>2973 (14)<br>2992 (3)	**2958**<br>2945 (10)<br>2949 (18)<br>2956 (11)<br>2958 (29)<br>2960 (14)<br>2963 (12)<br>2972 (19)<br>2979 (5)	**D** 2937 (35)	
ν_{CH2}	3012 (8)	3019 (7)			
ν_{CH2}		3118 (5)			
$2\beta_{NH2}$	3176 [b]<br>3187 (88)[c]			**H** 3187 (10)	
$2\beta_{OH2}$	3238 [b]<br>3211 (58)[c]	3204 [b]<br>3204 (0.1)[c]	3203	**V** 3213 (6)	
$2\beta_{NH2}$ [e]			3325	**I** 3318 (10)	
$2\beta_{NH2}$ [e]		3374			
ν_{NH} [b]	**3023** (1265)			**L₁** 2990 (19)	
ν_{NH} [b]		**3033** (1023)		**L₂** 3021 (9)	
ν_{NH} [b]			**3123** (951)	**L₃** 3147 (16)	
ν_{NH} [f]	**3378** (95)			**M₁** 3368 (27)	
ν_{NH} [f]		**3389** (86)		**M₂** 3387 (6)	
ν_{NH} [f]			**3420** (90)	**M₃** 3433 (5)	
ν_{OH} [s]	**3597** (51)	**3596** (48)	**3599** (44)	**N** 3627 (31)	3605 (3)
ν_{OH} [a]	**3682** (110)	**3682** (114)	**3684** (101)	**O** 3717 (28)	3700 (41)

[a] IR intensities in km/mol are given in parentheses. The computational data for isomers **II** and **III** are available in Table S2. [b] Harmonic value. [c] Result of anharmonic calculation. [d] Stretching (ν), bending (β), torsing (τ), wagging (γ), rocking (ρ). [e] coupled β_{NH2}/ν_{CN} modes.

Table S6. Computed vibrational frequencies (in cm^{-1}, B3LYP-D3/cc-pVTZ) of $\nu_{NH}^{s/a}$ and $\nu_{NH}^{b/f}$ modes of Ama$^+$(**I-III**) and Ama$^+$(**I-III**)-L (L=Ar_{1-3}, N_2, H_2O) (Figure 8). IR intensities in km/mol are given in parentheses.

Isomer	$\nu_{NH}^{s}/\nu_{NH}^{b}$	$\nu_{NH}^{a}/\nu_{NH}^{f}$
Ama$^+$(**I**)	3322 (223)	3438 (68)
Ama$^+$(**II**)	3329 (131)	3433 (76)
Ama$^+$(**III**)	3358 (173)	3433 (63)
Ama$^+$(**I**)-Ar(**I**)	3319 (241)	3435 (96)
Ama$^+$(**II**)-Ar(**I**)	3324 (164)	3428 (120)
Ama$^+$(**III**)-Ar(**I**)	3356 (176)	3464 (86)
Ama$^+$(**I**)-Ar_2(**I**)	3316 (262)	3432 (135)
Ama$^+$(**II**)-Ar_2(**I**)	3320 (176)	3423 (175)
Ama$^+$(**III**)-Ar_2(**I**)	3355 (178)	3464 (116)
Ama$^+$(**I**)-Ar_3(**I**)	3318 (245)	3435 (131)
Ama$^+$(**II**)-Ar_3(**I**)	3320 (176)	3423 (183)
Ama$^+$(**II**)-Ar_3(**I**)	3358 (172)	3465 (104)
Ama$^+$(**I**)-N_2(**I**)	3276 (567)	3411 (143)
Ama$^+$(**II**)-N_2(**I**)	3281 (413)	3408 (142)
Ama$^+$(**III**)-N_2(**I**)	3329 (363)	3448 (133)
Ama$^+$(**I**)-H_2O(**I**)	3023 (1265)	3378 (95)
Ama$^+$(**I**)-H_2O(**I**)	3033 (1023)	3389 (86)
Ama$^+$(**I**)-H_2O(**I**)	3123 (951)	3420 (90)

Table S7. Various energies of the Ama^+(**I-III**)-Ar, Ama^+(**I-III**)-N_2 and Ama^+(**I-III**)-H_2O isomers calculated at the B3LYP-D3/cc-pVTZ level. Energies are given in kJ mol^{-1}.

	E_0	E_e	G	D_0
Ama^+(**I**)	45.88	40.59	51.86	
Ama^+(**II**)	87.38	88.04	85.92	
Ama^+(**III**)	0	0	0	
Ama^+TS(**I-II**)	99.95	100.13	101.38	
Ama^+TS(**II-III**)	169.01	178.67	172.22	
Ama^+(**I**)-Ar(**I**)	46.30	40.54	50.43	8.36
Ama^+(**II**)-Ar(**I**)	87.27	87.92	84.28	8.90
Ama^+(**III**)-Ar(**I**)	0	0	0	8.79
Ama^+TS(**I-II**)-Ar	99.16	99.95	97.95	
Ama^+TS(**II-III**)-Ar	169.14	179.85	168.13	
Ama^+(**I**)-N_2(**I**)	46.33	39.21	53.86	13.41
Ama^+(**II**)-N_2(**I**)	85.59	85.66	85.38	15.66
Ama^+(**III**)-N_2(**I**)	0	0	0	13.87
Ama^+TS(**I-II**)-N_2	97.83	97.75	99.74	
Ama^+TS(**II-III**)-N_2	170.52	180.12	173.30	
Ama^+(**I**)-H_2O (**I**)	43.21	36.59	49.24	61.44
Ama^+(**II**)-H_2O (**I**)	79.75	80.47	79.06	66.39
Ama^+(**III**)-H_2O (**I**)	0	0	0	58.77
Ama^+TS(**I-II**)-H_2O	93.12	93.40	95.08	
Ama^+TS(**II-III**)-H_2O	212.53	224.66	211.94	

Table S8. Various energies of the Ama$^+$(**I-III**)-Ar_1(**I-VIII**) isomers calculated at the B3LYP-D3/cc-pVTZ level. Energies are given in kJ mol^{-1}.

	E_0	E_e	G	D_0	E_0^{total}
Ama$^+$(**I**)-Ar(**I**)	0	0	0	8.36	46.31
Ama$^+$(**I**)-Ar(**II**)	0.67	0.75	-0.56	7.69	46.98
Ama$^+$(**I**)-Ar(**III**)	1.17	1.06	2.71	7.19	47.47
Ama$^+$(**I**)-Ar(**IV**)	2.08	2.16	2.00	6.28	48.39
Ama$^+$(**I**)-Ar(**V**)	2.21	3.31	-1.80	6.15	48.51
Ama$^+$(**I**)-Ar(**VI**)	2.72	3.23	2.32	5.65	49.02
Ama$^+$(**I**)-Ar(**VII**)	2.85	3.62	1.90	5.51	49.16
Ama$^+$(**I**)-Ar(**VIII**)	2.91	3.69	-0.13	5.45	49.22
Ama$^+$(**II**)-Ar(**I**)	0	0	0	8.90	87.27
Ama$^+$(**II**)-Ar(**II**)	0.94	1.38	-0.48	7.97	88.21
Ama$^+$(**II**)-Ar(**III**)	1.81	2.32	0.71	7.08	89.09
Ama$^+$(**II**)-Ar(**IV**)	1.91	2.29	1.73	6.99	89.19
Ama$^+$(**II**)-Ar(**V**)	2.08	2.29	1.87	6.82	89.35
Ama$^+$(**II**)-Ar(**VI**)	2.26	2.10	2.97	6.64	89.53
Ama$^+$(**II**)-Ar(**VII**)	4.84	5.20	3.78	4.06	92.11
Ama$^+$(**III**)-Ar(**I**)	0	0	0	8.79	0
Ama$^+$(**III**)-Ar(**II**)	1.57	2.44	-3.27	7.22	1.57
Ama$^+$(**III**)-Ar(**III**)	1.90	2.49	0.01	6.89	1.90
Ama$^+$(**III**)-Ar(**IV**)	2.20	2.16	0.74	6.59	2.20
Ama$^+$(**III**)-Ar(**V**)	2.47	2.79	-0.52	6.33	2.47
Ama$^+$(**III**)-Ar(**VI**)	2.75	3.00	1.32	6.04	2.75
Ama$^+$(**III**)-Ar(**VII**)	2.92	2.88	1.46	5.88	2.92

Table S9. Various energies of the Ama^{+}(**I-III**)-Ar_2(**I-VIII**) isomers calculated at the B3LYP-D3/cc-pVTZ level. Energies are given in kJ mol^{-1}.

	E_0	E_e	G	D_0	E_0^{total}
Ama^{+}(**I**)-Ar_2(**I**)	0	0	0	16.91	47.20
Ama^{+}(**I**)-Ar_2**II**)	0.83	1.25	0.60	16.08	48.03
Ama^{+}(**I**)-Ar_2(**III**)	2.07	1.95	2.60	14.83	49.27
Ama^{+}(**I**)-Ar_2(**IV**)	2.72	2.90	2.33	14.19	49.91
Ama^{+}(**I**)-Ar_2(**V**)	2.91	2.72	4.95	14.00	50.10
Ama^{+}(**I**)-Ar_2(**VI**)	3.65	3.54	7.19	13.26	50.84
Ama^{+}(**I**)-Ar_2(**VII**)	3.78	3.97	4.25	13.12	50.98
Ama^{+}(**I**)-Ar_2(**VIII**)	5.68	5.53	7.61	11.23	52.87
Ama^{+}(**II**)-Ar_2(**I**)	0	0	0	17.97	87.64
Ama^{+}(**II**)-Ar_2(**II**)	0.07	0.50	0.19	17.90	87.71
Ama^{+}(**II**)-Ar_2(**III**)	1.85	2.11	1.67	16.12	89.49
Ama^{+}(**II**)-Ar_2(**IV**)	1.88	2.17	2.04	16.09	89.52
Ama^{+}(**II**)-Ar_2(**V**)	2.15	2.05	3.53	15.81	89.79
Ama^{+}(**II**)-Ar_2(**VI**)	2.24	2.07	3.21	15.72	85.88
Ama^{+}(**II**)-Ar_2(**VII**)	2.51	2.69	2.39	15.46	90.15
Ama^{+}(**II**)-Ar_2(**VIII**)	3.47	4.31	2.46	14.50	91.11
Ama^{+}(**II**)-Ar_2(**IX**)	3.69	4.27	1.93	14.27	91.33
Ama^{+}(**III**)-Ar_2 (**I**)	0	0	0	18.23	0
Ama^{+}(**III**)-Ar_2(**II**)	2.02	2.48	-0.66	16.20	2.02
Ama^{+}(**III**)-Ar_2(**III**)	2.62	2.34	2.14	15.60	2.62
Ama^{+}(**III**)-Ar_2(**IV**)	2.64	2.64	2.13	15.58	2.64
Ama^{+}(**III**)-Ar_2(**V**)	2.70	1.99	1.45	15.53	2.70
Ama^{+}(**III**)-Ar_2(**VI**)	2.96	2.97	1.55	15.26	2.96
Ama^{+}(**III**)-Ar_2(**VII**)	2.97	3.04	1.55	15.26	2.97
Ama^{+}(**III**)-Ar_2(**VIII**)	3.13	3.07	1.82	15.10	3.13
Ama^{+}(**III**)-Ar_2(**IX**)	3.50	3.68	-0.27	14.72	3.50
Ama^{+}(**III**)-Ar_2(**X**)	5.73	4.69	6.04	12.50	5.73

Table S10. Various energies of the Ama^+(**I-III**)-Ar_3(**I-VIII**) isomers calculated at the B3LYP-D3/cc-pVTZ level. Energies are given in kJ mol^{-1}.

	E_0	E_e	G	D_0	E_0^{total}
Ama^+(**I**)-Ar_3(**I**)	0	0	0	24.68	46.03
Ama^+(**I**)-Ar_3(**II**)	1.24	0.81	1.61	23.44	47.27
Ama^+(**I**)-Ar_3(**III**)	1.37	0.76	1.29	23.31	47.41
Ama^+(**I**)-Ar_3(**IV**)	2.04	2.06	-3.05	22.64	48.08
Ama^+(**I**)-Ar_3(**V**)	2.14	2.03	1.38	22.54	48.18
Ama^+(**I**)-Ar_3(**VI**)	2.14	1.81	3.57	22.53	48.18
Ama^+(**I**)-Ar_3(**VII**)	2.19	1.84	-2.08	22.49	48.23
Ama^+(**I**)-Ar_3(**VIII**)	2.30	1.34	3.03	22.37	48.34
Ama^+(**I**)-Ar_3(**IX**)	2.88	2.85	1.63	21.80	48.91
Ama^+(**I**)-Ar_3(**X**)	3.36	3.47	2.49	21.31	49.40
Ama^+(**I**)-Ar_3(**XI**)	4.21	4.62	2.81	20.47	50.25
Ama^+(**I**)-Ar_3(**XII**)	4.22	4.14	0.75	20.45	50.26
Ama^+(**I**)-Ar_3(**XIII**)	4.93	4.31	6.25	19.75	50.96
Ama^+(**II**)-Ar_3(**I**)	0	0	0	27.38	84.83
Ama^+(**II**)-Ar_3(**II**)	1.92	2.07	-0.83	25.46	86.76
Ama^+(**II**)-Ar_3(**III**)	2.39	2.00	0.17	24.99	87.23
Ama^+(**II**)-Ar_3(**IV**)	2.57	1.20	1.19	24.81	87.41
Ama^+(**II**)-Ar_3(**V**)	2.59	2.46	2.84	24.79	87.42
Ama^+(**II**)-Ar_3(**VI**)	2.64	2.44	2.19	24.74	87.48
Ama^+(**II**)-Ar_3(**VII**)	3.09	2.30	3.39	24.29	87.92
Ama^+(**II**)-Ar_3(**VIII**)	3.76	3.54	3.05	23.61	88.60
Ama^+(**II**)-Ar_3(**IX**)	4.08	3.80	4.20	23.30	88.91
Ama^+(**II**)-Ar_3(**X**)	4.47	4.14	3.63	22.91	89.30
Ama^+(**II**)-Ar_3(**XI**)	4.69	4.69	2.79	22.69	89.23
Ama^+(**II**)-Ar_3(**XII**)	4.84	4.73	1.51	22.53	89.69
Ama^+(**III**)-Ar_3(**I**)	0	0	0	24.83	0
Ama^+(**III**)-Ar_3(**II**)	0.09	0.93	-4.15	24.75	0.09
Ama^+(**III**)-Ar_3(**III**)	0.29	0.93	-3.78	24.55	0.29
Ama^+(**III**)-Ar_3(**IV**)	0.30	0.75	-0.92	24.53	0.30
Ama^+(**III**)-Ar_3(**V**)	0.31	1.44	-5.34	24.52	0.31
Ama^+(**III**)-Ar_3(**VI**)	1.23	1.40	-1.63	23.61	1.23
Ama^+(**III**)-Ar_3(**VII**)	2.52	3.38	-2.15	22.32	2.52
Ama^+(**III**)-Ar_3(**VIII**)	3.10	3.71	-1.15	21.73	3.10
Ama^+(**III**)-Ar_3(**IX**)	3.29	4.23	-2.49	21.55	3.29
Ama^+(**III**)-Ar_3(**X**)	3.50	3.83	-0.17	21.34	3.50
Ama^+(**III**)-Ar_3(**XI**)	3.51	3.85	-0.18	21.32	3.51

Ama^+(**III**)-Ar_3(**XII**)	3.54	4.24	-2.29	21.30	3.53
Ama^+(**III**)-Ar_3(**XIII**)	3.99	5.24	-2.76	20.85	3.99

Table S11. Various energies of the Ama^+(**I-III**)-N_2(**I-VII**) isomers calculated at the B3LYP-D3/cc-pVTZ level. Energies are given in kJ mol^{-1}.

	E_0	E_e	G	D_0	E_0^{total}
Ama^+(**I**)-N_2(**I**)	0	0	0	13.41	46.33
Ama^+(**I**)-N_2(**II**)	4.12	5.49	3.44	9.29	50.45
Ama^+(**I**)-N_2(**III**)	6.16	8.02	4.20	7.25	52.50
Ama^+(**I**)-N_2(**IV**)	6.84	9.17	1.05	6.57	53.17
Ama^+(**I**)-N_2(**V**)	6.86	8.97	3.00	6.55	53.20
Ama^+(**II**)-N_2(**I**)	0	0	0	15.66	85.59
Ama^+(**II**)-N_2(**II**)	5.55	7.04	2.41	10.11	91.14
Ama^+(**II**)-N_2(**III**)	6.27	7.14	4.36	9.39	91.86
Ama^+(**II**)-N_2(**IV**)	6.83	8.17	3.87	8.83	92.42
Ama^+(**II**)-N_2(**V**)	7.24	8.49	4.50	8.42	92.82
Ama^+(**III**)-N_2(**I**)	0	0	0	13.87	0
Ama^+(**III**)-N_2(**II**)	4.08	5.35	1.67	9.79	4.08
Ama^+(**III**)-N_2(**III**)	5.55	6.42	3.34	8.31	5.55
Ama^+(**III**)-N_2(**IV**)	5.96	6.33	4.48	7.91	5.96
Ama^+(**III**)-N_2(**V**)	5.98	6.73	2.32	7.89	5.98
Ama^+(**III**)-N_2(**VI**)	6.58	6.96	4.07	7.29	6.58
Ama^+(**III**)-N_2(**VII**)	6.90	7.42	4.32	6.97	6.90

Table S12. Various energies of the Ama^+(**I-III**)-H_2O(**I-IV**) isomers calculated at the B3LYP-D3/cc-pVTZ level. Energies are given in kJ mol^{-1}.

	E_0	E_e	G	D_0	E_0^{total}
Ama^+(**I**)-H_2O(**I**)	0	0	0	61.44	43.10
Ama^+(**I**)-H_2O(**II**)	27.82	31.00	25.25	33.61	70.92
Ama^+(**I**)-H_2O(**III**)	30.86	34.42	26.74	30.57	73.96
Ama^+(**II**)-H_2O(**I**)	0	0	0	66.39	79.64
Ama^+(**II**)-H_2O(**II**)	24.41	25.75	23.09	41.98	104.06
Ama^+(**II**)-H_2O(**III**)	25.88	27.80	23.78	40.51	105.52
Ama^+(**II**)-H_2O(**IV**)	34.72	37.30	32.39	31.67	114.36
Ama^+(**II**)-H_2O(**V**)	36.22	38.53	33.17	30.17	115.86
Ama^+(**III**)-H_2O(**I**)	0	0	0	58.66	0
Ama^+(**III**)-H_2O(**II**)	19.52	21.38	17.16	39.13	19.52
Ama^+(**III**)-H_2O(**III**)	26.61	28.01	26.74	32.05	26.61

Table S13. Various energies (in kJ mol^{-1}) of the Ada^+(**I-III**) isomers and transition states calculated at the B3LYP-D3/cc-pVTZ level.

	E_0	E_e	G
Ada^+(**I**)	5.37	10.96	12.43
Ada^+(**II**)	152.95	162.74	148.41
Ada^+(**III**)	0	0	0
Ada^+TS(**I-II**)	162.03	170.02	158.56
Ada^+TS(**II-III**)	190.77	202.91	188.63

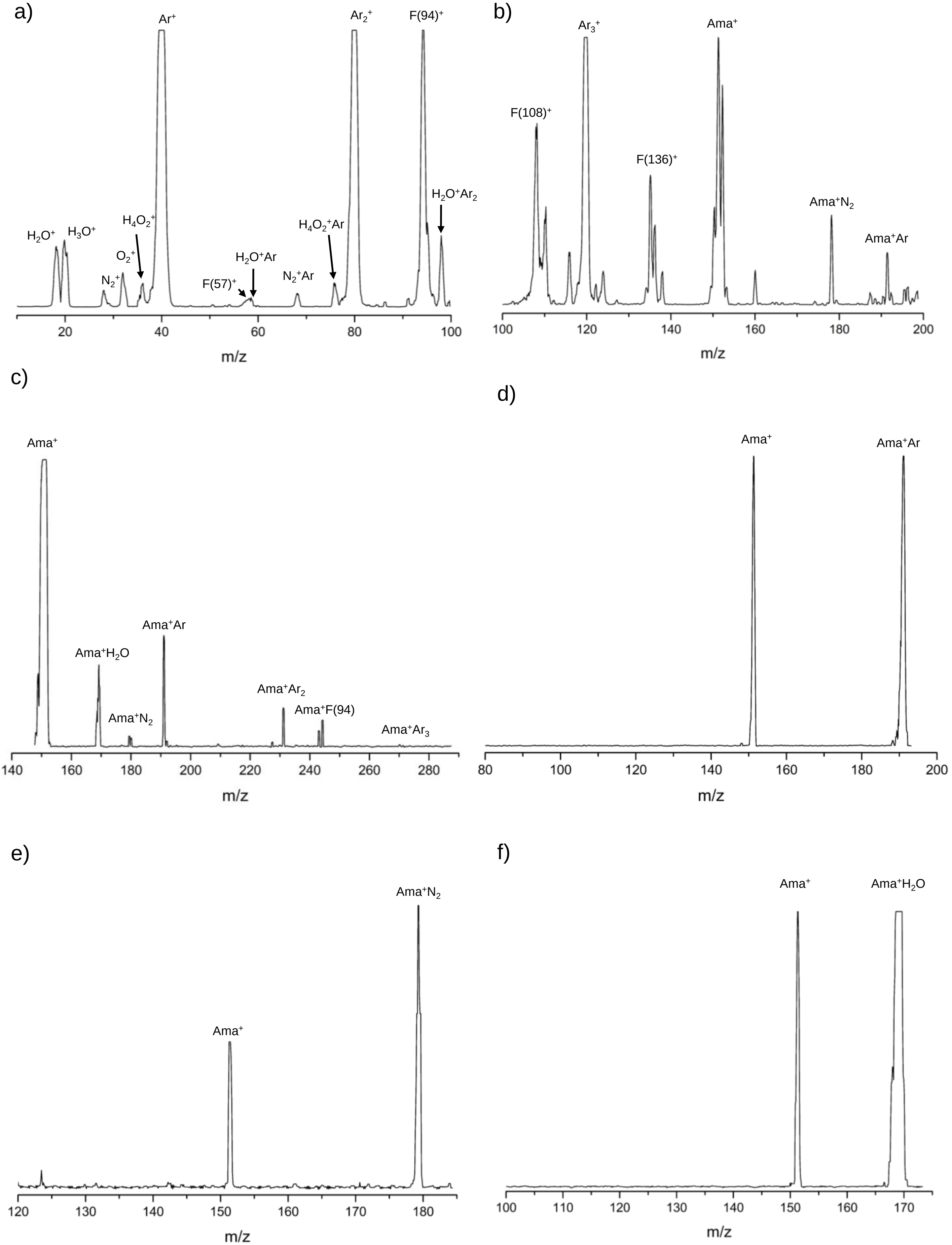

a)
H_2O^+
H_3O^+
N_2^+
O_2^+
$H_4O_2^+$
Ar^+
F(57)$^+$
H_2O^+Ar
N_2^+Ar
$H_4O_2^+Ar$
Ar_2^+
F(94)$^+$
$H_2O^+Ar_2$
20
40
60
80
100
m/z
b)
F(108)$^+$
Ar_3^+
F(136)$^+$
Ama$^+$
Ama$^+N_2$
Ama$^+$Ar
100
120
140
160
180
200
m/z
c)
Ama$^+$
Ama^+H_2O
Ama$^+N_2$
Ama$^+$Ar
Ama$^+Ar_2$
Ama$^+$F(94)
Ama$^+Ar_3$
140
160
180
200
220
240
260
280
m/z
d)
Ama$^+$
Ama$^+$Ar
80
100
120
140
160
180
200
m/z
e)
Ama$^+$
Ama$^+N_2$
120
130
140
150
160
170
180
m/z
f)
Ama$^+$
Ama^+H_2O
100
110
120
130
140
150
160
170
m/z

Figure S1

Figure S2

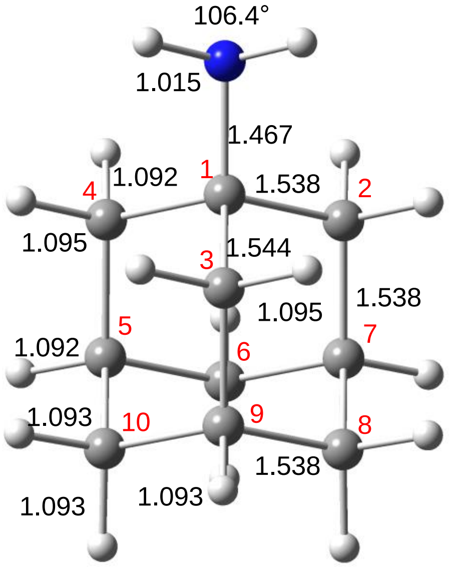

Ama (C_s)

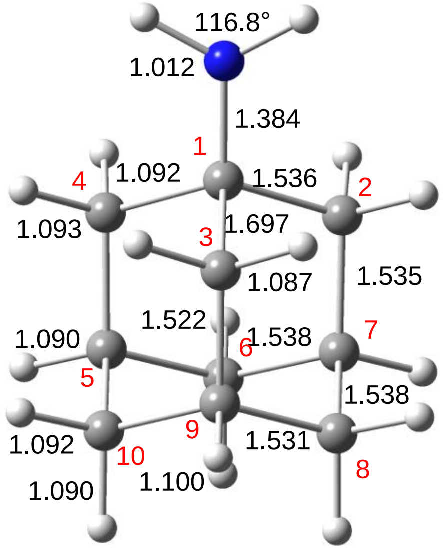

Ama$^+$(**I**) (C_s)

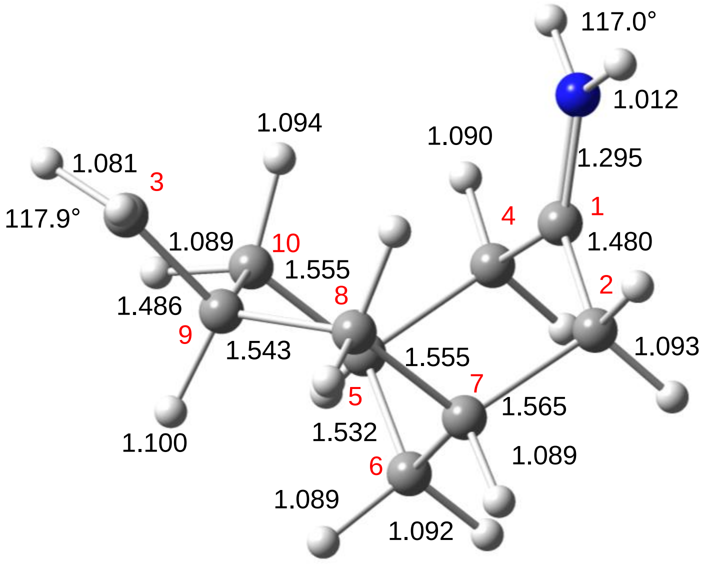

Ama$^+$(**II**) (C_s)

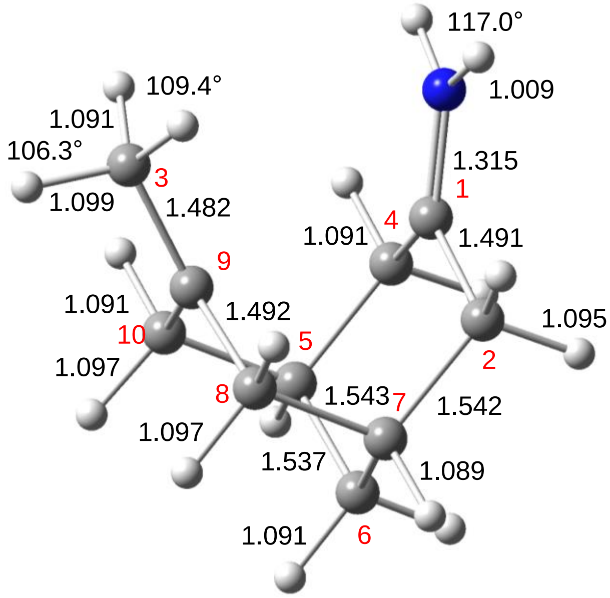

Ama$^+$(**III**) (C_s)

Figure S3

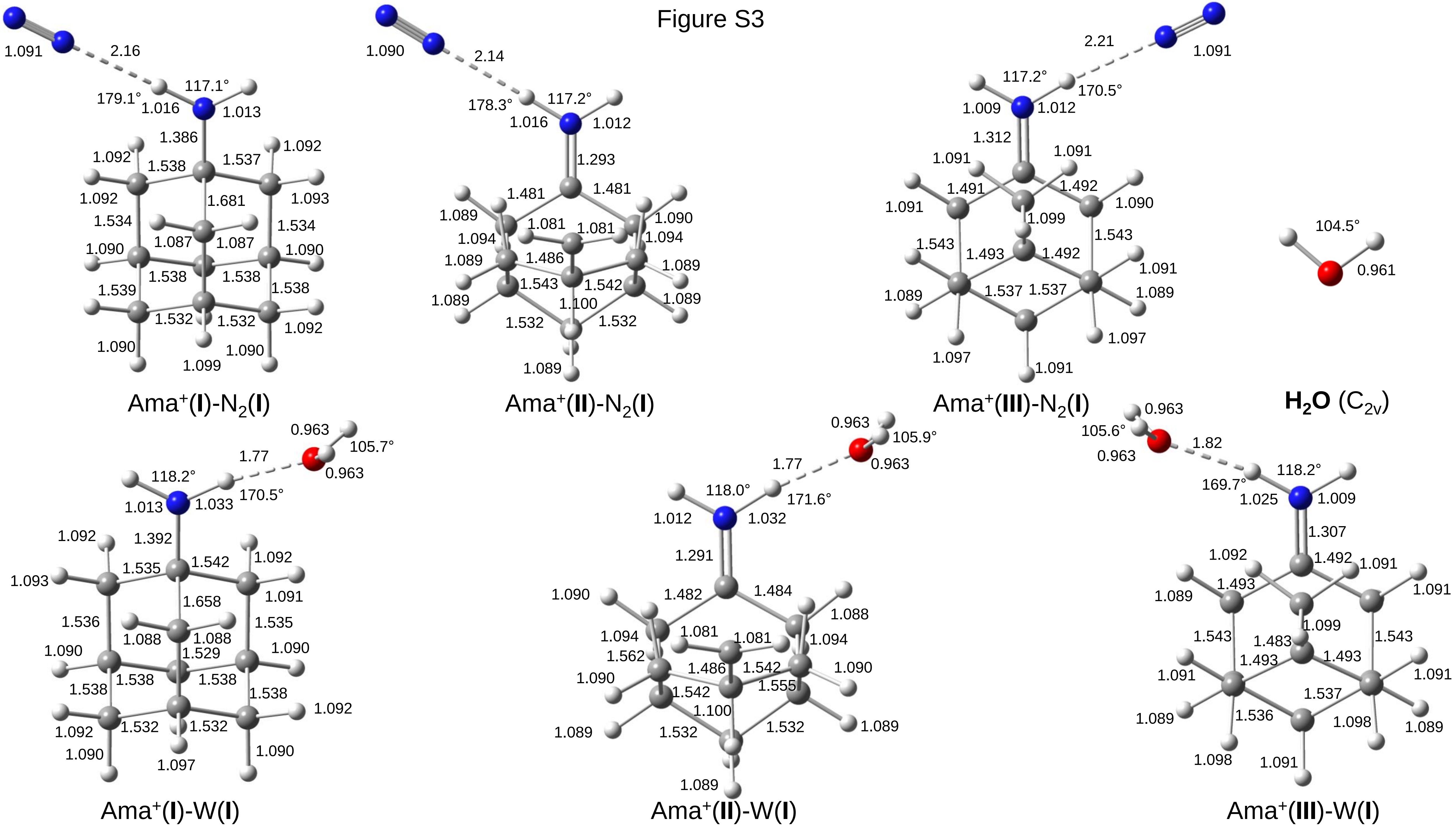

Ama⁺(I)-N₂(I)
Ama⁺(II)-N₂(I)
Ama⁺(III)-N₂(I)
H₂O (C2v)
Ama⁺(I)-W(I)
Ama⁺(II)-W(I)
Ama⁺(III)-W(I)

Figure S4

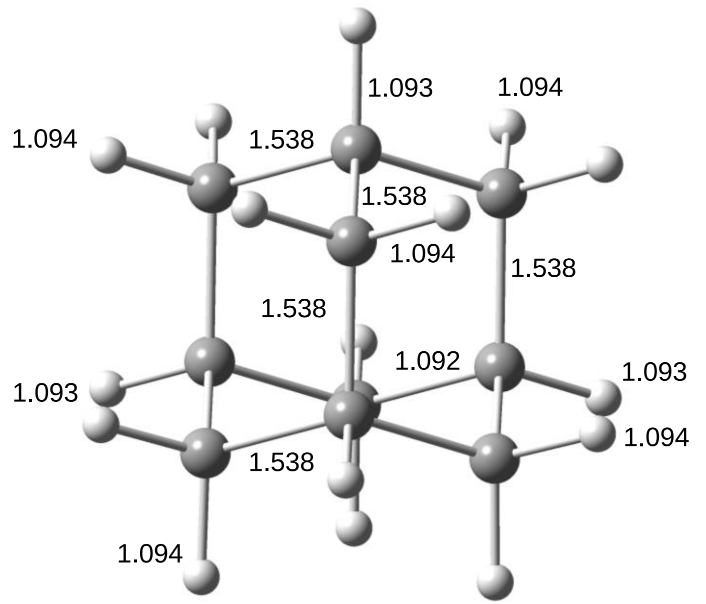

Ada (T_d)

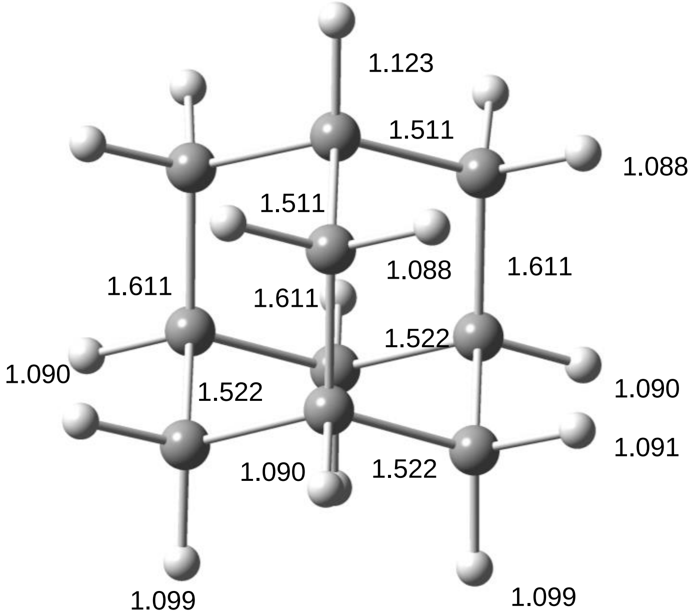

Ada$^+$(**I**) (C_{3v})

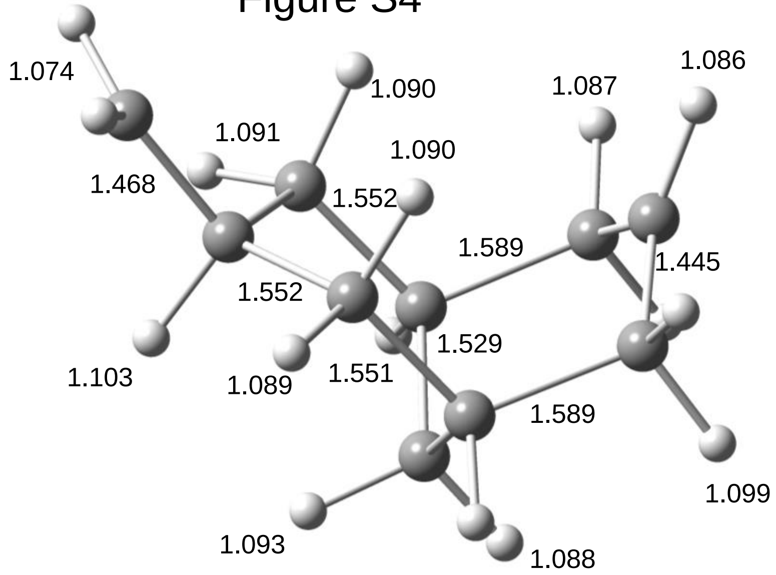

Ada$^+$(TS **I-II**) (C_s)

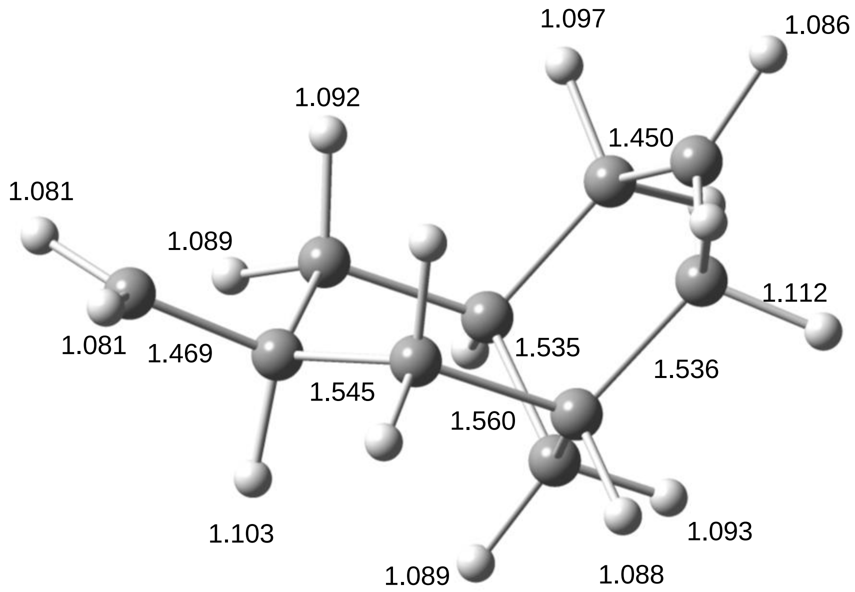

Ada$^+$(**II**) (C_s)

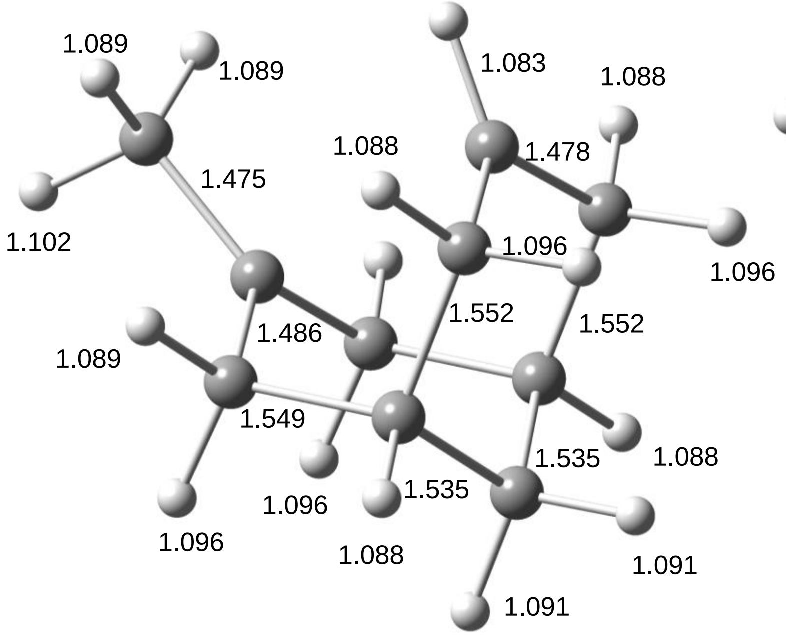

Ada$^+$(**III**) (C_s)

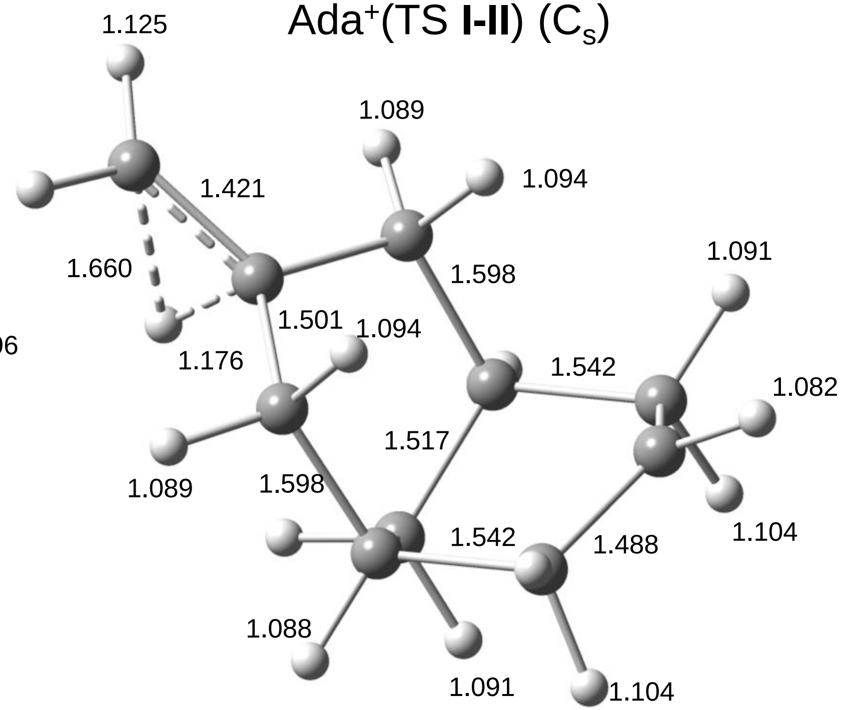

Ada$^+$(TS **II-III**) (C_s)

Figure S5

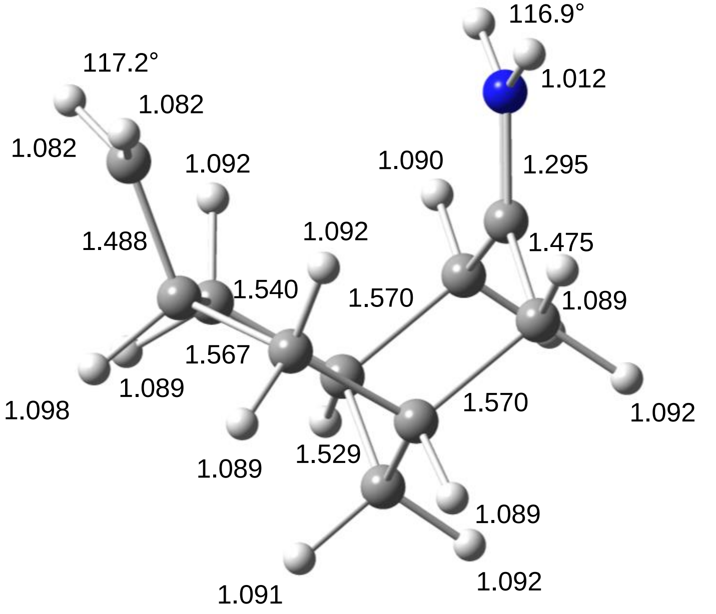

TS(I-II) (C_s)

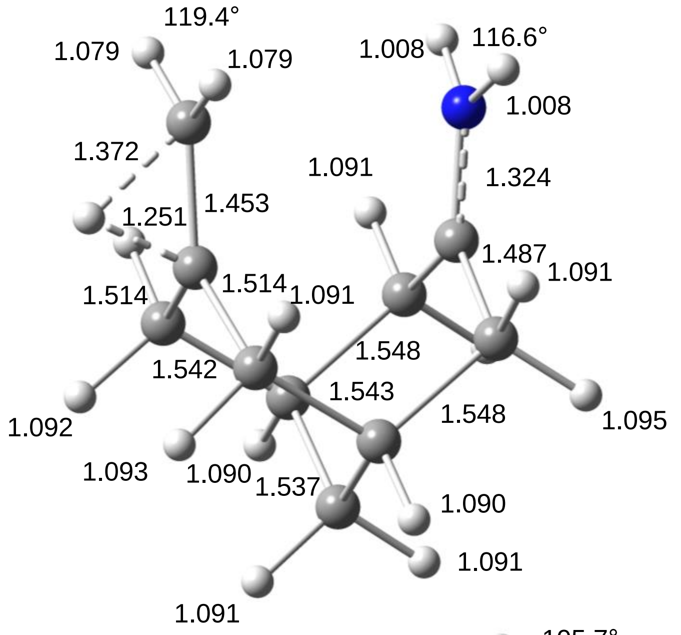

TS(II-III) (C_s)

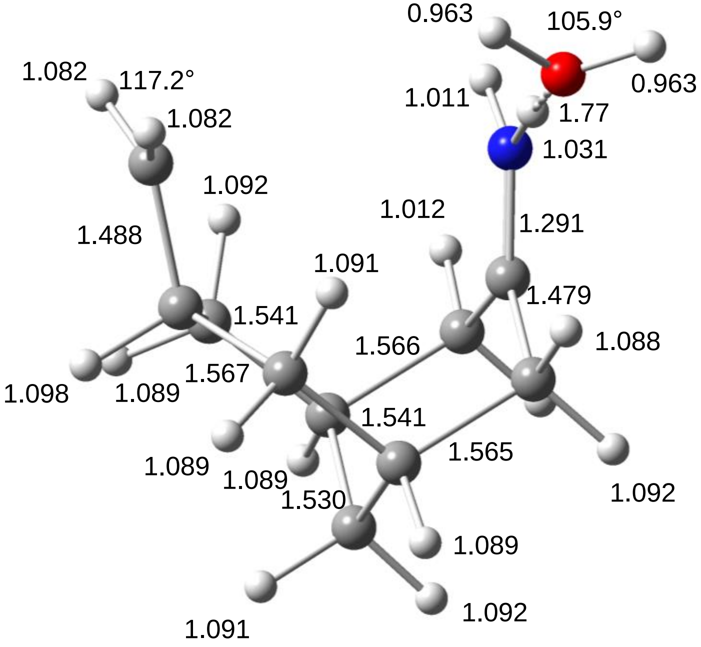

TS(I-II)-H_2O

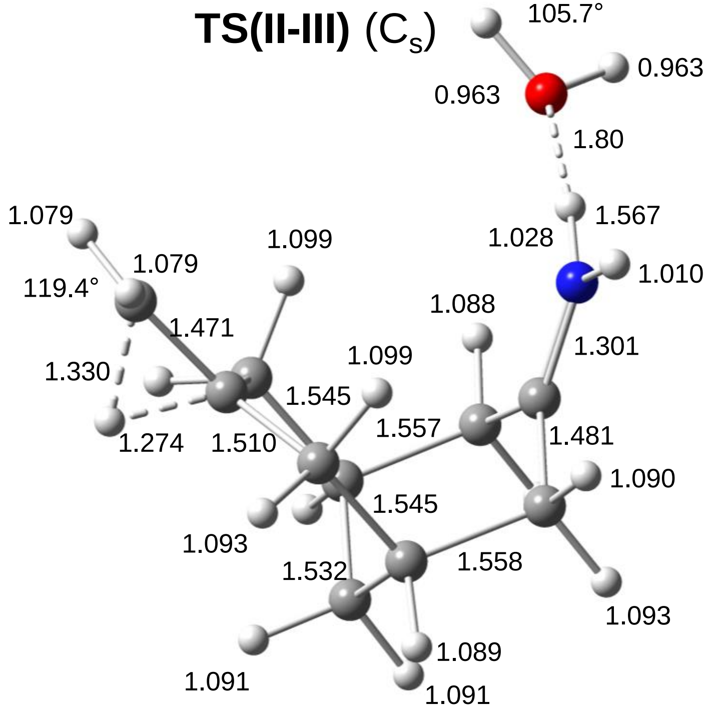

TS(II-III)-H_2O

Figure S6

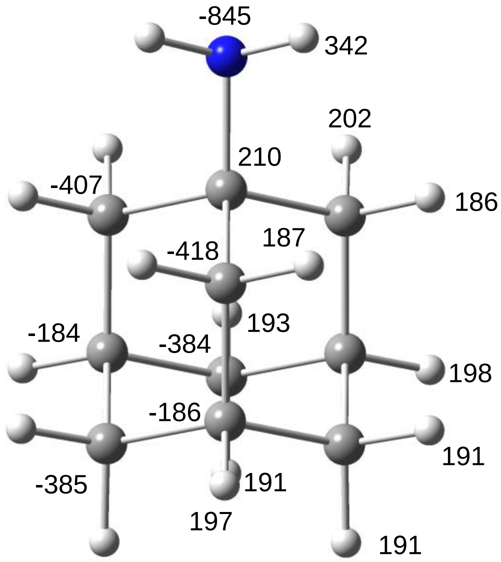

Ama (C_s)

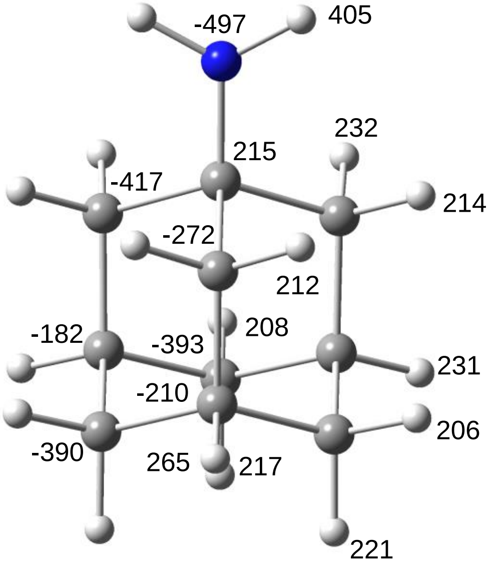

Ama$^+$ (C_s)

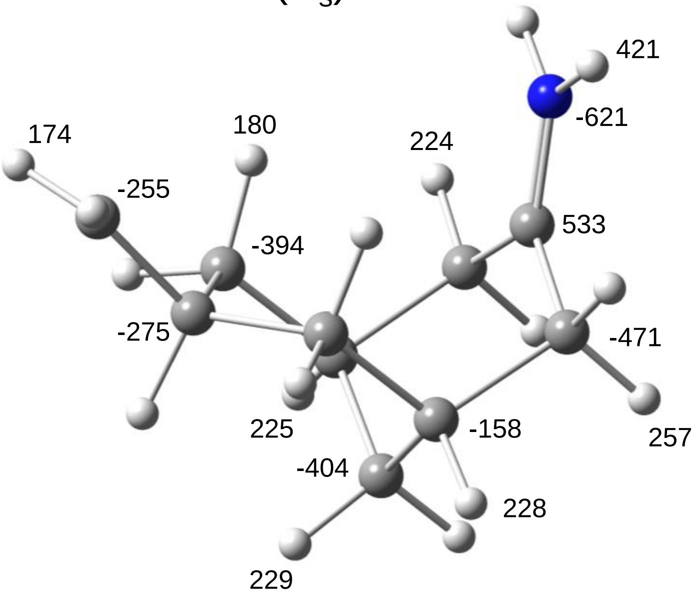

Ama$^+$(**II**) (C_s)

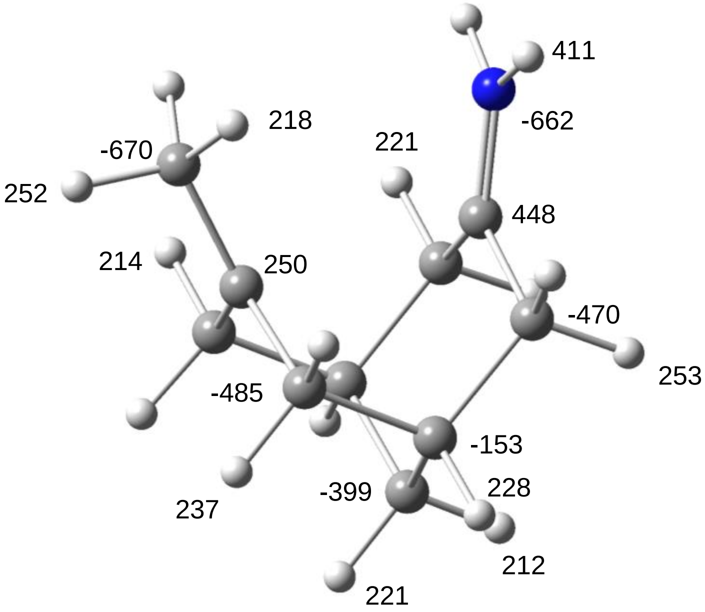

Ama$^+$(**III**) (C_s)

Figure S7

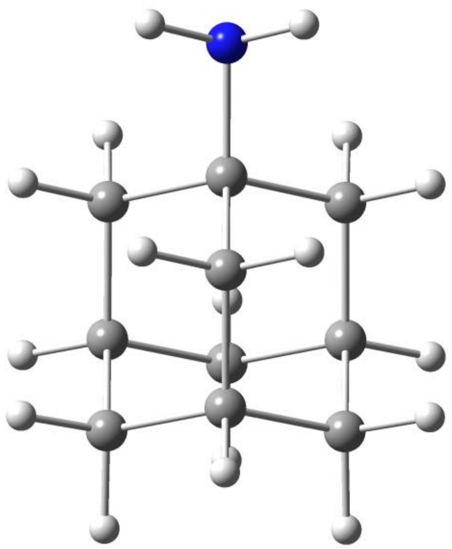

Ama (C_s)

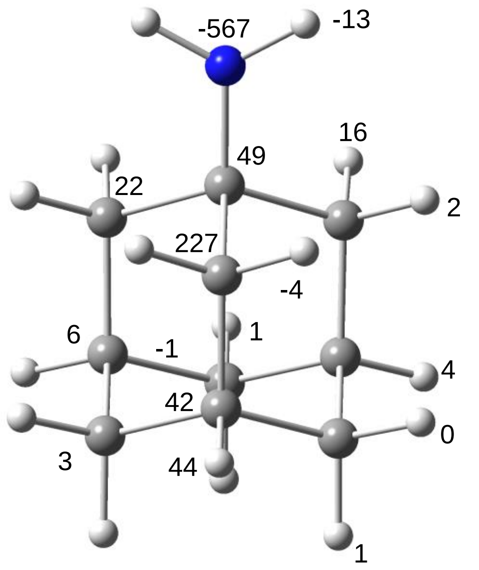

Ama$^+$ (C_s)

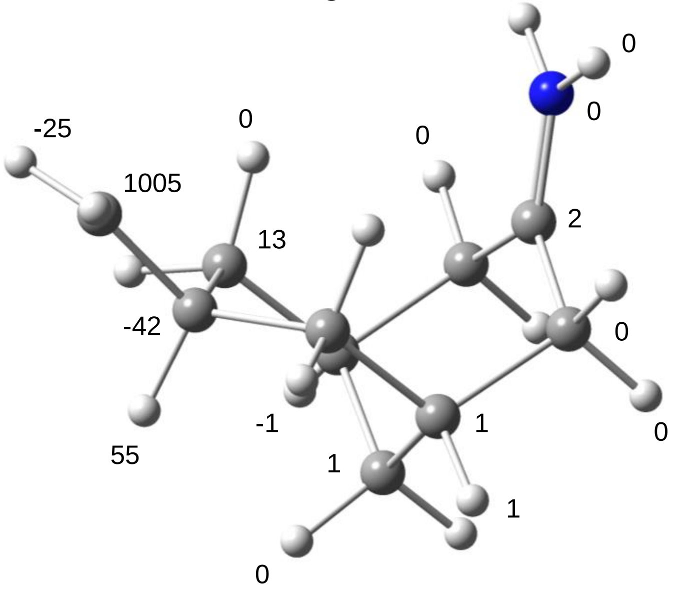

Ama$^+$(**II**) (C_s)

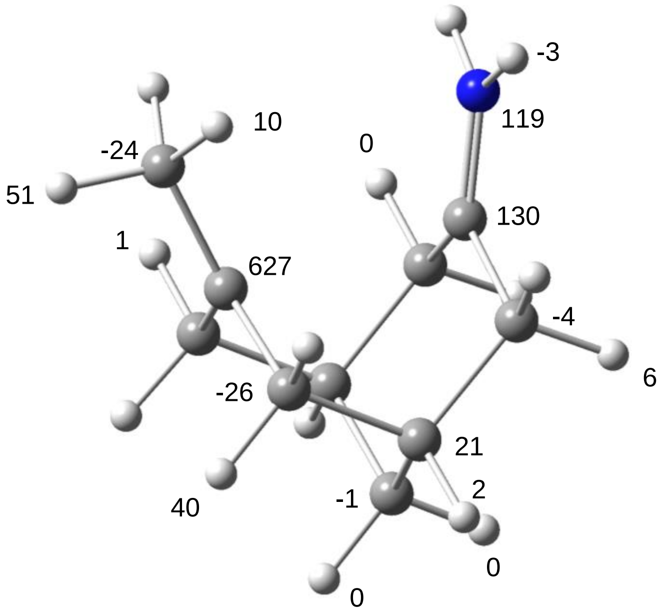

Ama$^+$(**III**) (C_s)

Figure S8

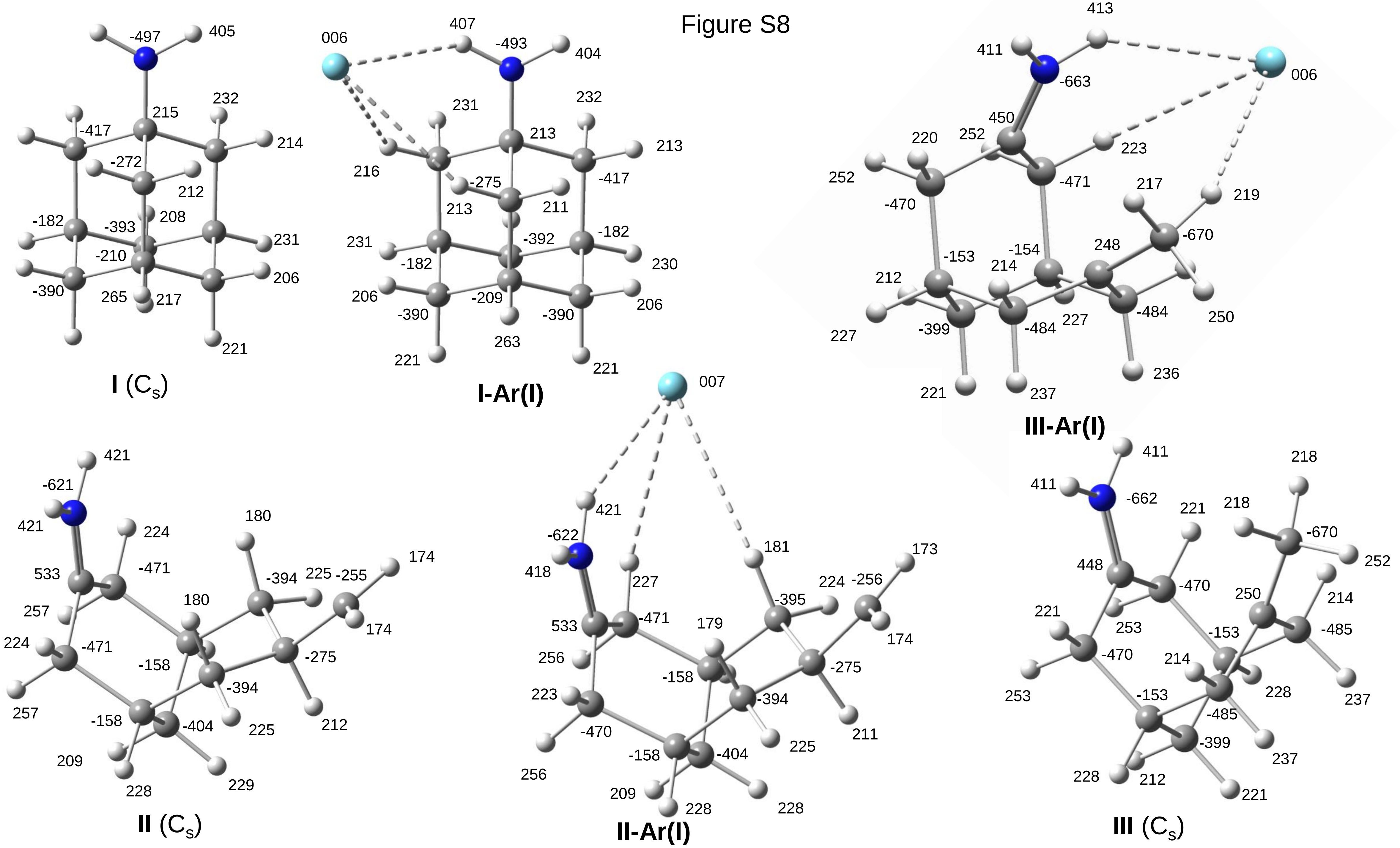

Figure S9

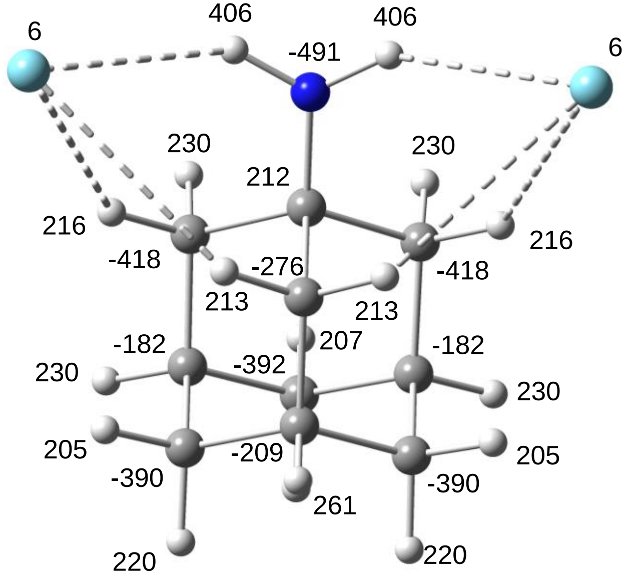

I-Ar$_2$(I) (C_s)

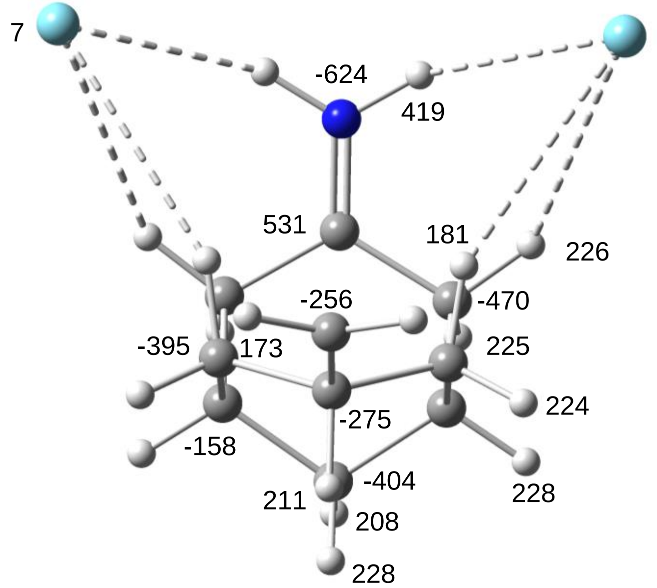

II-Ar$_2$(I) (C_S)

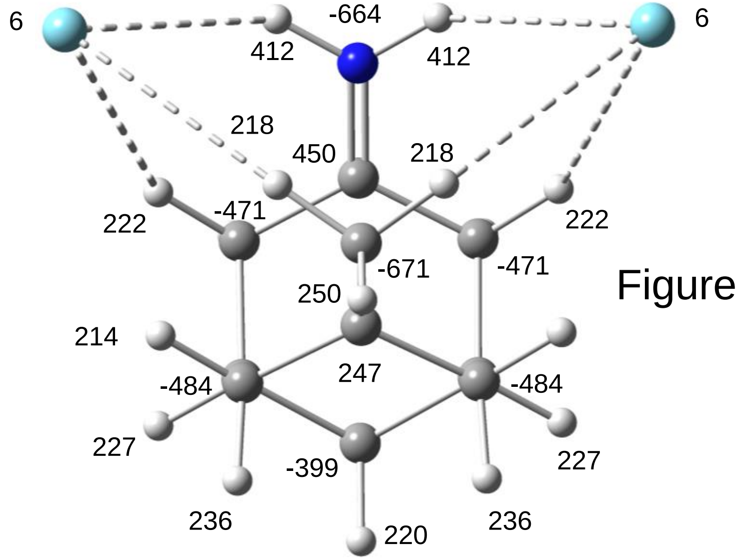

III-Ar$_2$(I) (C_S)

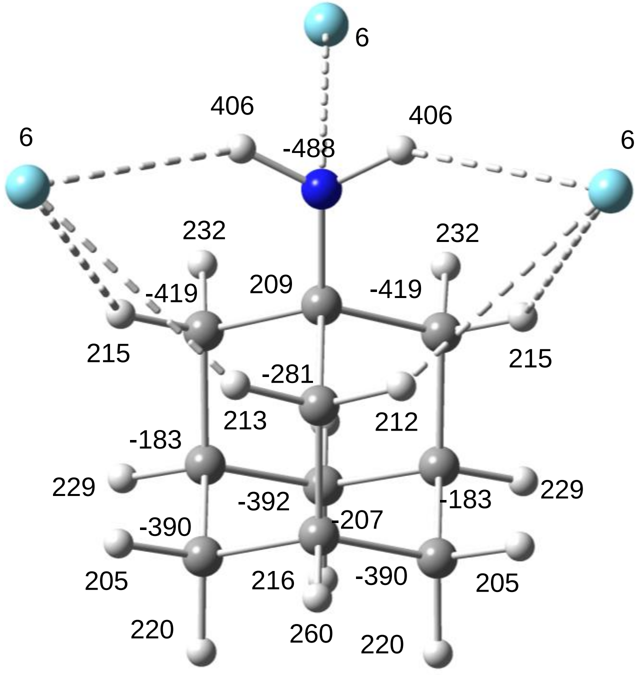

I-Ar$_3$(I)

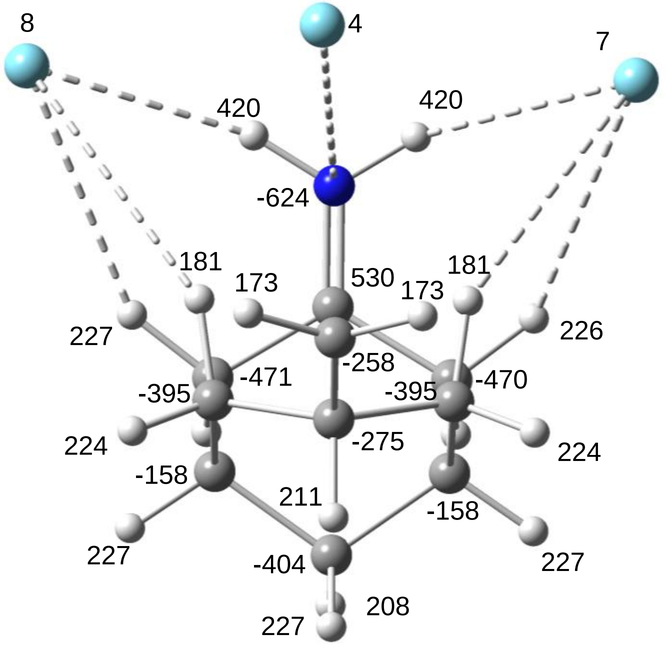

II-Ar$_3$(I)

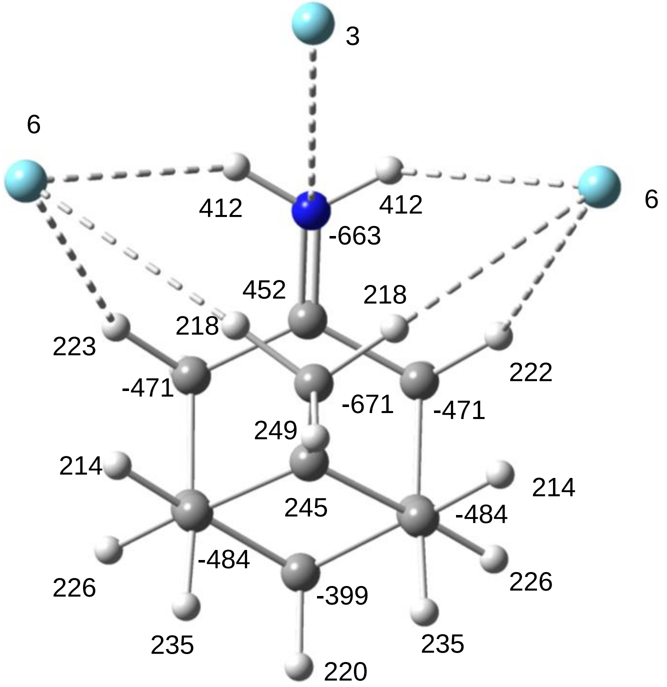

III-Ar$_3$(I) (C_S)

Figure S10

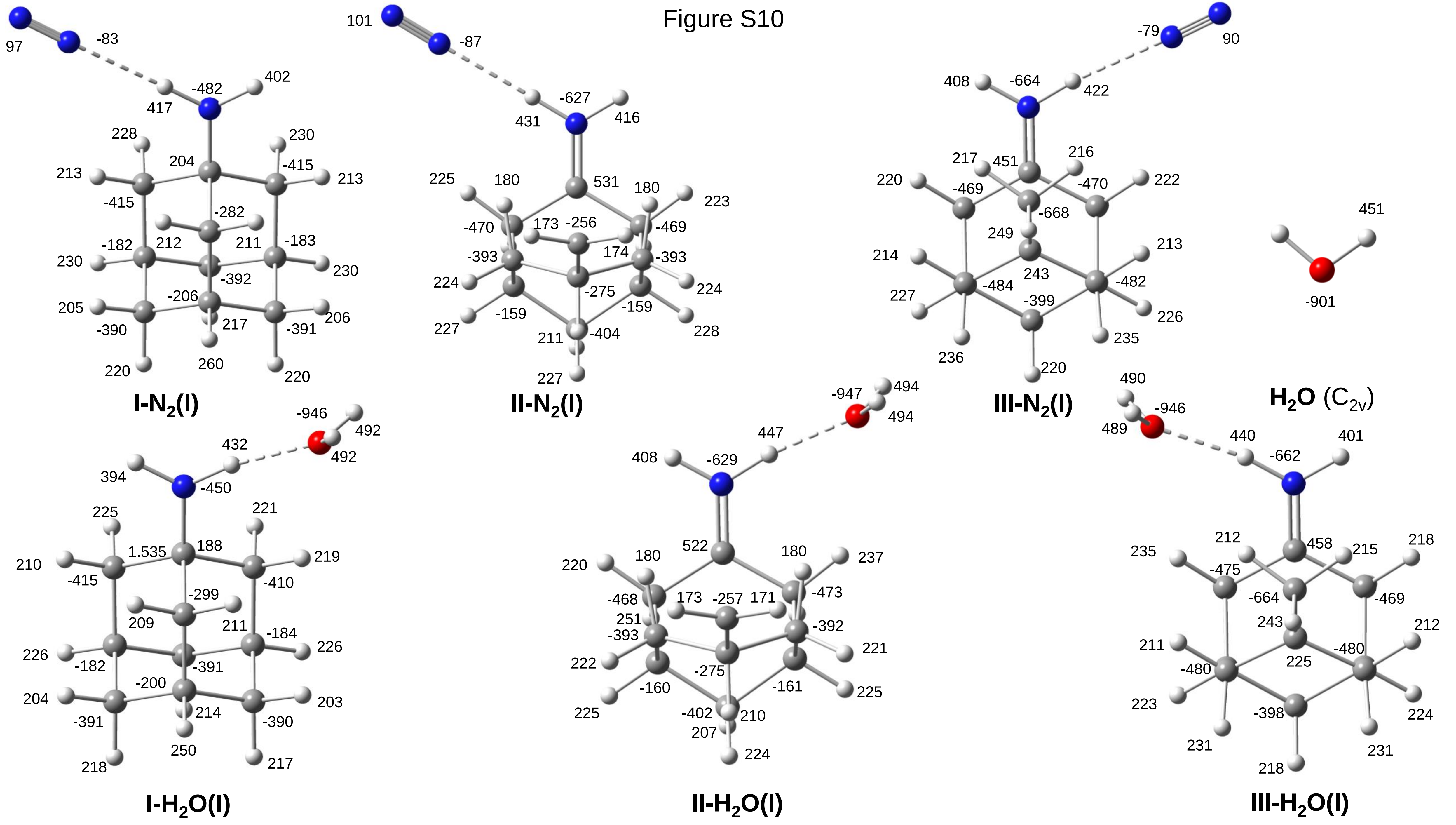

Figure S11

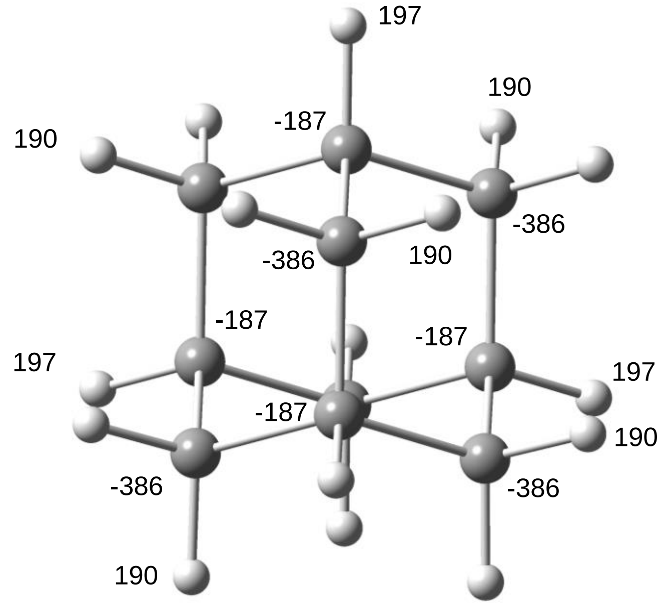

Ada (T_d)

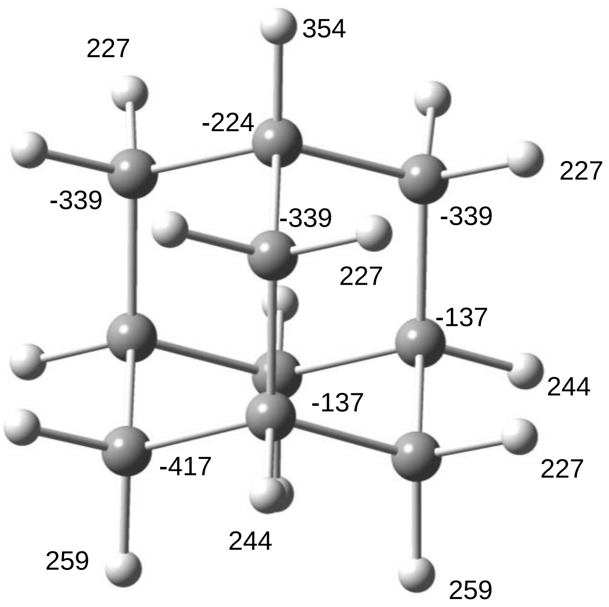

Ada$^+$(**I**) (C_{3v})

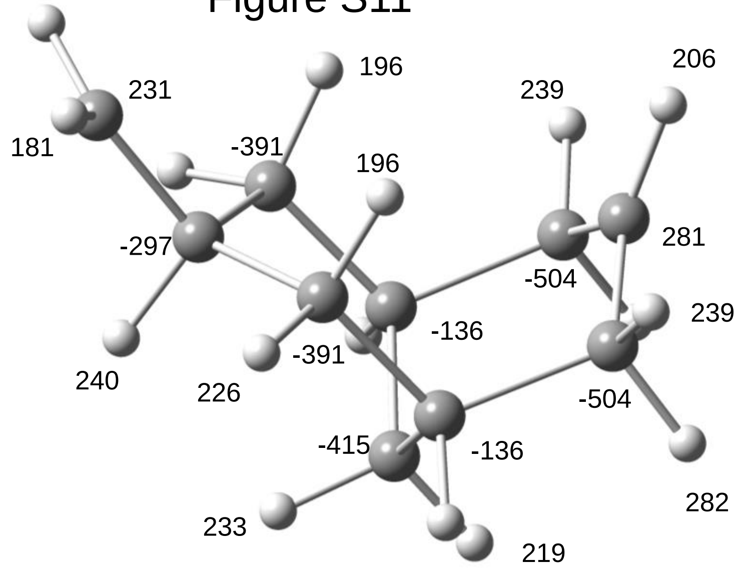

Ada$^+$(TS **I-II**) (C_s)

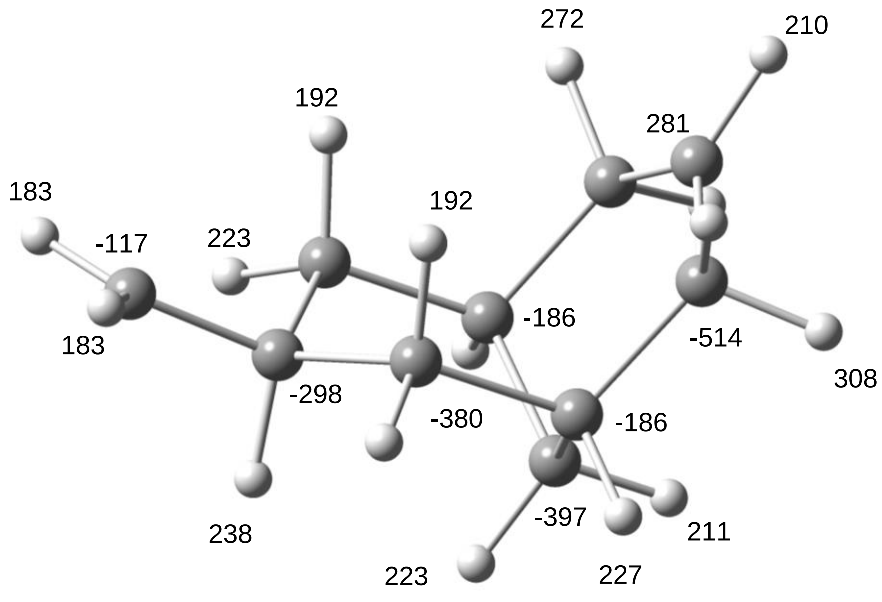

Ada$^+$(**II**) (C_s)

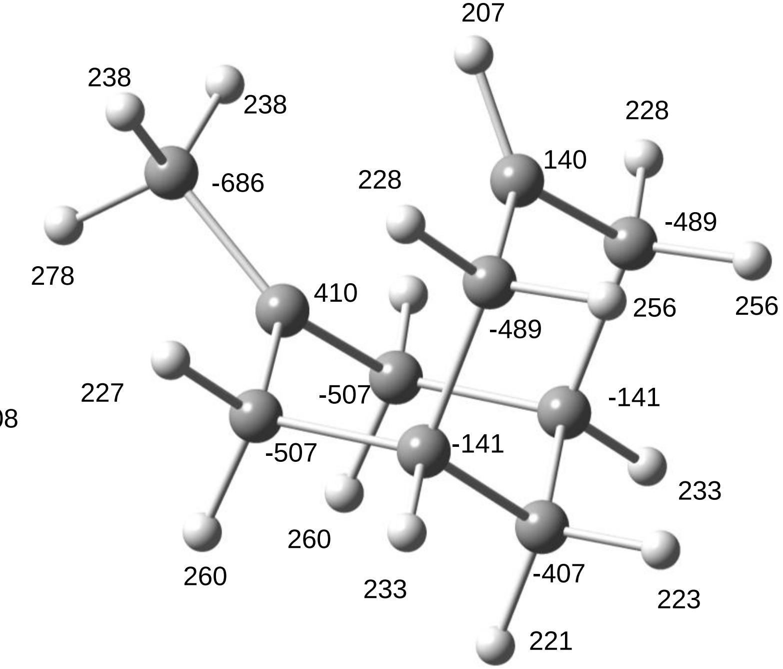

Ada$^+$(**III**) (C_s)

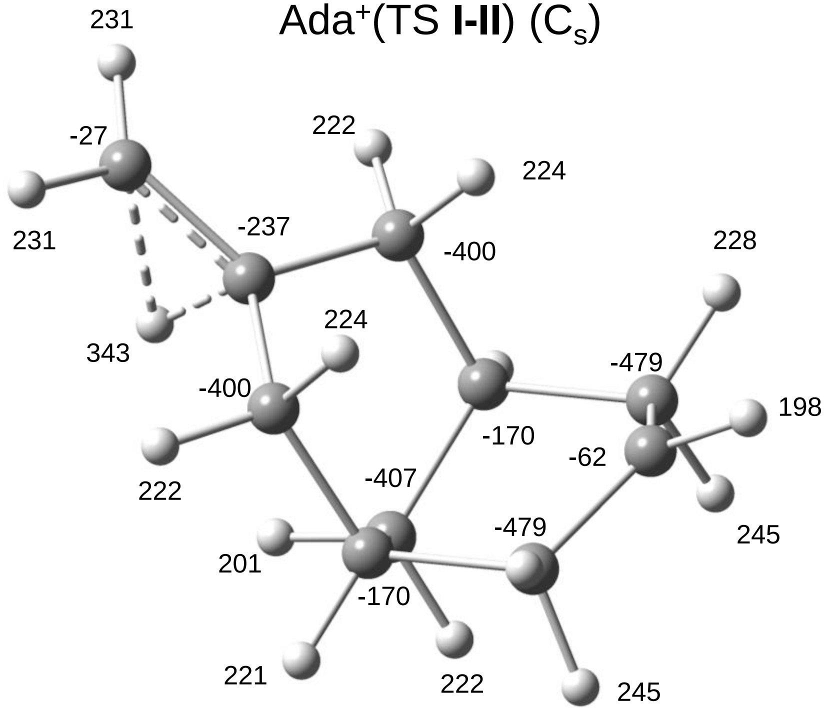

Ada$^+$(TS **II-III**) (C_s)

Figure S12

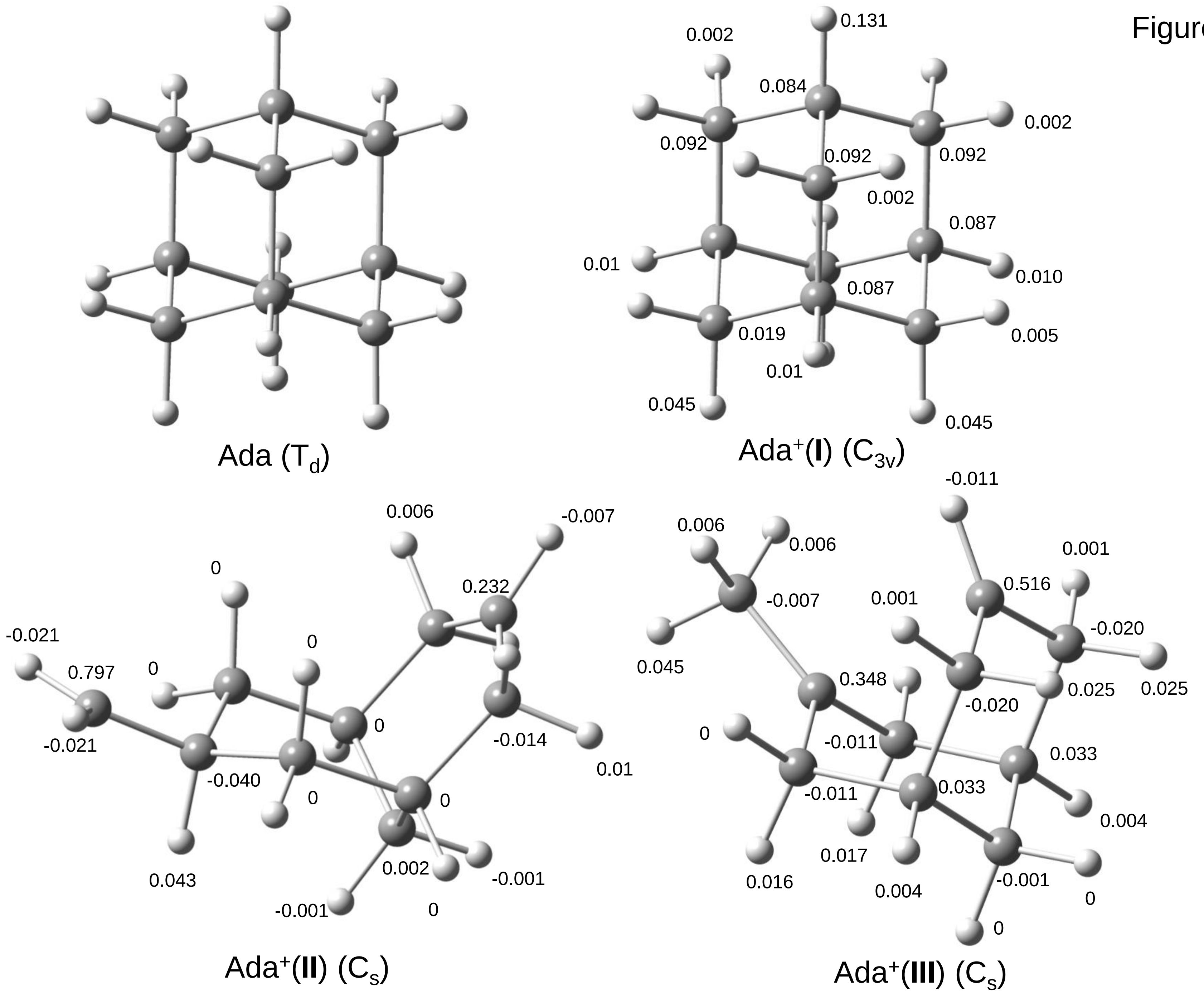
Ada (T_d)
0.131
0.002
0.084
0.002
0.092
0.092
0.092
0.002
0.087
0.01
0.010
0.087
0.019
0.005
0.01
0.045
0.045
Ada$^+$(**I**) (C_{3v})
0.006
-0.007
0
0.232
-0.021
0
0.797
0
0
-0.021
-0.014
0.01
-0.040
0
0
0.043
0.002
-0.001
-0.001
0
Ada$^+$(**II**) (C_s)
-0.011
0.006
0.006
0.001
0.516
0.001
-0.007
-0.020
0.045
0.348
0.025
0.025
-0.020
0
-0.011
0.033
-0.011
0.033
0.004
0.017
0.016
0.004
-0.001
0
0
Ada$^+$(**III**) (C_s)

Figure S13

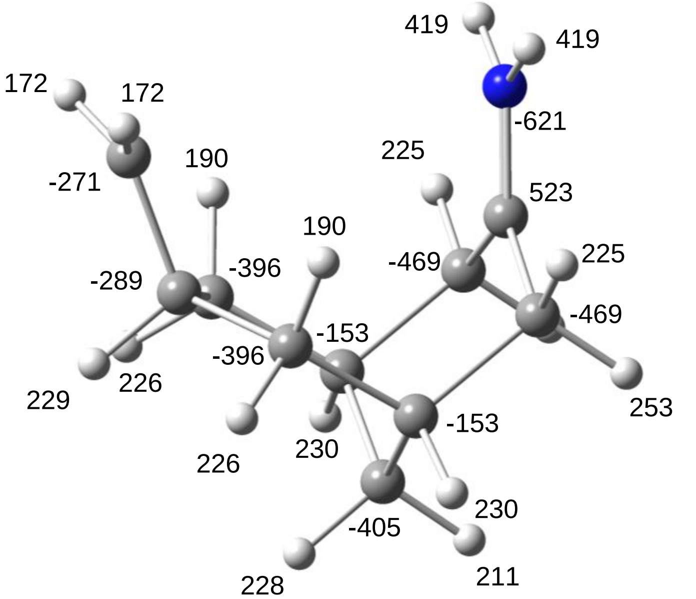

TS(I-II) (C_s)

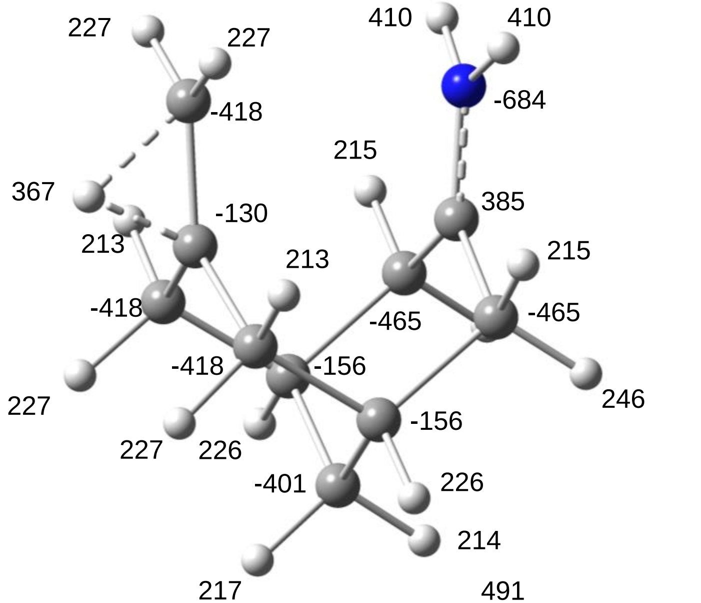

TS(II-III) (C_s)

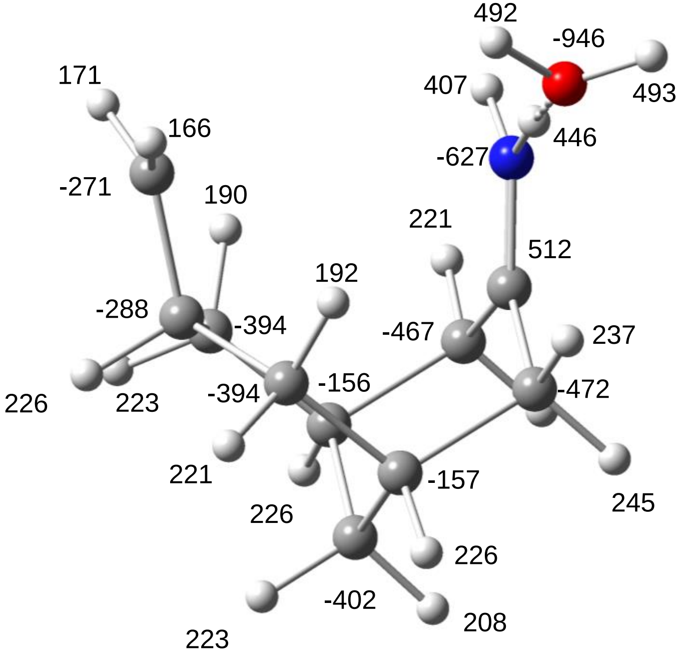

TS(I-II)-H_2O

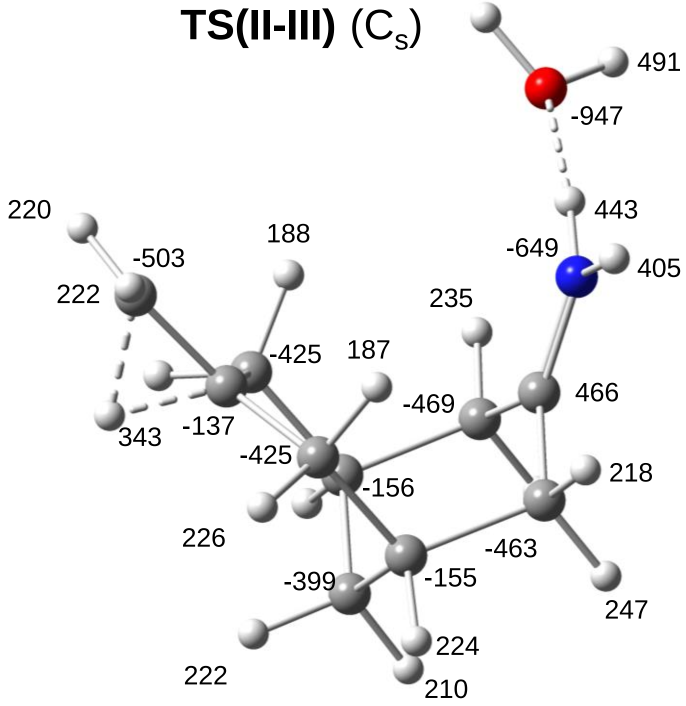

TS(II-III)-H_2O

Figure S14

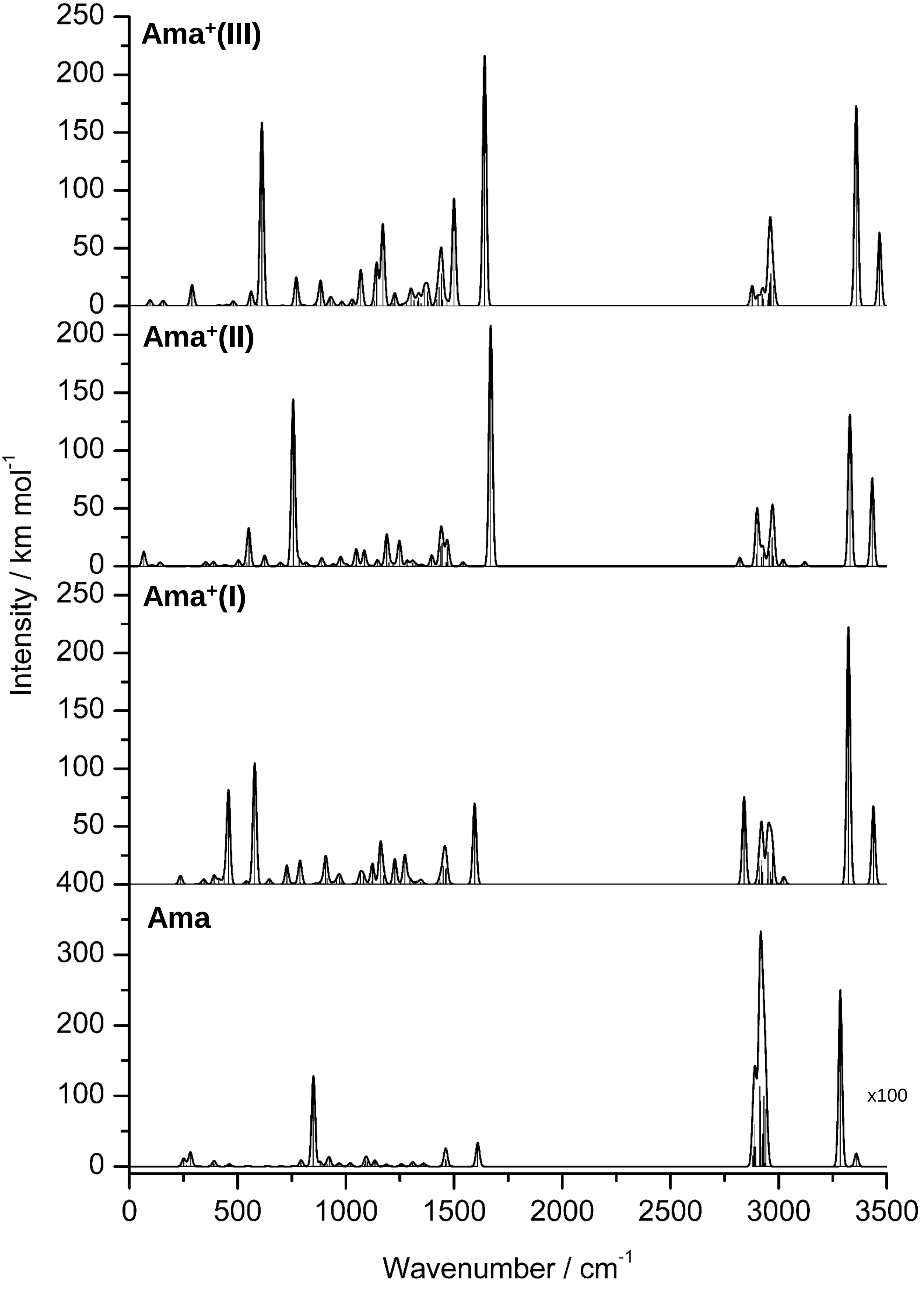

Ama+(III)
Ama+(II)
Ama+(I)
Ama
x100
Intensity / km mol-1
Wavenumber / cm-1
0
500
1000
1500
2000
2500
3000
3500

Figure S15

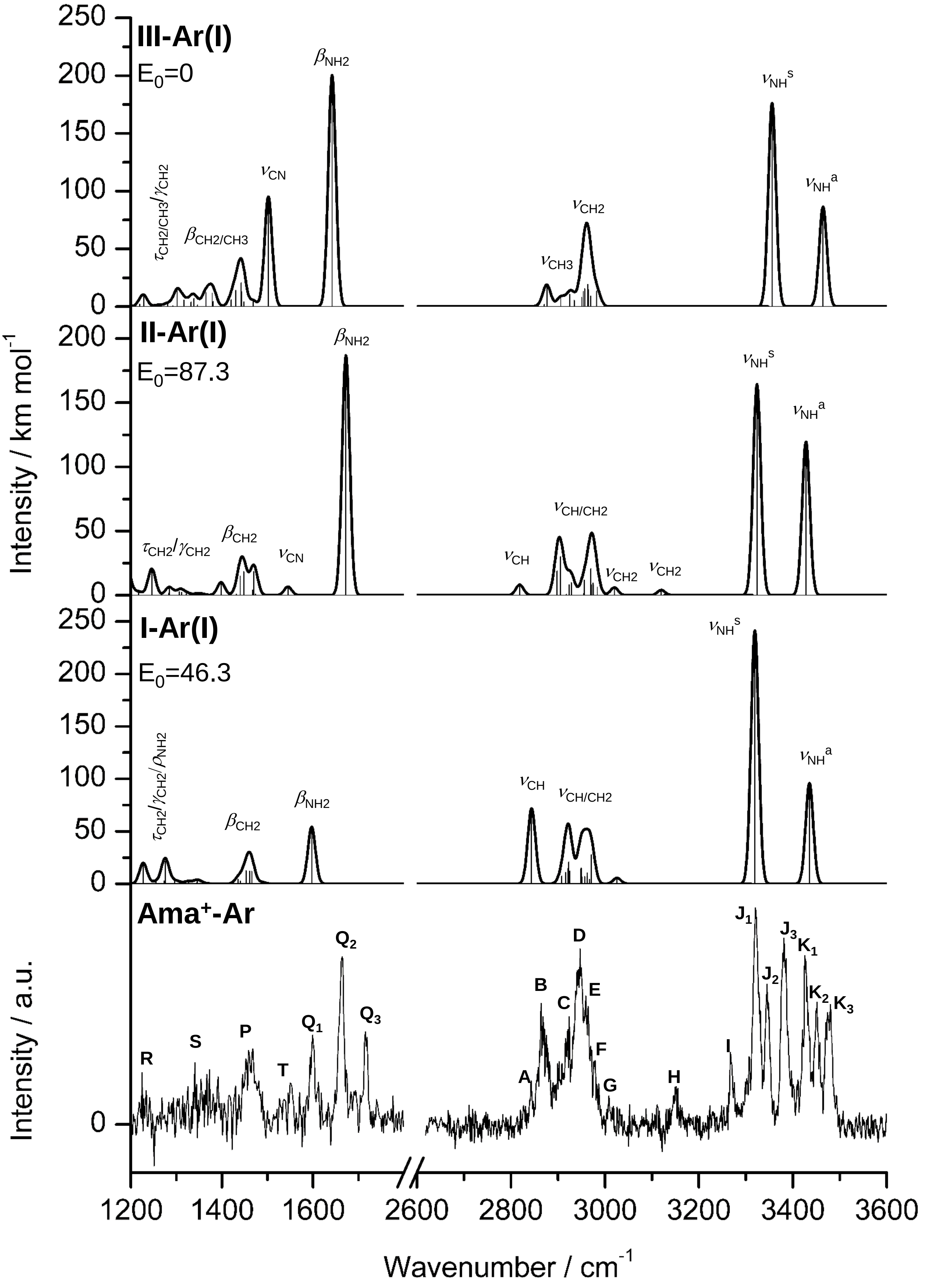
III-Ar(I)
E0=0
βNH2
νCN
τCH2/CH3/γCH2
βCH2/CH3
νCH3
νCH2
νNHs
νNHa
II-Ar(I)
E0=87.3
βNH2
τCH2/γCH2
βCH2
νCN
νCH
νCH/CH2
νCH2
νCH2
νNHs
νNHa
I-Ar(I)
E0=46.3
τCH2/γCH2/ρNH2
βCH2
βNH2
νCH
νCH/CH2
νNHs
νNHa
Ama+-Ar
R
S
P
T
Q1
Q2
Q3
A
B
C
D
E
F
G
H
I
J1
J2
J3
K1
K2
K3
Intensity / km mol-1
Intensity / a.u.
Wavenumber / cm-1

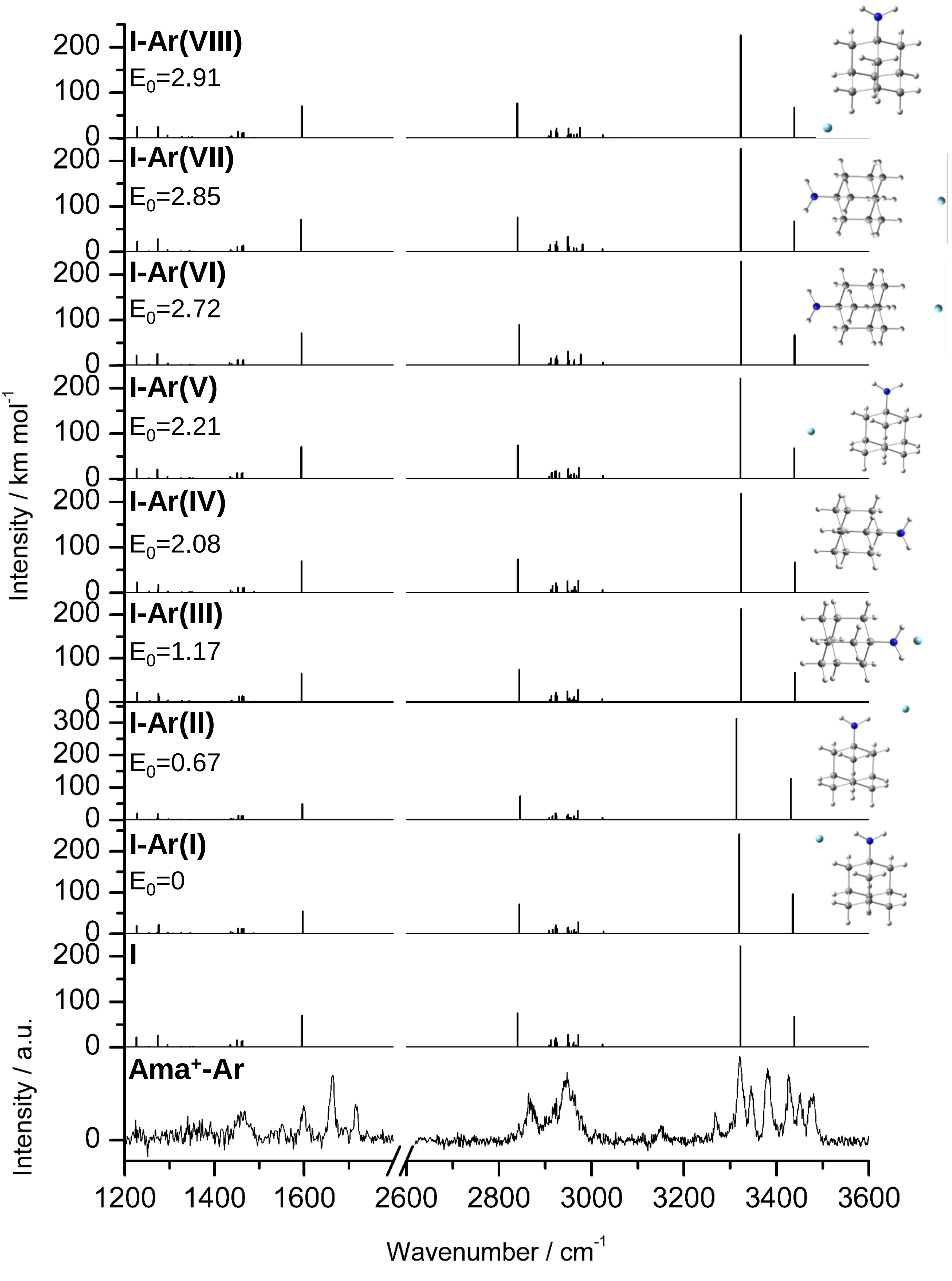
I-Ar(VIII)
E0=2.91
I-Ar(VII)
E0=2.85
I-Ar(VI)
E0=2.72
I-Ar(V)
E0=2.21
I-Ar(IV)
E0=2.08
I-Ar(III)
E0=1.17
I-Ar(II)
E0=0.67
I-Ar(I)
E0=0
I
Ama+-Ar
Intensity / km mol-1
Intensity / a.u.
Wavenumber / cm-1
1200 1400 1600 2600 2800 3000 3200 3400 3600
0 100 200 300

Figure S17

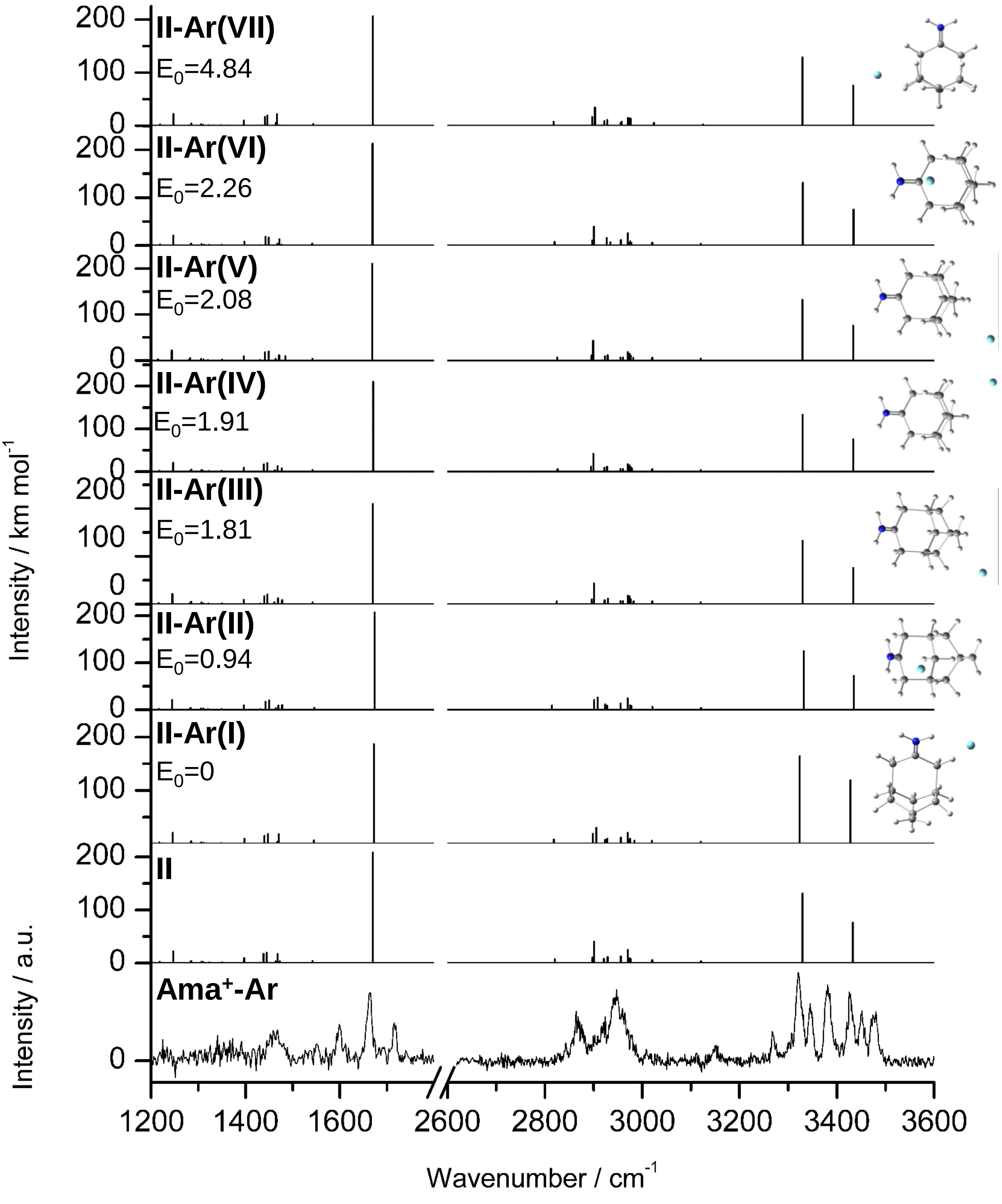
II-Ar(VII)
E0=4.84
II-Ar(VI)
E0=2.26
II-Ar(V)
E0=2.08
II-Ar(IV)
E0=1.91
II-Ar(III)
E0=1.81
II-Ar(II)
E0=0.94
II-Ar(I)
E0=0
II
Ama+-Ar
Intensity / km mol-1
Intensity / a.u.
Wavenumber / cm-1
0
100
200
1200
1400
1600
2600
2800
3000
3200
3400
3600

Figure S18

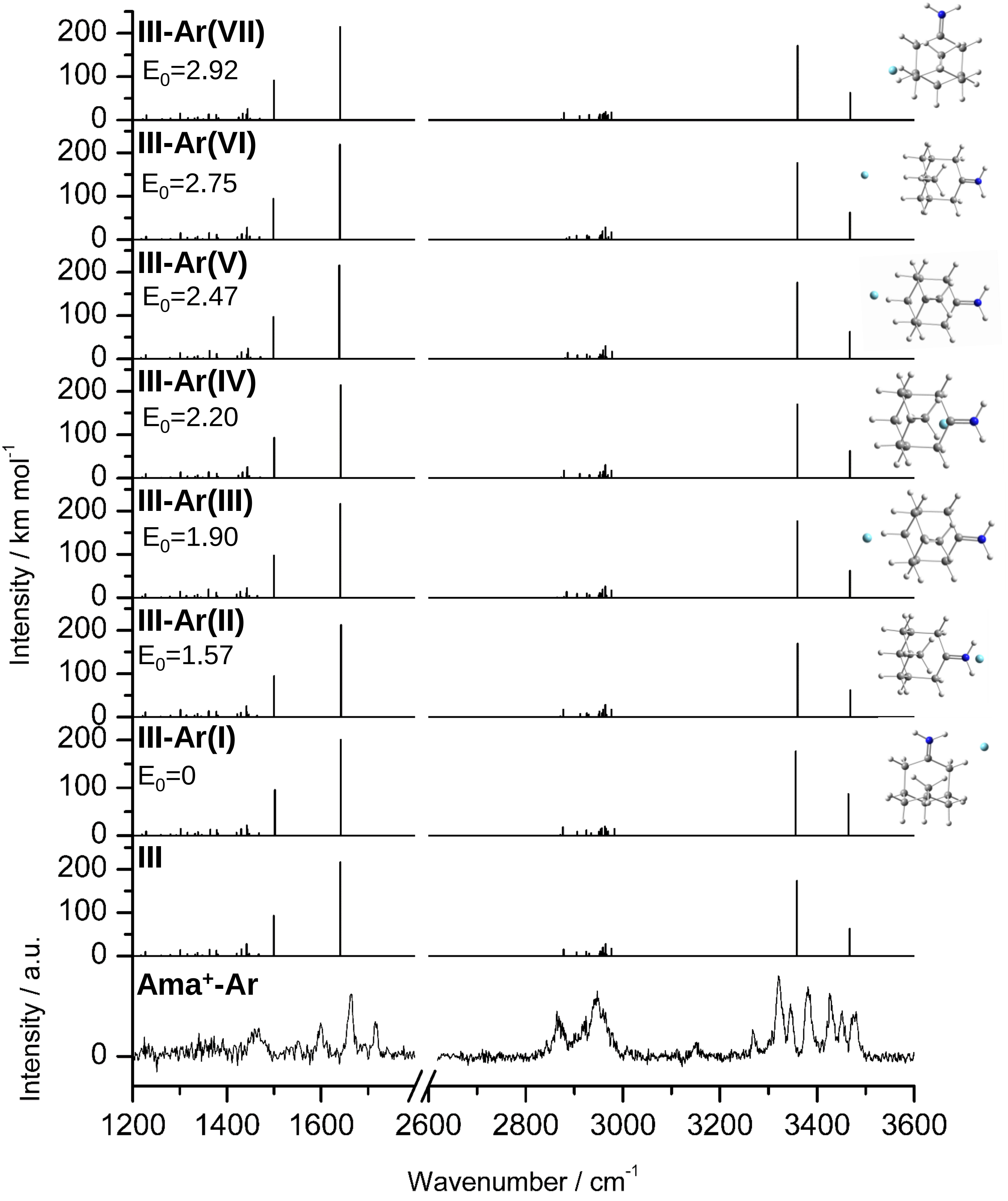
III-Ar(VII)
E0=2.92
III-Ar(VI)
E0=2.75
III-Ar(V)
E0=2.47
III-Ar(IV)
E0=2.20
III-Ar(III)
E0=1.90
III-Ar(II)
E0=1.57
III-Ar(I)
E0=0
III
Ama+-Ar
Intensity / km mol-1
Intensity / a.u.
Wavenumber / cm-1
0
100
200
1200
1400
1600
2600
2800
3000
3200
3400
3600

Figure S19

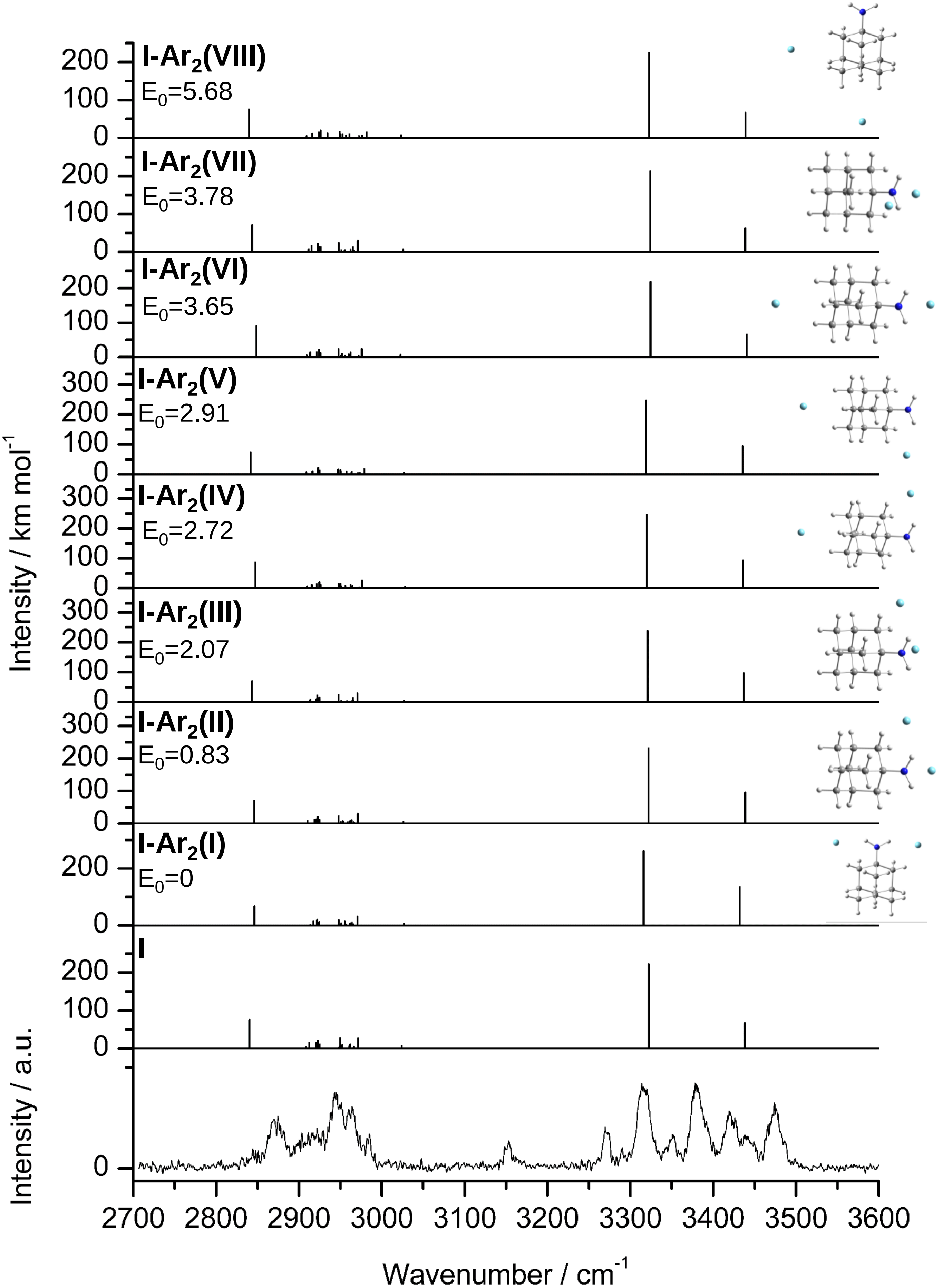

I-Ar2(VIII)
E0=5.68
I-Ar2(VII)
E0=3.78
I-Ar2(VI)
E0=3.65
I-Ar2(V)
E0=2.91
I-Ar2(IV)
E0=2.72
I-Ar2(III)
E0=2.07
I-Ar2(II)
E0=0.83
I-Ar2(I)
E0=0
I
Intensity / km mol-1
Intensity / a.u.
2700 2800 2900 3000 3100 3200 3300 3400 3500 3600
Wavenumber / cm-1

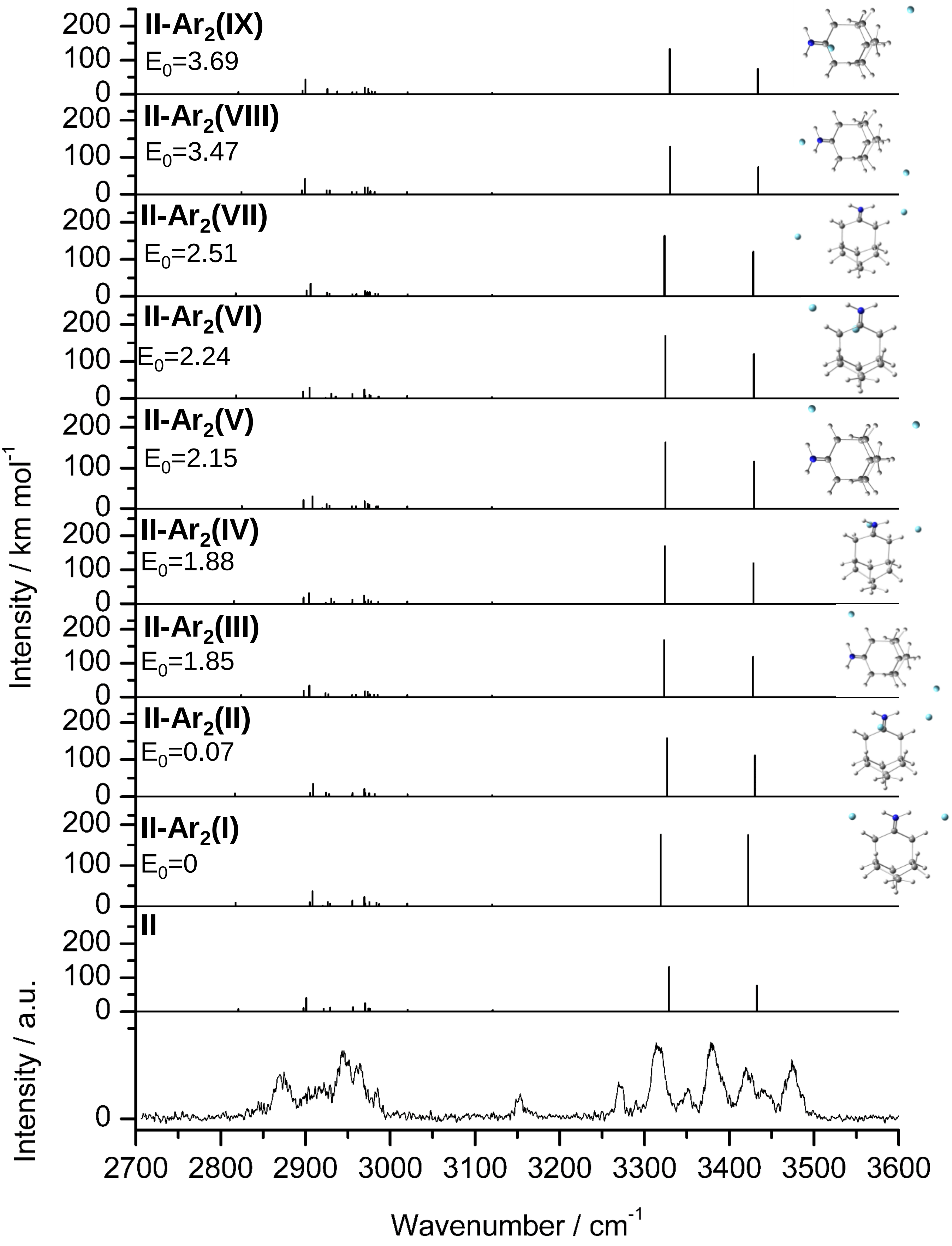
II-Ar$_2$(IX)
E$_0$=3.69
II-Ar$_2$(VIII)
E$_0$=3.47
II-Ar$_2$(VII)
E$_0$=2.51
II-Ar$_2$(VI)
E$_0$=2.24
II-Ar$_2$(V)
E$_0$=2.15
II-Ar$_2$(IV)
E$_0$=1.88
II-Ar$_2$(III)
E$_0$=1.85
II-Ar$_2$(II)
E$_0$=0.07
II-Ar$_2$(I)
E$_0$=0
II
Intensity / km mol^{-1}
Intensity / a.u.
Wavenumber / cm^{-1}
2700 2800 2900 3000 3100 3200 3300 3400 3500 3600
0 100 200

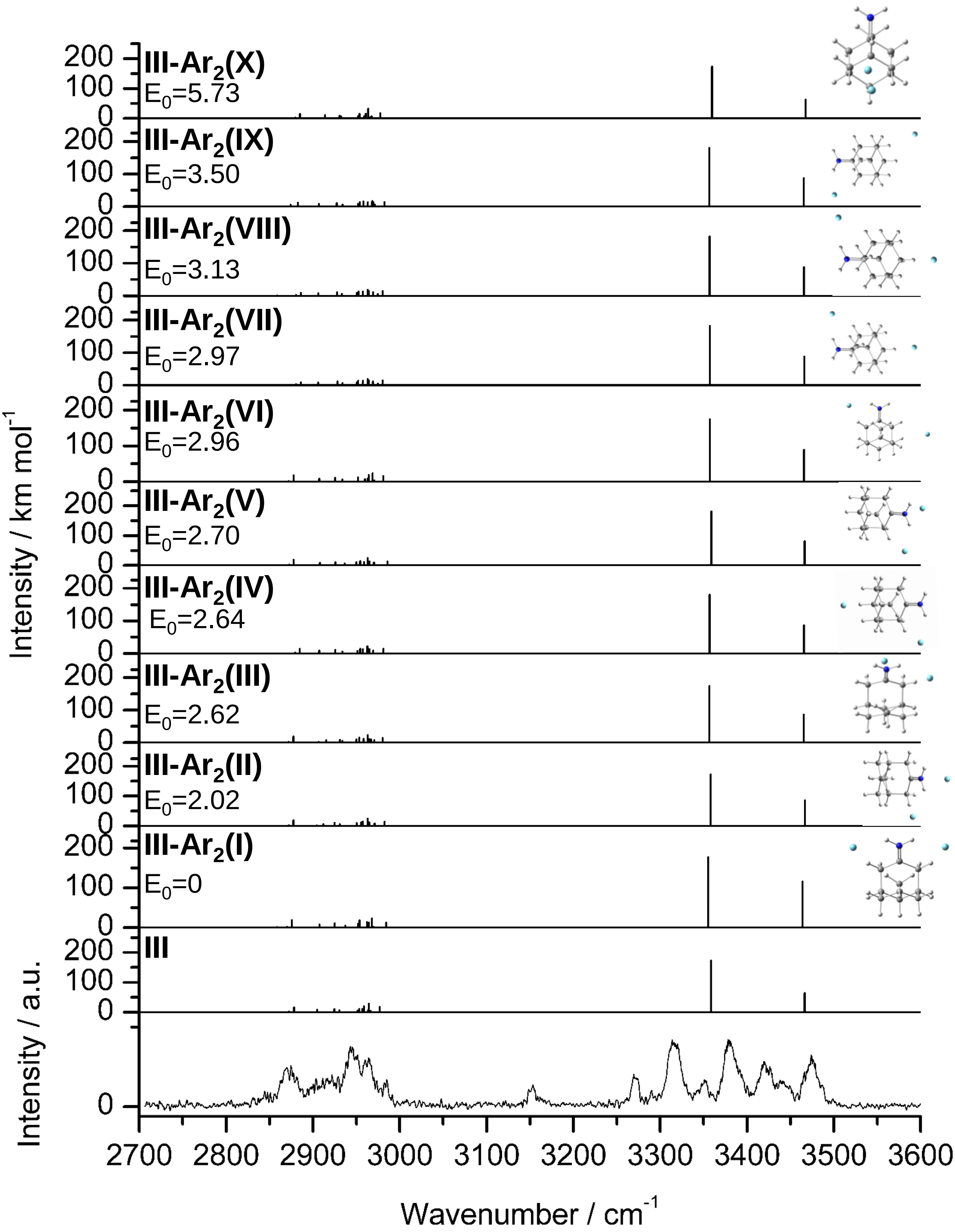
III-Ar2(X)
E0=5.73
III-Ar2(IX)
E0=3.50
III-Ar2(VIII)
E0=3.13
III-Ar2(VII)
E0=2.97
III-Ar2(VI)
E0=2.96
III-Ar2(V)
E0=2.70
III-Ar2(IV)
E0=2.64
III-Ar2(III)
E0=2.62
III-Ar2(II)
E0=2.02
III-Ar2(I)
E0=0
III
Intensity / km mol-1
Intensity / a.u.
0
100
200
2700
2800
2900
3000
3100
3200
3300
3400
3500
3600
Wavenumber / cm-1

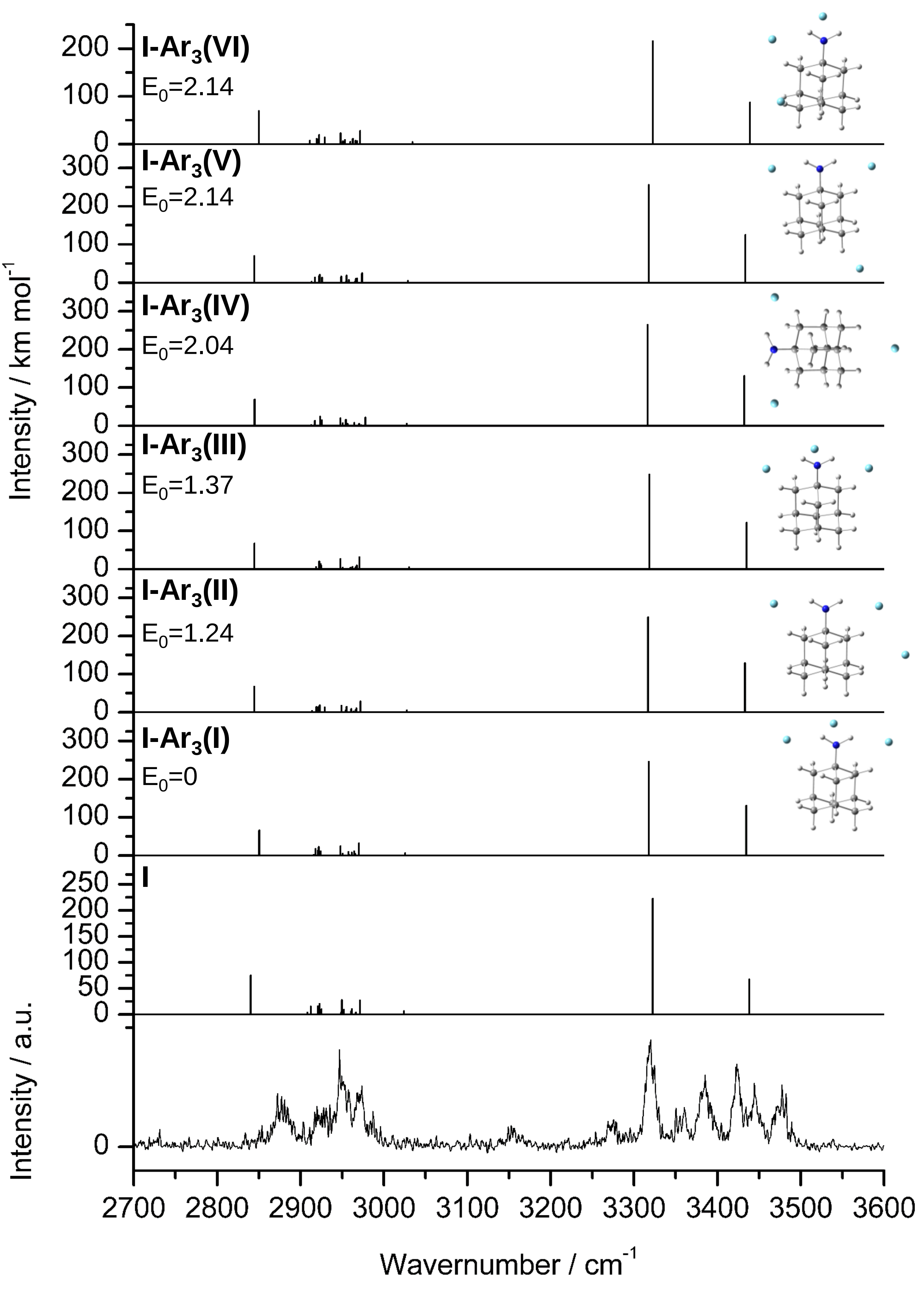
I-Ar3(VI)
E0=2.14
I-Ar3(V)
E0=2.14
I-Ar3(IV)
E0=2.04
I-Ar3(III)
E0=1.37
I-Ar3(II)
E0=1.24
I-Ar3(I)
E0=0
I
Intensity / km mol-1
Intensity / a.u.
Wavenumber / cm-1
2700 2800 2900 3000 3100 3200 3300 3400 3500 3600

Figure S23

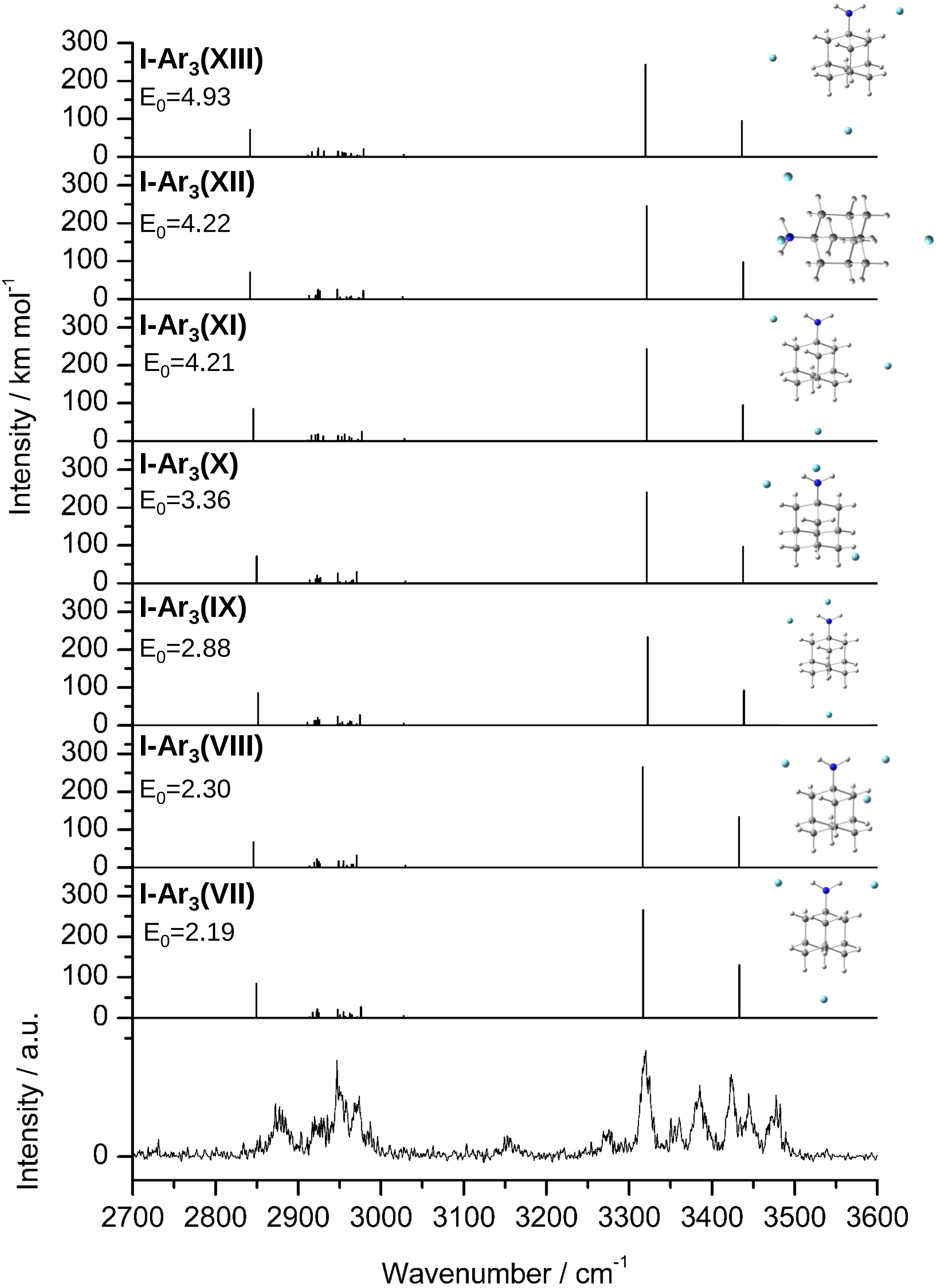

I-Ar3(XIII)
E0=4.93
I-Ar3(XII)
E0=4.22
I-Ar3(XI)
E0=4.21
I-Ar3(X)
E0=3.36
I-Ar3(IX)
E0=2.88
I-Ar3(VIII)
E0=2.30
I-Ar3(VII)
E0=2.19
Intensity / km mol-1
Intensity / a.u.
Wavenumber / cm-1
0
100
200
300
2700
2800
2900
3000
3100
3200
3300
3400
3500
3600

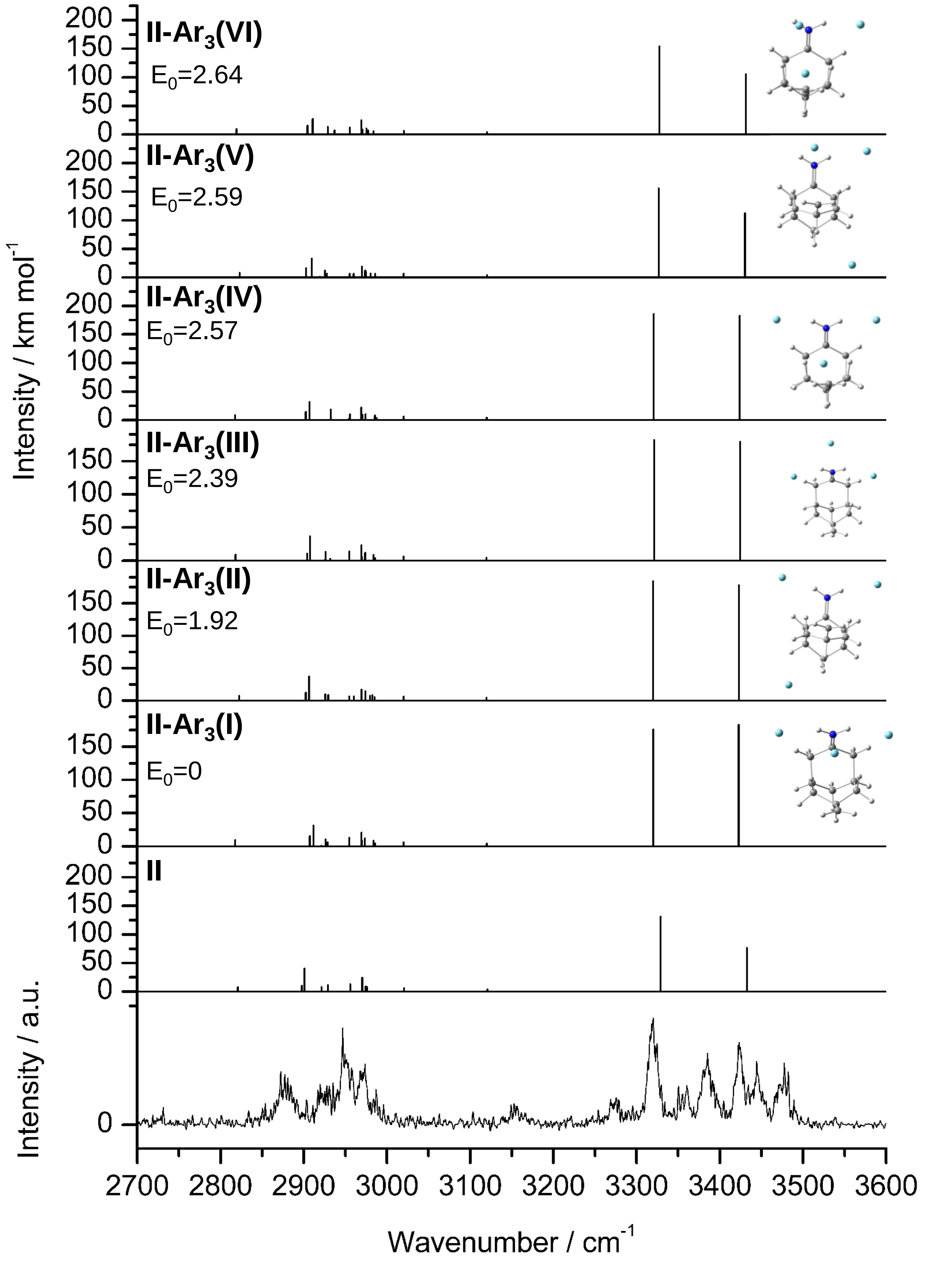

II-Ar3(VI)
E0=2.64
II-Ar3(V)
E0=2.59
II-Ar3(IV)
E0=2.57
II-Ar3(III)
E0=2.39
II-Ar3(II)
E0=1.92
II-Ar3(I)
E0=0
II
Intensity / km mol-1
Intensity / a.u.
Wavenumber / cm-1
2700 2800 2900 3000 3100 3200 3300 3400 3500 3600

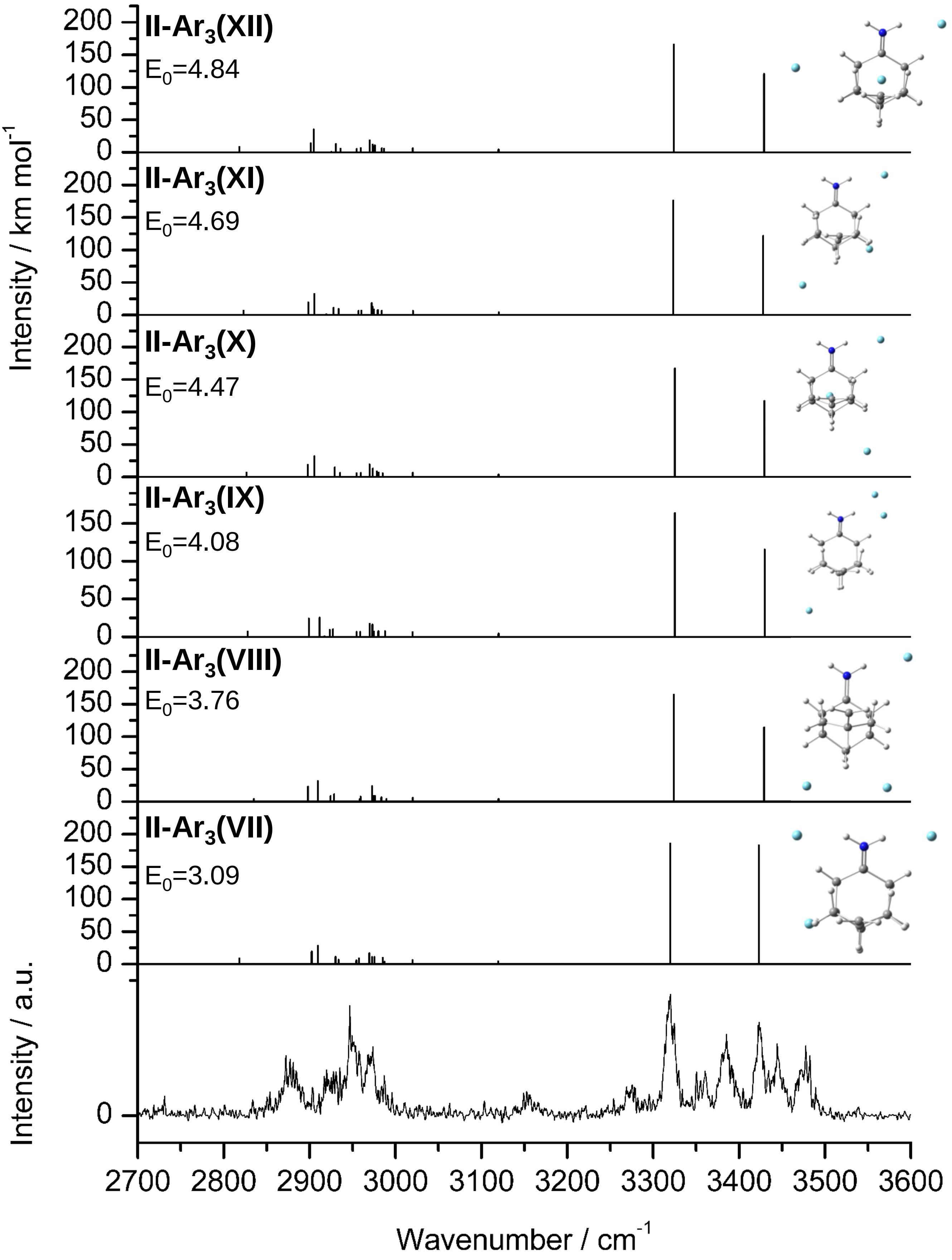
Intensity / km mol-1
II-Ar3(XII)
E0=4.84
II-Ar3(XI)
E0=4.69
II-Ar3(X)
E0=4.47
II-Ar3(IX)
E0=4.08
II-Ar3(VIII)
E0=3.76
II-Ar3(VII)
E0=3.09
Intensity / a.u.
Wavenumber / cm-1
2700
2800
2900
3000
3100
3200
3300
3400
3500
3600

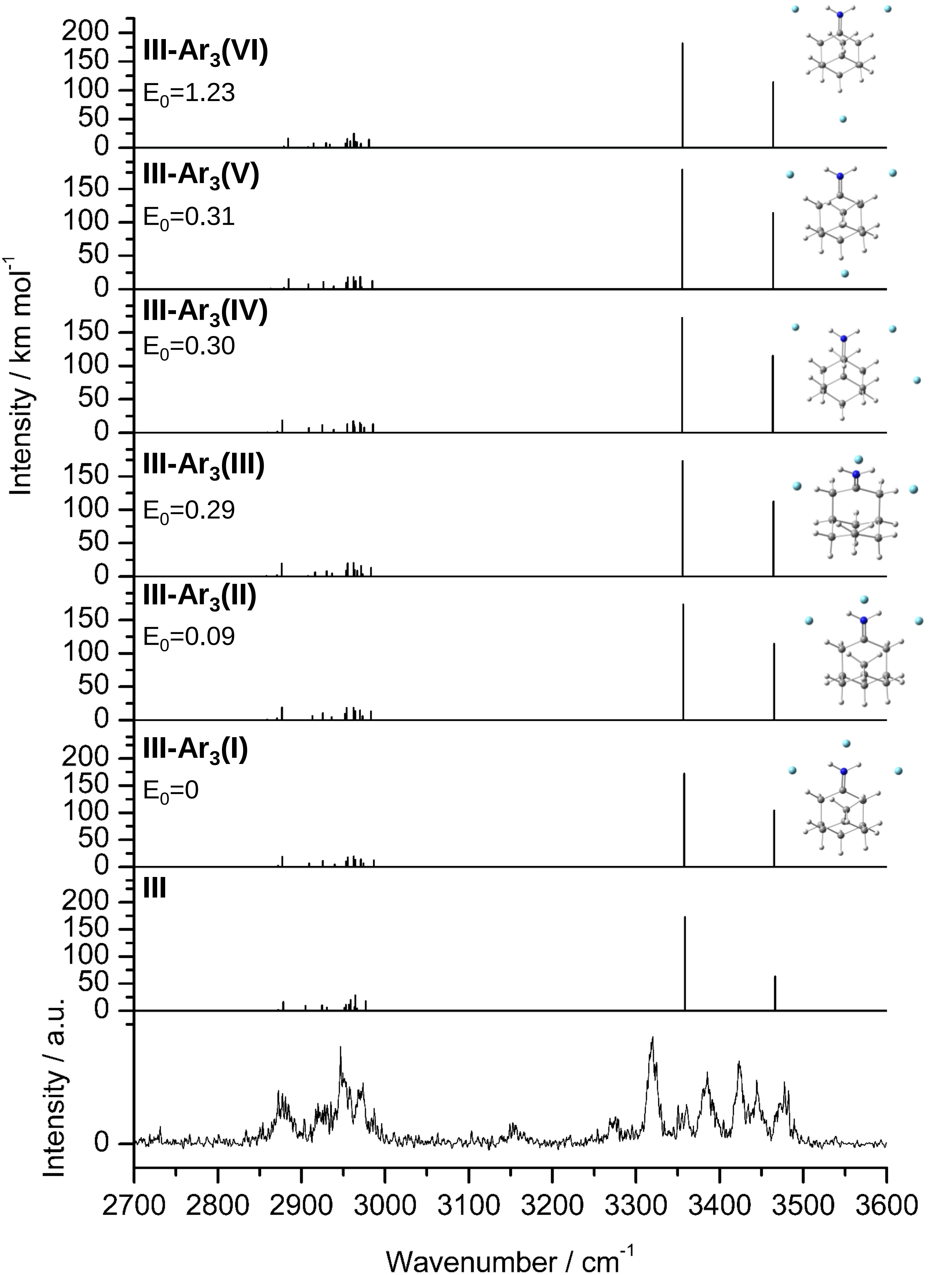
III-Ar$_3$(VI)
E$_0$=1.23
III-Ar$_3$(V)
E$_0$=0.31
III-Ar$_3$(IV)
E$_0$=0.30
III-Ar$_3$(III)
E$_0$=0.29
III-Ar$_3$(II)
E$_0$=0.09
III-Ar$_3$(I)
E$_0$=0
III
Intensity / km mol^{-1}
Intensity / a.u.
Wavenumber / cm^{-1}
2700 2800 2900 3000 3100 3200 3300 3400 3500 3600

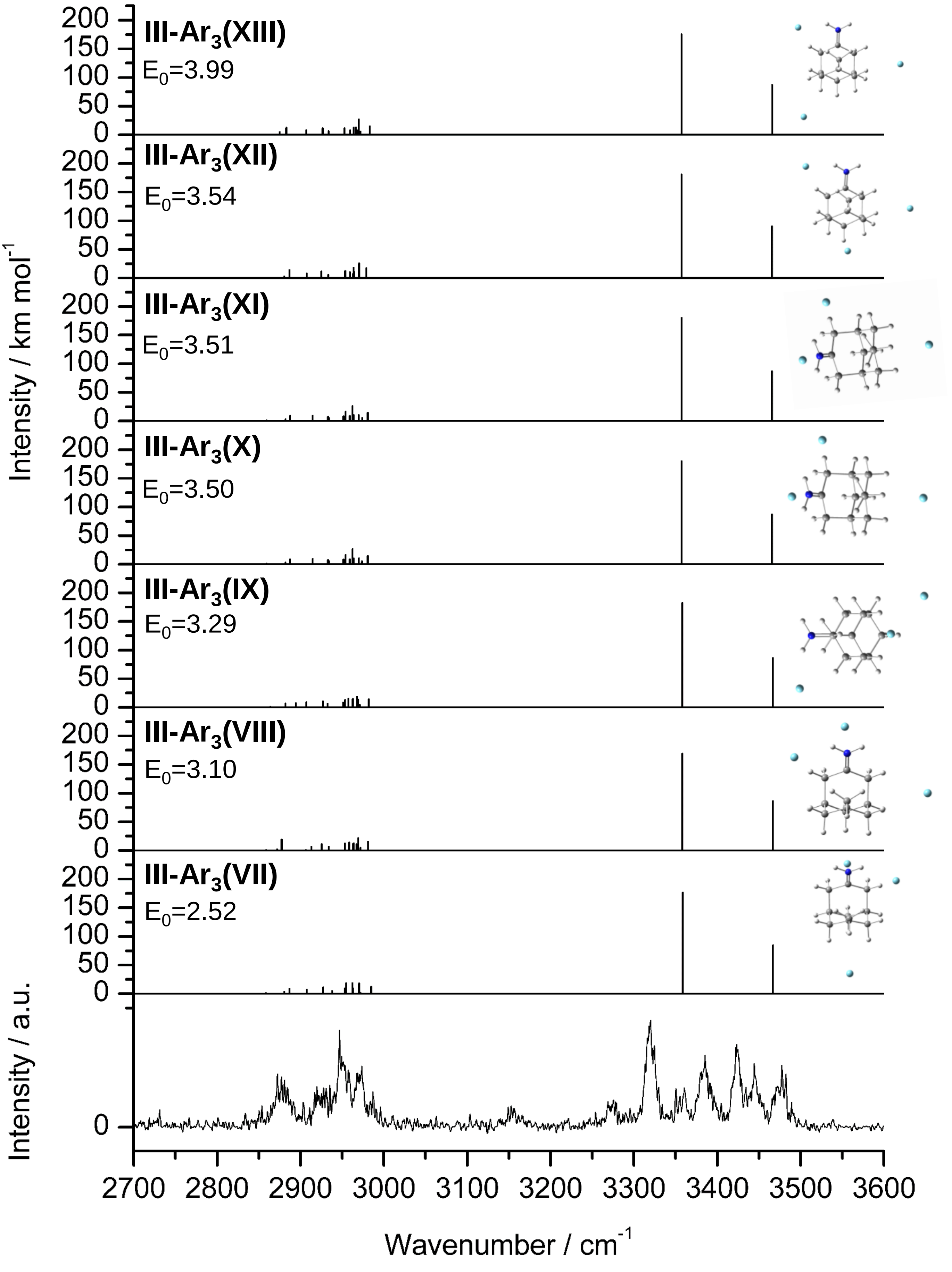
III-Ar3(XIII)
E0=3.99
III-Ar3(XII)
E0=3.54
III-Ar3(XI)
E0=3.51
III-Ar3(X)
E0=3.50
III-Ar3(IX)
E0=3.29
III-Ar3(VIII)
E0=3.10
III-Ar3(VII)
E0=2.52
Intensity / km mol-1
Intensity / a.u.
Wavenumber / cm-1
0
50
100
150
200
2700
2800
2900
3000
3100
3200
3300
3400
3500
3600

Figure S28

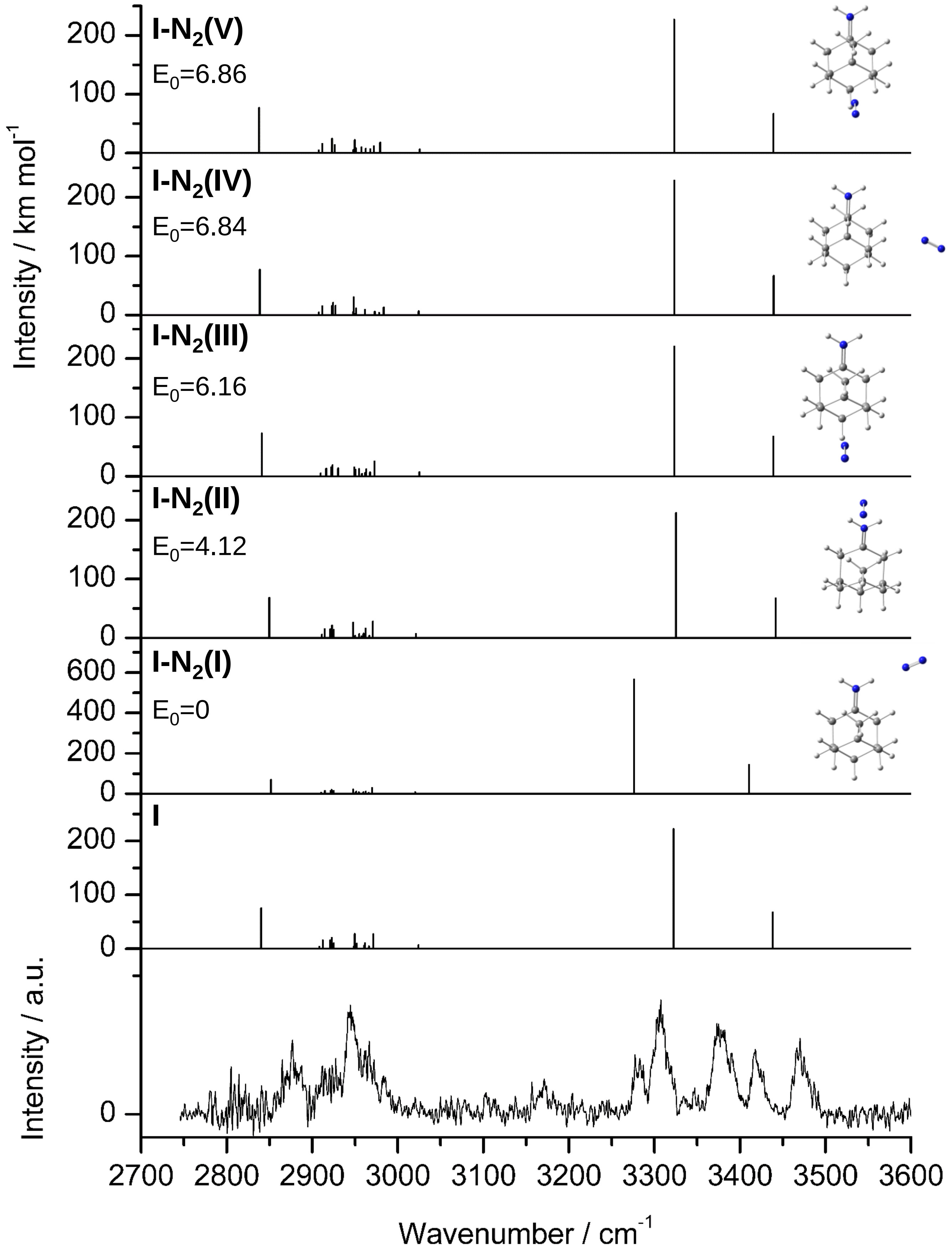
I-N2(V)
E0=6.86
I-N2(IV)
E0=6.84
I-N2(III)
E0=6.16
I-N2(II)
E0=4.12
I-N2(I)
E0=0
I
Intensity / km mol-1
Intensity / a.u.
Wavenumber / cm-1
0
100
200
400
600
2700
2800
2900
3000
3100
3200
3300
3400
3500
3600

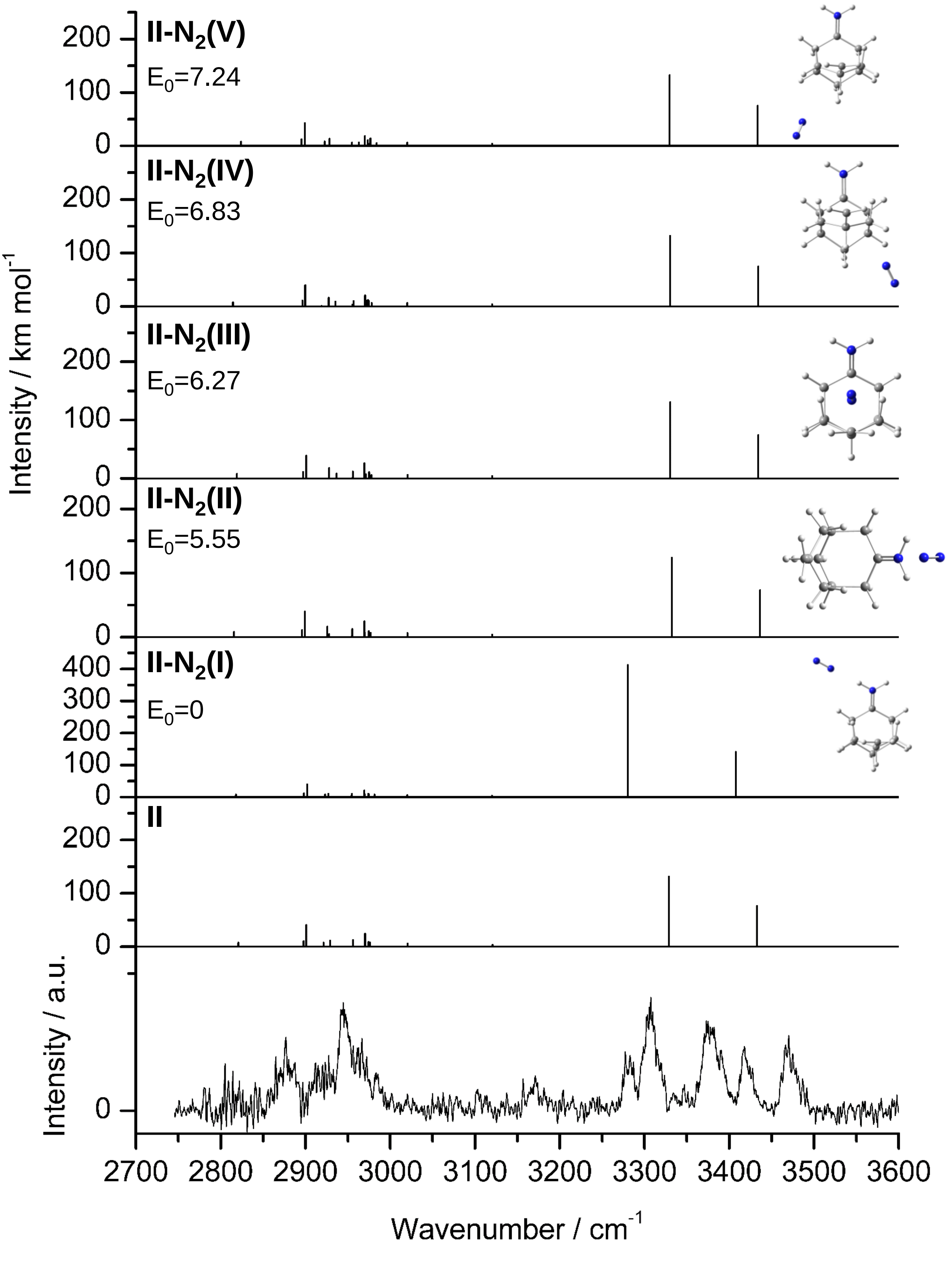
II-N2(V)
E0=7.24
II-N2(IV)
E0=6.83
II-N2(III)
E0=6.27
II-N2(II)
E0=5.55
II-N2(I)
E0=0
II
Intensity / km mol-1
Intensity / a.u.
Wavenumber / cm-1
2700 2800 2900 3000 3100 3200 3300 3400 3500 3600

Figure S30

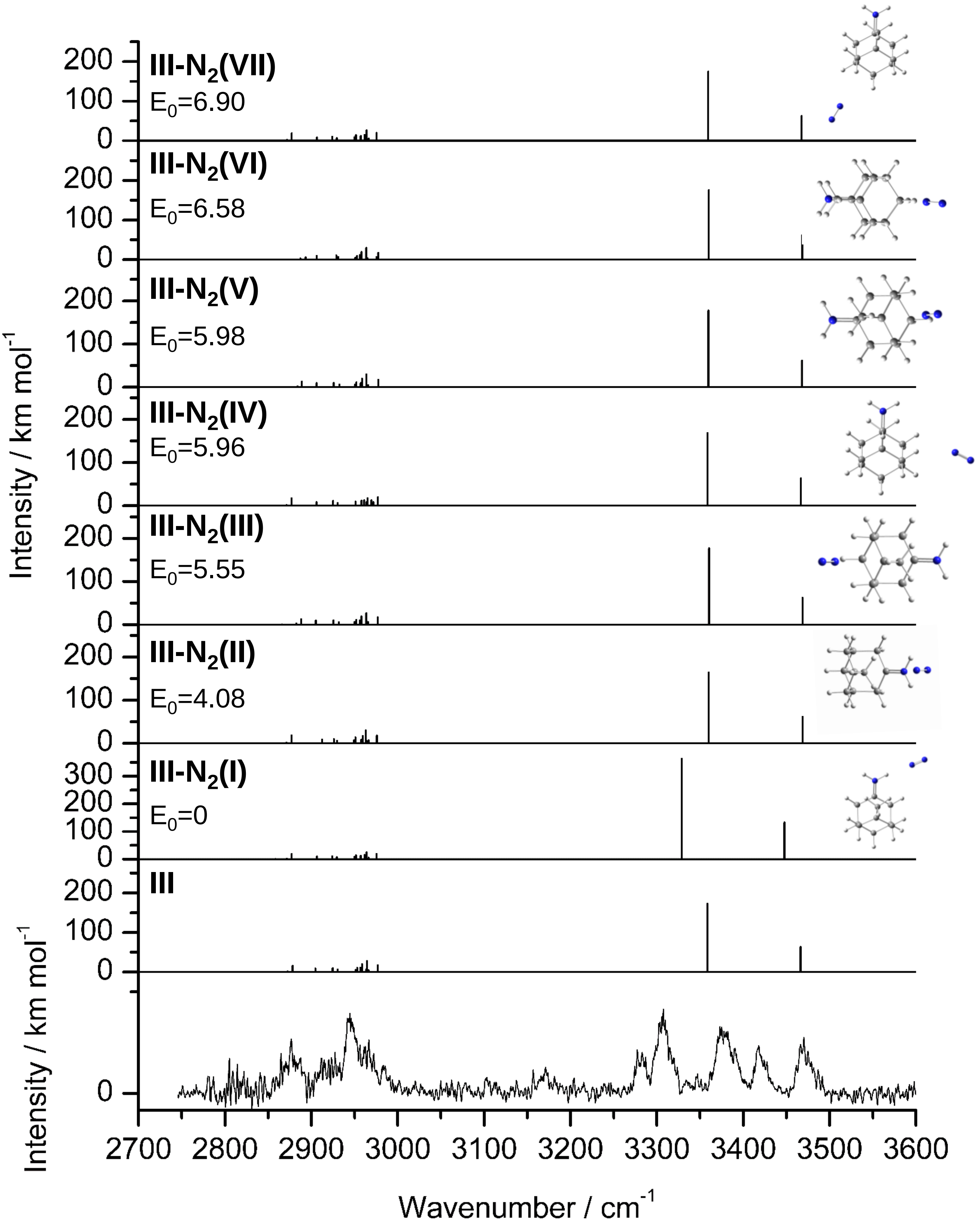

III-N$_2$(VII)
E$_0$=6.90
III-N$_2$(VI)
E$_0$=6.58
III-N$_2$(V)
E$_0$=5.98
III-N$_2$(IV)
E$_0$=5.96
III-N$_2$(III)
E$_0$=5.55
III-N$_2$(II)
E$_0$=4.08
III-N$_2$(I)
E$_0$=0
III
Intensity / km mol^{-1}
Intensity / km mol^{-1}
0
100
200
300
2700
2800
2900
3000
3100
3200
3300
3400
3500
3600
Wavenumber / cm^{-1}

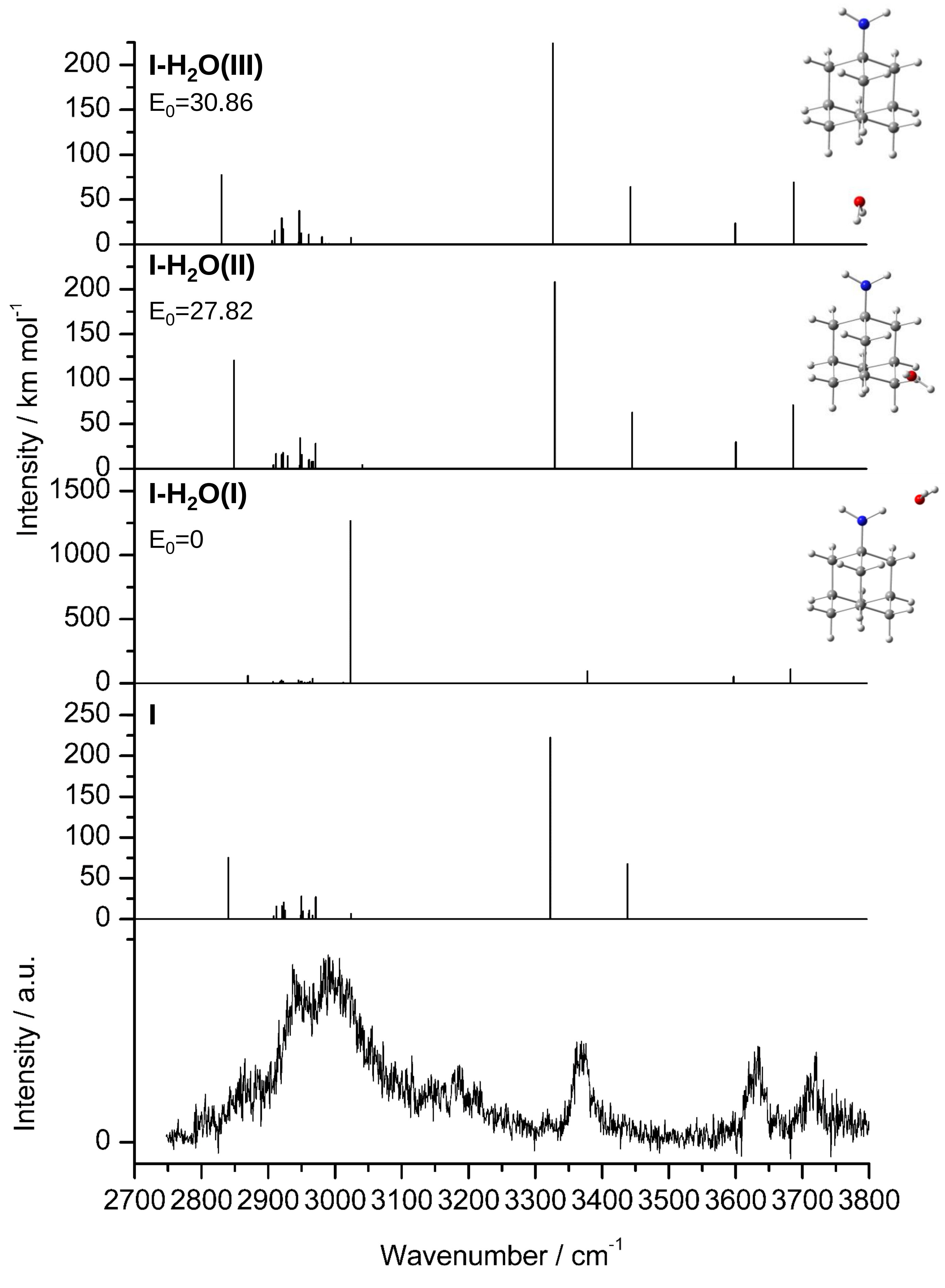
I-H_2O(III)
E_0=30.86
I-H_2O(II)
E_0=27.82
I-H_2O(I)
E_0=0
I
Intensity / km mol^{-1}
Intensity / a.u.
Wavenumber / cm^{-1}
2700 2800 2900 3000 3100 3200 3300 3400 3500 3600 3700 3800

Figure S32

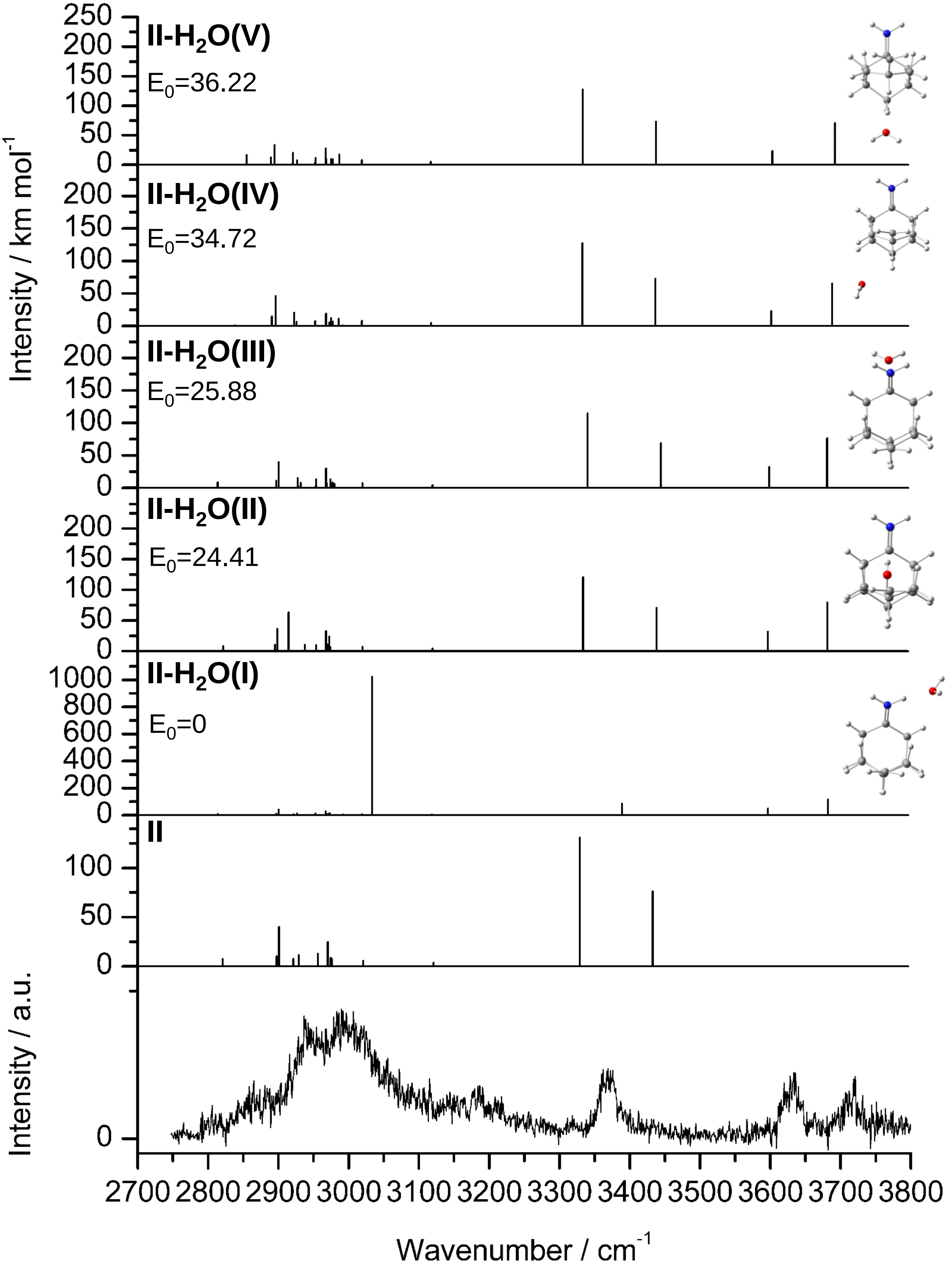
II-H2O(V)
E0=36.22
II-H2O(IV)
E0=34.72
II-H2O(III)
E0=25.88
II-H2O(II)
E0=24.41
II-H2O(I)
E0=0
II
Intensity / km mol-1
Intensity / a.u.
Wavenumber / cm-1
250
200
150
100
50
0
1000
800
600
400
200
2700
2800
2900
3000
3100
3200
3300
3400
3500
3600
3700
3800

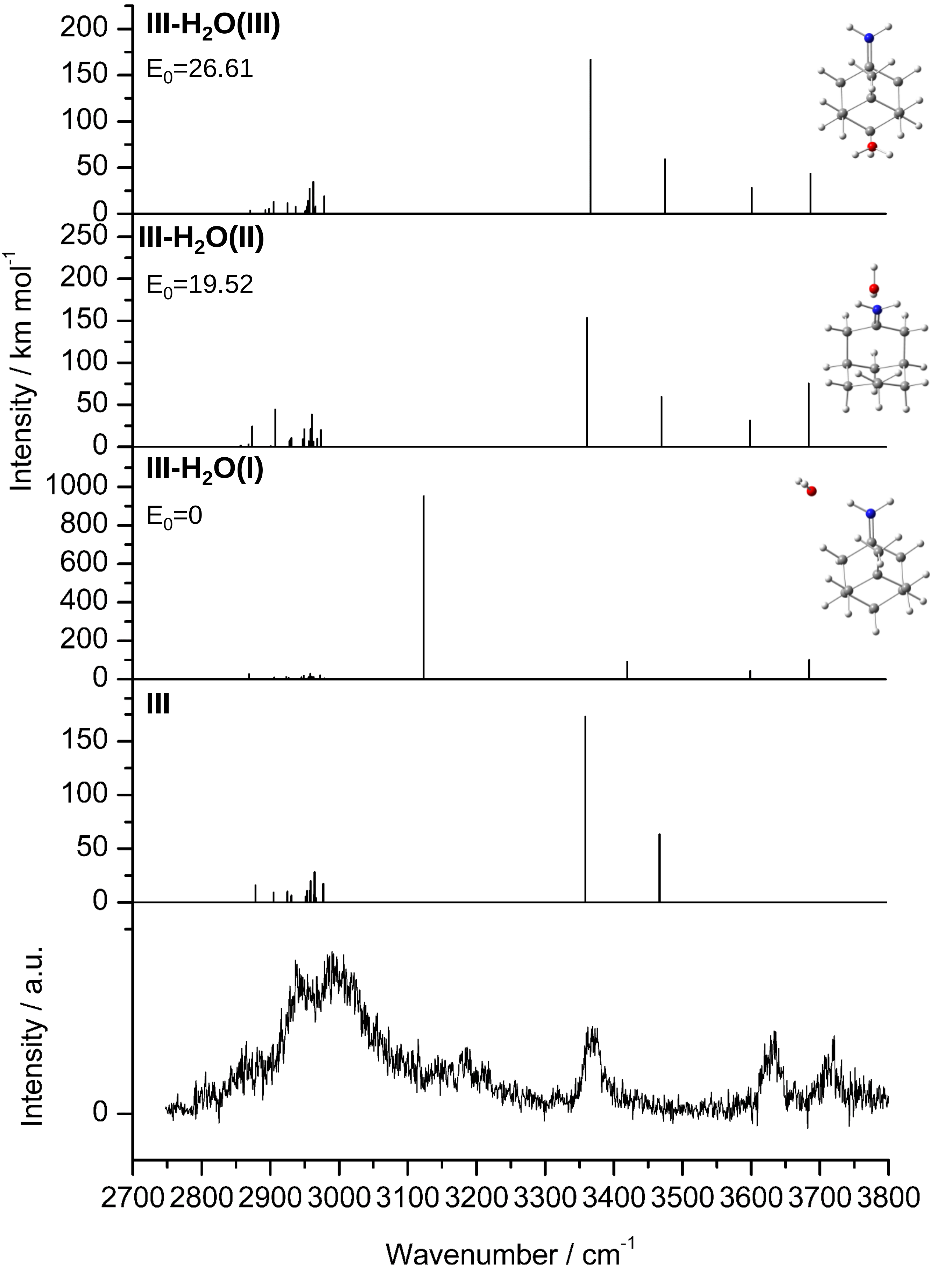
III-H$_2$O(III)
E$_0$=26.61
III-H$_2$O(II)
E$_0$=19.52
III-H$_2$O(I)
E$_0$=0
III
Intensity / km mol^{-1}
Intensity / a.u.
Wavenumber / cm^{-1}
2700 2800 2900 3000 3100 3200 3300 3400 3500 3600 3700 3800

Figure S34

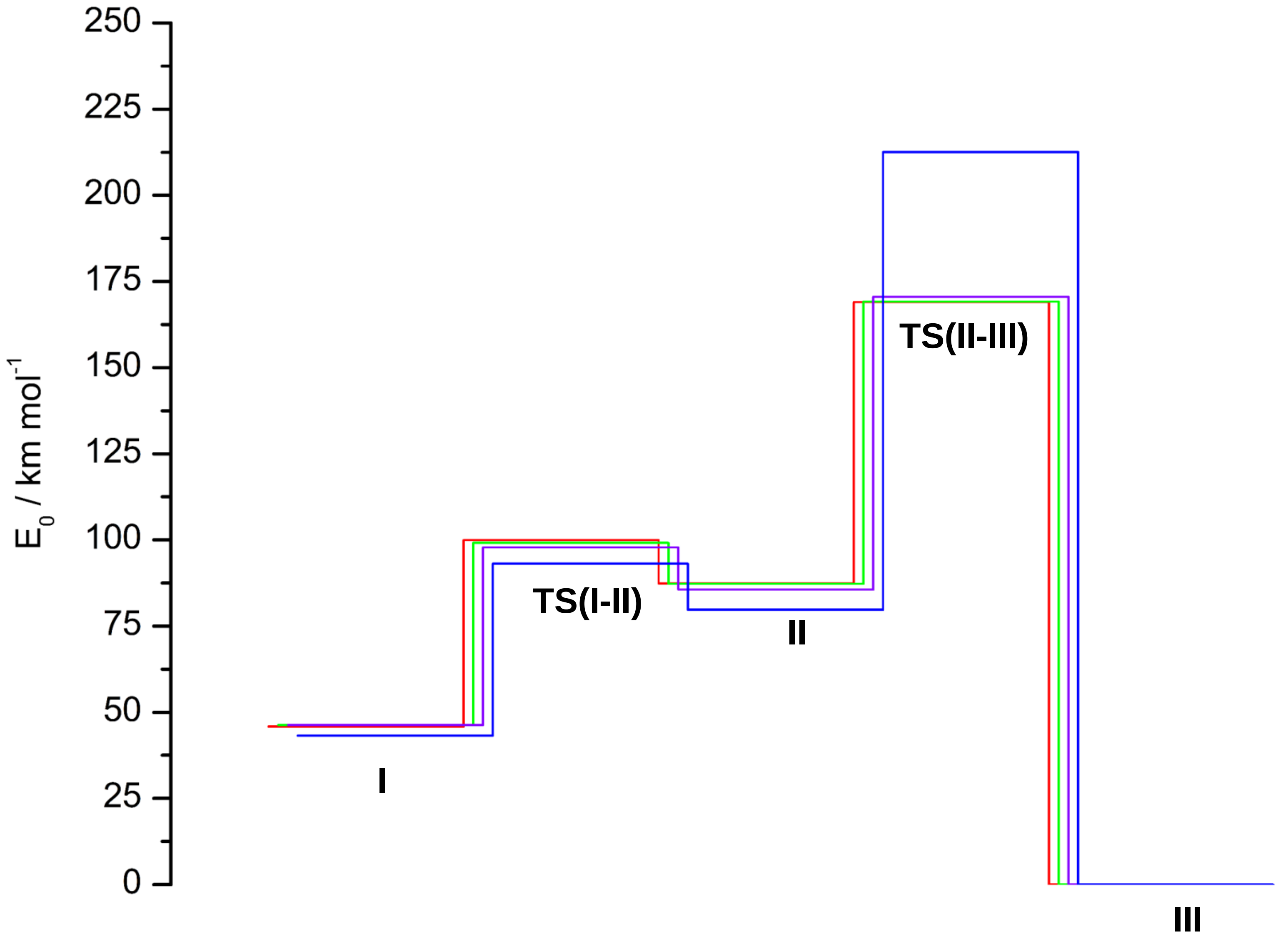

E0 / km mol-1
250
225
200
175
150
125
100
75
50
25
0
I
TS(I-II)
II
TS(II-III)
III

Figure S35

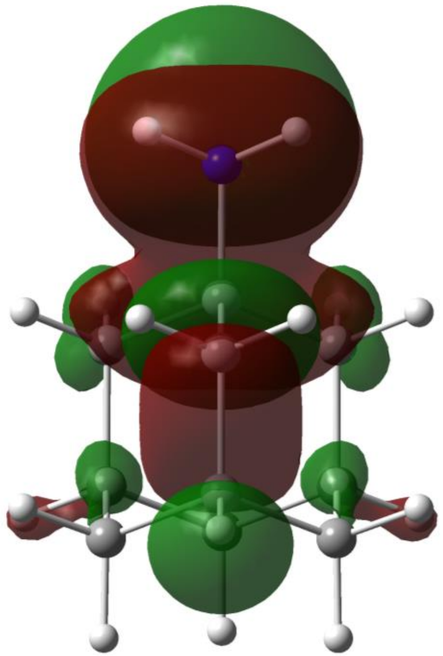

Ama (C_s)

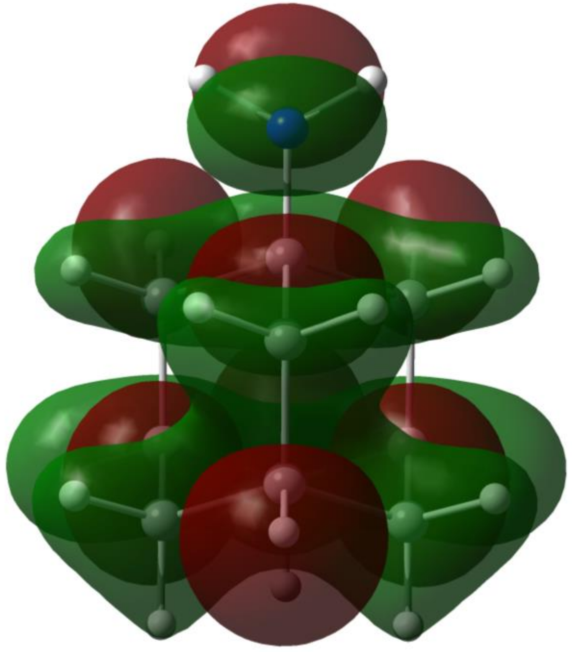

Ama$^+$(**I**) (C_s)

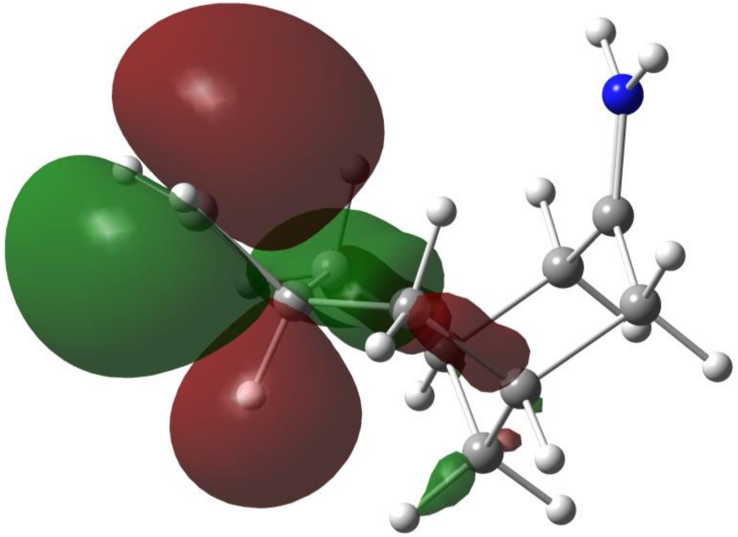

Ama$^+$(**II**) (C_s)

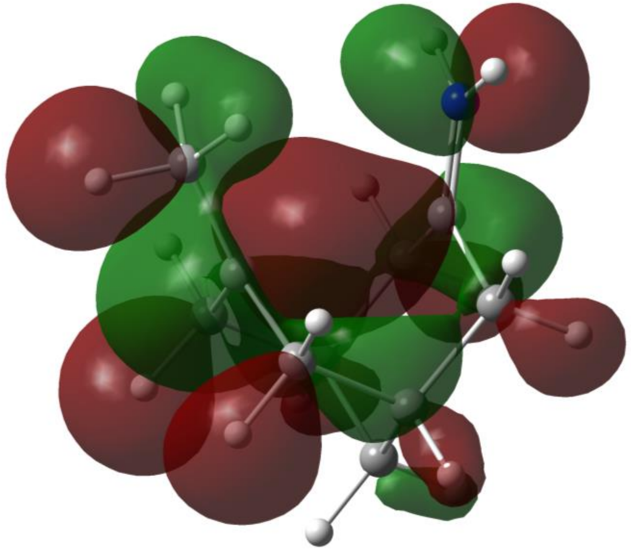

Ama$^+$(**III**) (C_s)

Figure S36

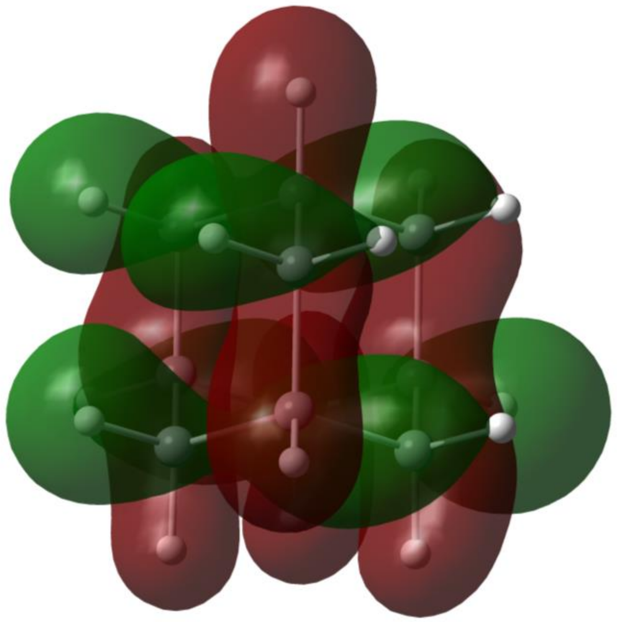

Ada (C_s)

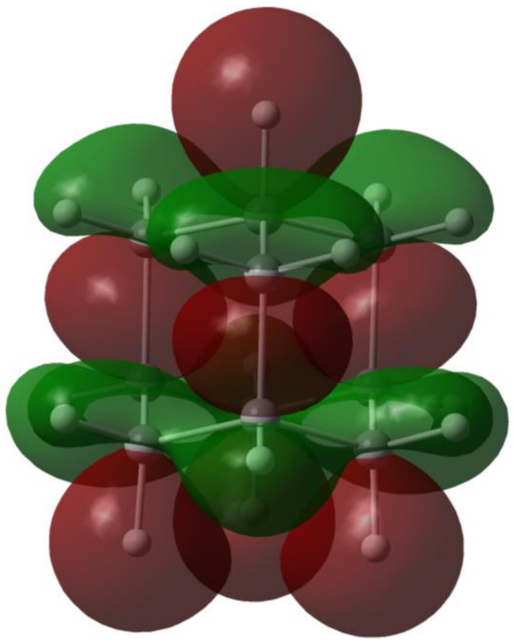

Ada$^+$(**I**) (C_s)

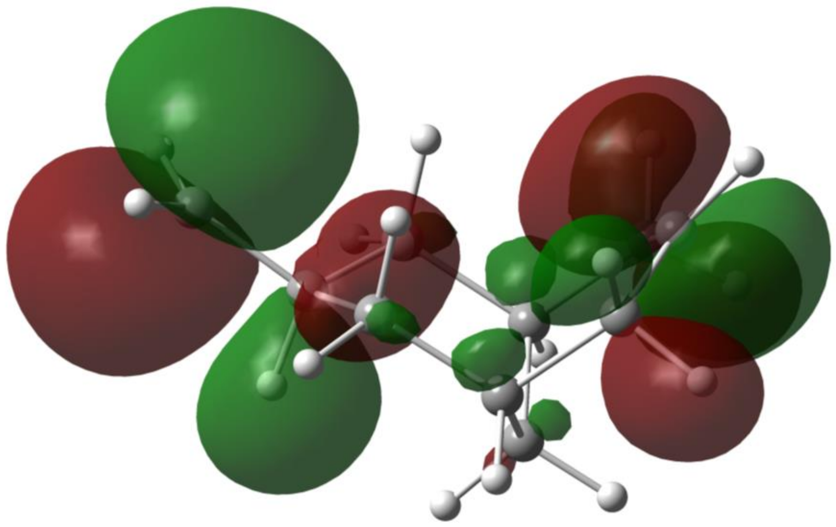

Ada$^+$(**II**) (C_s)

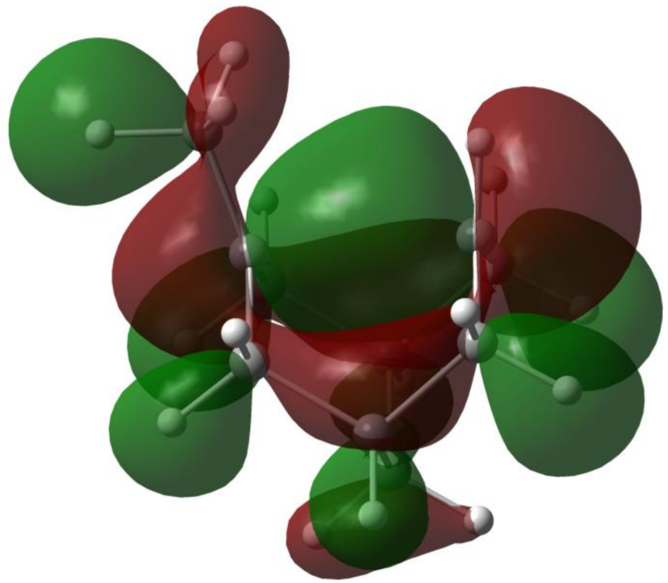

Ada$^+$(**III**) (C_s)

Figure S37

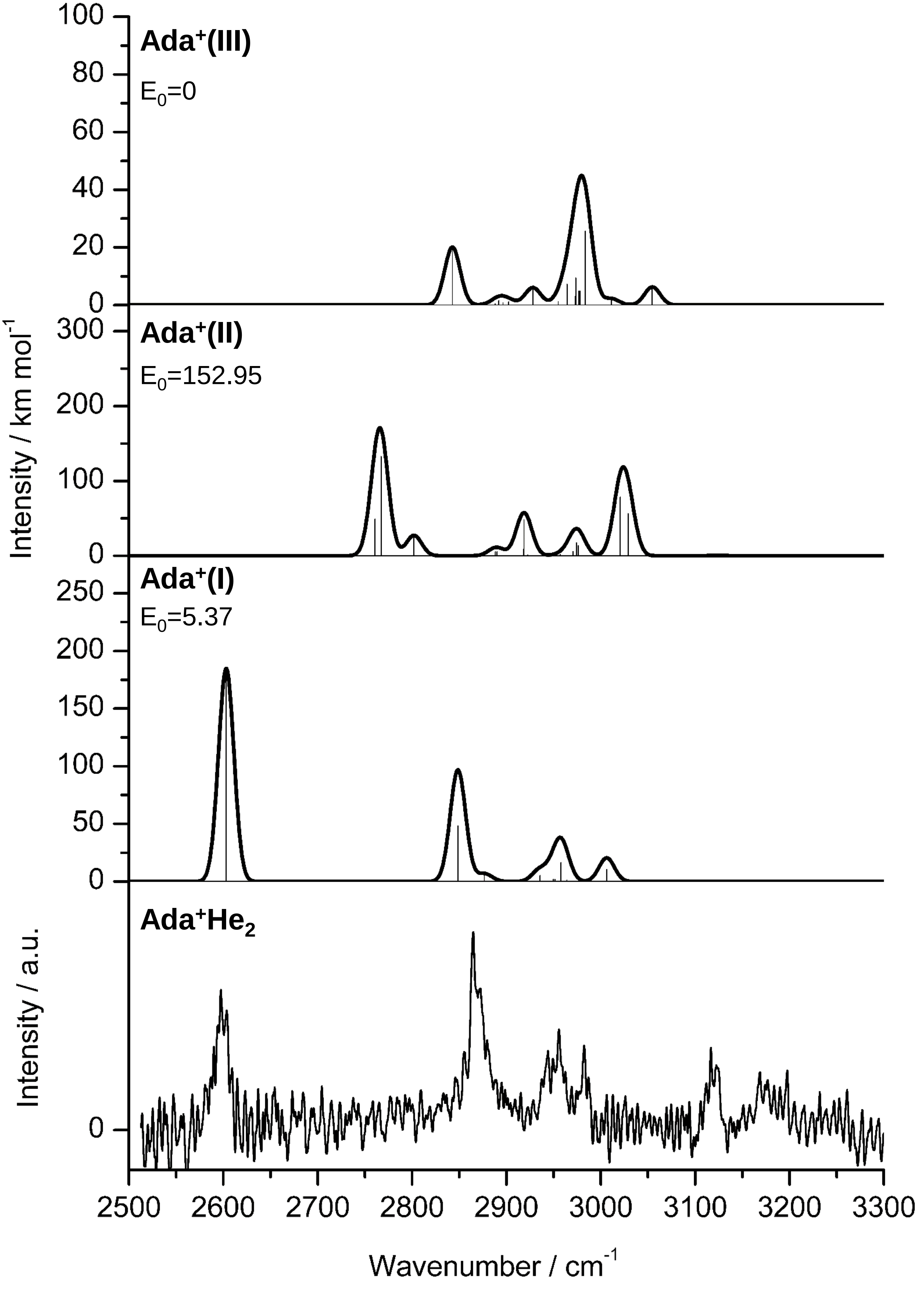

Ada+(III)
E0=0
Ada+(II)
E0=152.95
Ada+(I)
E0=5.37
Ada+He2
Intensity / km mol-1
Intensity / a.u.
Wavenumber / cm-1
100
80
60
40
20
0
300
200
100
0
250
200
150
100
50
0
0
2500
2600
2700
2800
2900
3000
3100
3200
3300

Cartesian coordiantes (Å) and energies (hartree) of relevant structures (B3LYP-D3/cc-pVTZ)

Ama

1	6	0	-1.011128	1.127081	1.255703
2	1	0	-0.514261	1.502017	2.154656
3	1	0	-2.038105	1.501352	1.276200
4	6	0	-1.011128	-0.410783	1.256075
5	1	0	-1.521728	-0.777686	2.149414
6	6	0	-0.287239	1.641427	0.000000
7	1	0	-0.281288	2.733996	0.000000
8	6	0	1.158945	1.116612	0.000000
9	1	0	1.696050	1.483603	0.880296
10	1	0	1.696050	1.483603	-0.880296
11	6	0	0.437377	-0.927013	1.249983
12	1	0	0.460778	-2.018647	1.262920
13	1	0	0.962767	-0.582715	2.146909
14	6	0	1.182128	-0.427579	0.000000
15	6	0	0.437377	-0.927013	-1.249983
16	1	0	0.962767	-0.582715	-2.146909
17	1	0	0.460778	-2.018647	-1.262920
18	6	0	-1.736812	-0.921403	0.000000
19	1	0	-2.774040	-0.575535	0.000000
20	1	0	-1.762177	-2.014271	0.000000
21	6	0	-1.011128	1.127081	-1.255703
22	1	0	-2.038105	1.501352	-1.276200
23	1	0	-0.514261	1.502017	-2.154656
24	6	0	-1.011128	-0.410783	-1.256075
25	1	0	-1.521728	-0.777686	-2.149414
26	7	0	2.535235	-0.993222	0.000000
27	1	0	3.048130	-0.666622	-0.812633
28	1	0	3.048130	-0.666622	0.812633

Sum of electronic and zero-point Energies= -445.999992
Sum of electronic and thermal Energies= -445.991746
Sum of electronic and thermal Enthalpies= -445.990802
Sum of electronic and thermal Free Energies= -446.032017

Ama$^+$(I)

1	6	0	-1.009933	1.119927	1.260815
2	1	0	-0.519111	1.502470	2.157785
3	1	0	-2.034331	1.491912	1.270028
4	6	0	-1.009933	-0.418569	1.258258
5	1	0	-1.512657	-0.782974	2.154281
6	6	0	-0.292961	1.610622	0.000000
7	1	0	-0.245778	2.709641	0.000000
8	6	0	1.169160	1.189049	0.000000
9	1	0	1.708022	1.497285	0.892533
10	1	0	1.708022	1.497285	-0.892533
11	6	0	0.436294	-0.931980	1.280099
12	1	0	0.450942	-2.023690	1.314481
13	1	0	0.966411	-0.567549	2.163360
14	6	0	1.172297	-0.507942	0.000000
15	6	0	0.436294	-0.931980	-1.280099
16	1	0	0.966411	-0.567549	-2.163360
17	1	0	0.450942	-2.023690	-1.314481
18	6	0	-1.725250	-0.938846	0.000000
19	1	0	-2.762343	-0.601654	0.000000
20	1	0	-1.747492	-2.030538	0.000000
21	6	0	-1.009933	1.119927	-1.260815
22	1	0	-2.034331	1.491912	-1.270028
23	1	0	-0.519111	1.502470	-2.157785
24	6	0	-1.009933	-0.418569	-1.258258
25	1	0	-1.512657	-0.782974	-2.154281
26	7	0	2.515855	-0.841720	0.000000
27	1	0	3.044730	-0.885075	-0.862169
28	1	0	3.044730	-0.885075	0.862169

Sum of electronic and zero-point Energies= -445.710234
Sum of electronic and thermal Energies= -445.701526
Sum of electronic and thermal Enthalpies= -445.700582
Sum of electronic and thermal Free Energies= -445.743151

Ama$^+$(II)

1	6	0	0.878102	-0.318597	1.247668
2	6	0	1.635327	-0.783226	0.000000
3	6	0	0.878102	-0.318597	-1.247668
4	6	0	-0.568901	-0.886083	-1.279077
5	6	0	-0.955442	-1.657448	0.000000
6	6	0	-0.568901	-0.886083	1.279077
7	6	0	0.878102	1.245884	1.270499
8	6	0	-2.392771	-2.036083	0.000000
9	6	0	0.309242	1.748203	0.000000
10	6	0	0.878102	1.245884	-1.270499
11	7	0	-0.730363	2.520450	0.000000
12	1	0	1.416275	-0.624513	2.143007
13	1	0	2.653570	-0.389067	0.000000
14	1	0	1.727499	-1.868303	0.000000
15	1	0	1.416275	-0.624513	-2.143007
16	1	0	-1.294078	-0.080902	-1.429269
17	1	0	-0.686147	-1.547069	-2.136958
18	1	0	-0.364623	-2.585563	0.000000
19	1	0	-1.294078	-0.080902	1.429269
20	1	0	-0.686147	-1.547069	2.136958
21	1	0	0.337490	1.629612	2.135471
22	1	0	1.913834	1.590934	1.332043
23	1	0	-2.899004	-2.268577	-0.926112
24	1	0	-2.899004	-2.268577	0.926112
25	1	0	0.337490	1.629612	-2.135471
26	1	0	1.913834	1.590934	-1.332043
27	1	0	-1.153201	2.838847	0.862911
28	1	0	-1.153201	2.838847	-0.862911

Sum of electronic and zero-point Energies= -445.694426
Sum of electronic and thermal Energies= -445.684131
Sum of electronic and thermal Enthalpies= -445.683187
Sum of electronic and thermal Free Energies= -445.730175

Ama$^+$(III)

1	6	0	0.923219	-0.687401	1.262057
2	6	0	1.637589	-1.195743	0.000000
3	6	0	0.923219	-0.687401	-1.262057
4	6	0	-0.547317	-1.154933	-1.263904
5	6	0	-1.207910	-0.715660	0.000000
6	6	0	-0.547317	-1.154933	1.263904
7	6	0	0.923219	0.854630	1.276496
8	6	0	-2.594709	-0.193357	0.000000
9	6	0	0.358830	1.379799	0.000000
10	6	0	0.923219	0.854630	-1.276496
11	7	0	-0.415579	2.442147	0.000000
12	1	0	1.432506	-1.047956	2.154616
13	1	0	2.679262	-0.869158	0.000000
14	1	0	1.650442	-2.286200	0.000000
15	1	0	1.432506	-1.047956	-2.154616
16	1	0	-1.072493	-0.775868	-2.141394
17	1	0	-0.569959	-2.249855	-1.329234
18	1	0	-1.072493	-0.775868	2.141394
19	1	0	-0.569959	-2.249855	1.329234
20	1	0	0.380399	1.243863	2.139094
21	1	0	1.953469	1.219584	1.347374
22	1	0	0.380399	1.243863	-2.139094
23	1	0	1.953469	1.219584	-1.347374
24	1	0	-0.742050	2.856458	0.860499
25	1	0	-0.742050	2.856458	-0.860499
26	1	0	-2.812381	0.398211	-0.890584
27	1	0	-2.812381	0.398211	0.890584
28	1	0	-3.311898	-1.026340	0.000000

Sum of electronic and zero-point Energies= -445.727708
Sum of electronic and thermal Energies= -445.717586
Sum of electronic and thermal Enthalpies= -445.716642
Sum of electronic and thermal Free Energies= -445.762902

Ama$^+$(I)-Ar(I)

1	6	0	-2.618310	0.424296	-1.263667
2	1	0	-2.649048	1.221982	-2.008570
3	1	0	-3.486122	-0.211925	-1.437666
4	6	0	-2.672916	1.009298	0.158431
5	1	0	-3.579686	1.603727	0.271411
6	6	0	-1.335938	-0.400545	-1.405136
7	1	0	-1.256052	-0.797325	-2.427612
8	6	0	-0.100258	0.476714	-1.260835
9	1	0	-0.089732	1.321618	-1.945106
10	1	0	0.832889	-0.076302	-1.328764
11	6	0	-1.465943	1.930387	0.380596
12	1	0	-1.510717	2.380328	1.374970
13	1	0	-1.456210	2.743831	-0.348925
14	6	0	-0.161116	1.125013	0.301888
15	6	0	-0.142642	-0.075422	1.260870
16	1	0	0.780449	-0.645521	1.138958
17	1	0	-0.150809	0.323674	2.277582
18	6	0	-2.653723	-0.128318	1.193144
19	1	0	-3.528571	-0.764941	1.056971
20	1	0	-2.711685	0.278248	2.204846
21	6	0	-1.315777	-1.551630	-0.395297
22	1	0	-2.173798	-2.202409	-0.563076
23	1	0	-0.418584	-2.160350	-0.523276
24	6	0	-1.372241	-0.962699	1.025280
25	1	0	-1.351726	-1.772605	1.754723
26	7	0	0.977390	1.910071	0.381955
27	1	0	1.877292	1.501036	0.602467
28	1	0	0.978863	2.864633	0.044620
29	18	0	3.573428	-0.516074	-0.050326

Sum of electronic and zero-point Energies= -973.272792
Sum of electronic and thermal Energies= -973.261623
Sum of electronic and thermal Enthalpies= -973.260679
Sum of electronic and thermal Free Energies= -973.312063

Ama$^+$(II)-Ar(I)

1	6	0	-0.588167	-0.180326	1.533254
2	6	0	-2.039525	-0.567717	1.234716
3	6	0	-2.231651	-0.659440	-0.282073
4	6	0	-1.901357	0.689495	-0.979866
5	6	0	-1.327712	1.754343	-0.021810
6	6	0	-0.217547	1.182979	0.883828
7	6	0	0.345483	-1.327424	1.021182
8	6	0	-0.869731	2.962986	-0.756036
9	6	0	0.069434	-1.565564	-0.413260
10	6	0	-1.329754	-1.818915	-0.823381
11	7	0	1.006291	-1.436832	-1.297280
12	1	0	-0.430968	-0.133243	2.609338
13	1	0	-2.288533	-1.517903	1.711279
14	1	0	-2.719156	0.172491	1.654578
15	1	0	-3.254638	-0.954098	-0.509104
16	1	0	-1.186762	0.536258	-1.793965
17	1	0	-2.798352	1.089039	-1.451452
18	1	0	-2.151701	2.057719	0.641171
19	1	0	0.698576	1.089533	0.294161
20	1	0	0.009878	1.911254	1.661366
21	1	0	1.394292	-1.093016	1.197338
22	1	0	0.100382	-2.243032	1.565814
23	1	0	-1.382035	3.287985	-1.650499
24	1	0	-0.150376	3.636010	-0.311482
25	1	0	-1.423265	-1.920966	-1.904369
26	1	0	-1.653016	-2.754527	-0.359699
27	1	0	1.956388	-1.206830	-1.031444
28	1	0	0.820067	-1.554853	-2.285142
29	18	0	3.775464	0.379640	0.036874

Sum of electronic and zero-point Energies= -973.257189
Sum of electronic and thermal Energies= -973.244418
Sum of electronic and thermal Enthalpies= -973.243474
Sum of electronic and thermal Free Energies= -973.299172

Ama$^+$(III)-Ar(I)

1	6	0	1.270304	-0.691280	1.147344
2	6	0	2.729542	-0.380196	0.779887
3	6	0	2.797434	0.349721	-0.571106
4	6	0	2.152406	-0.517202	-1.671835
5	6	0	0.759778	-0.873392	-1.270608
6	6	0	0.618583	-1.556167	0.048883
7	6	0	0.467373	0.621406	1.248555
8	6	0	-0.344467	-0.881118	-2.258727
9	6	0	0.622721	1.415004	-0.005162
10	6	0	2.008188	1.672549	-0.492347
11	7	0	-0.397446	2.068875	-0.513484
12	1	0	1.224440	-1.205500	2.106314
13	1	0	3.192203	0.227464	1.559950
14	1	0	3.300269	-1.307829	0.724484
15	1	0	3.832330	0.572469	-0.827072
16	1	0	2.171682	-0.001090	-2.632471
17	1	0	2.745669	-1.432394	-1.792202
18	1	0	-0.427218	-1.756411	0.284010
19	1	0	1.127769	-2.527516	0.016052
20	1	0	-0.583082	0.430667	1.468423
21	1	0	0.867046	1.232340	2.065012
22	1	0	2.001034	2.187599	-1.454111
23	1	0	2.485675	2.340426	0.232526
24	1	0	-1.332688	1.946599	-0.153493
25	1	0	-0.289792	2.668799	-1.317927
26	1	0	-0.242868	-0.083906	-2.996741
27	1	0	-1.322988	-0.811744	-1.782500
28	1	0	-0.339405	-1.825143	-2.821799
29	18	0	-3.290907	0.066219	0.262241

Sum of electronic and zero-point Energies= -973.290429
Sum of electronic and thermal Energies= -973.277839
Sum of electronic and thermal Enthalpies= -973.276895
Sum of electronic and thermal Free Energies= -973.331272

Ama$^+$(I)-Ar$_2$(I)

1	6	0	-1.120596	-2.011717	1.260615
2	1	0	-1.458896	-1.489087	2.157595
3	1	0	-1.580719	-2.999804	1.271339
4	6	0	0.412599	-2.144387	1.258114
5	1	0	0.732204	-2.676707	2.154174
6	6	0	-1.549900	-1.255355	0.000000
7	1	0	-2.640304	-1.114806	0.000000
8	6	0	-1.001270	0.165671	0.000000
9	1	0	-1.262928	0.728541	0.892196
10	1	0	-1.262928	0.728541	-0.892196
11	6	0	1.046136	-0.747065	1.279410
12	1	0	2.135144	-0.824855	1.312001
13	1	0	0.729449	-0.187990	2.162268
14	6	0	0.682023	0.020970	0.000000
15	6	0	1.046136	-0.747065	-1.279410
16	1	0	0.729449	-0.187990	-2.162268
17	1	0	2.135144	-0.824855	-1.312001
18	6	0	0.869619	-2.901494	0.000000
19	1	0	0.446544	-3.906711	0.000000
20	1	0	1.955594	-3.015593	0.000000
21	6	0	-1.120596	-2.011717	-1.260615
22	1	0	-1.580719	-2.999804	-1.271339
23	1	0	-1.458896	-1.489087	-2.157595
24	6	0	0.412599	-2.144387	-1.258114
25	1	0	0.732204	-2.676707	-2.154174
26	7	0	1.128411	1.333026	0.000000
27	1	0	1.196058	1.858000	-0.863426
28	1	0	1.196058	1.858000	0.863426
29	18	0	-0.186162	2.570806	-3.076750
30	18	0	-0.186162	2.570806	3.076750

Sum of electronic and zero-point Energies= -1500.835202
Sum of electronic and thermal Energies= -1500.821524
Sum of electronic and thermal Enthalpies= -1500.820580
Sum of electronic and thermal Free Energies= -1500.880785

Ama$^+$(II)-Ar$_2$(I)

1	6	0	1.909246	0.055591	1.247714
2	6	0	2.796294	0.096320	0.000000
3	6	0	1.909246	0.055591	-1.247714
4	6	0	1.029530	-1.225330	-1.280079
5	6	0	1.137620	-2.080126	0.000000
6	6	0	1.029530	-1.225330	1.280079
7	6	0	1.029530	1.350349	1.270078
8	6	0	0.153515	-3.193983	0.000000
9	6	0	0.273531	1.432152	0.000000
10	6	0	1.029530	1.350349	-1.270078
11	7	0	-1.020333	1.446199	0.000000
12	1	0	2.526234	0.104631	2.143194
13	1	0	3.417141	0.994547	0.000000
14	1	0	3.483194	-0.749047	0.000000
15	1	0	2.526234	0.104631	-2.143194
16	1	0	-0.020904	-0.963861	-1.434664
17	1	0	1.305673	-1.839731	-2.136282
18	1	0	2.144594	-2.523306	0.000000
19	1	0	-0.020904	-0.963861	1.434664
20	1	0	1.305673	-1.839731	2.136282
21	1	0	0.368645	1.364212	2.135445
22	1	0	1.690992	2.218552	1.326170
23	1	0	-0.137380	-3.669176	-0.926081
24	1	0	-0.137380	-3.669176	0.926081
25	1	0	0.368645	1.364212	-2.135445
26	1	0	1.690992	2.218552	-1.326170
27	1	0	-1.548189	1.442392	0.864427
28	1	0	-1.548189	1.442392	-0.864427
29	18	0	-2.334949	0.420747	-3.159762
30	18	0	-2.334949	0.420747	3.159762

Sum of electronic and zero-point Energies= -1500.820086
Sum of electronic and thermal Energies= -1500.804690
Sum of electronic and thermal Enthalpies= -1500.803746
Sum of electronic and thermal Free Energies= -1500.869210

Ama$^+$(III)-Ar$_2$(I)

1	6	0	-1.261803	2.094840	-0.170315
2	6	0	-0.000058	2.959174	-0.321333
3	6	0	1.261749	2.094893	-0.170373
4	6	0	1.264299	1.398186	1.206048
5	6	0	0.000029	0.622761	1.372350
6	6	0	-1.264327	1.398047	1.206039
7	6	0	-1.275087	0.994548	-1.251909
8	6	0	0.000092	-0.715018	2.009856
9	6	0	-0.000007	0.222778	-1.210353
10	6	0	1.275049	0.994580	-1.251900
11	7	0	-0.000005	-1.079274	-1.382664
12	1	0	-2.154672	2.708435	-0.282024
13	1	0	-0.000103	3.459107	-1.291699
14	1	0	-0.000049	3.742634	0.437202
15	1	0	2.154574	2.708546	-0.282092
16	1	0	2.141171	0.758889	1.314202
17	1	0	1.331714	2.164185	1.988800
18	1	0	-2.141125	0.758627	1.314088
19	1	0	-1.331888	2.163945	1.988880
20	1	0	-2.138291	0.338141	-1.141334
21	1	0	-1.339247	1.456773	-2.242658
22	1	0	2.138260	0.338181	-1.141302
23	1	0	1.339255	1.456738	-2.242681
24	1	0	-0.861653	-1.604480	-1.411272
25	1	0	0.861645	-1.604474	-1.411281
26	1	0	0.890209	-1.291493	1.755353
27	1	0	-0.889873	-1.291647	1.755170
28	1	0	-0.000037	-0.611646	3.104099
29	18	0	-3.138537	-2.235693	-0.028913
30	18	0	3.138566	-2.235655	-0.028947

Sum of electronic and zero-point Energies= -1500.853394
Sum of electronic and thermal Energies= -1500.838154
Sum of electronic and thermal Enthalpies= -1500.837210
Sum of electronic and thermal Free Energies= -1500.901023

Ama$^+$(I)-Ar$_3$(I)

1	6	0	2.599349	-1.273889	-0.702379
2	1	0	2.260287	-2.177033	-1.213784
3	1	0	3.689244	-1.286256	-0.715440
4	6	0	2.090782	-1.252399	0.750129
5	1	0	2.444229	-2.142419	1.271158
6	6	0	2.087899	-0.021774	-1.421211
7	1	0	2.406870	-0.035824	-2.472817
8	6	0	0.565735	-0.020641	-1.499517
9	1	0	0.159570	-0.917649	-1.960146
10	1	0	0.161981	0.865718	-1.982292
11	6	0	0.557307	-1.270916	0.756522
12	1	0	0.180893	-1.286701	1.781247
13	1	0	0.175222	-2.160081	0.251005
14	6	0	0.009289	0.000469	0.090618
15	6	0	0.561016	1.286372	0.724122
16	1	0	0.181363	2.163838	0.196681
17	1	0	0.184791	1.329215	1.748162
18	6	0	2.595958	0.013938	1.461655
19	1	0	3.686171	0.012600	1.488947
20	1	0	2.252906	0.027772	2.498345
21	6	0	2.602693	1.246819	-0.734751
22	1	0	3.692621	1.256140	-0.748358
23	1	0	2.265735	2.137602	-1.268738
24	6	0	2.094450	1.263285	0.717908
25	1	0	2.450547	2.165085	1.216355
26	7	0	-1.371244	0.000499	-0.038173
27	1	0	-1.879552	0.862939	-0.190347
28	1	0	-1.880860	-0.864260	-0.171645
29	18	0	-1.965832	3.115592	-1.672923
30	18	0	-1.980431	-3.151190	-1.605183
31	18	0	-2.021525	0.047722	3.260345

Sum of electronic and zero-point Energies= -2028.397749
Sum of electronic and thermal Energies= -2028.381383
Sum of electronic and thermal Enthalpies= -2028.380439
Sum of electronic and thermal Free Energies= -2028.450327

Ama$^+$(II)-Ar$_3$(I)

1	6	0	2.597225	1.250208	0.267569
2	6	0	2.227653	2.727521	0.098633
3	6	0	0.715282	2.843244	-0.115301
4	6	0	0.256092	2.050745	-1.372014
5	6	0	1.383850	1.222349	-2.020716
6	6	0	2.181131	0.414392	-0.975640
7	6	0	1.910752	0.715247	1.568108
8	6	0	0.871470	0.355048	-3.113587
9	6	0	0.453851	0.956803	1.468014
10	6	0	-0.007028	2.333086	1.175861
11	7	0	-0.384168	-0.027216	1.508105
12	1	0	3.667831	1.153092	0.438727
13	1	0	2.536101	3.301615	0.974598
14	1	0	2.760929	3.153470	-0.750108
15	1	0	0.434037	3.890187	-0.214184
16	1	0	-0.567567	1.377530	-1.119697
17	1	0	-0.145726	2.739479	-2.114275
18	1	0	2.087371	1.940822	-2.467553
19	1	0	1.581413	-0.448286	-0.673968
20	1	0	3.067852	-0.000077	-1.454084
21	1	0	2.135217	-0.338145	1.729330
22	1	0	2.290204	1.284030	2.420753
23	1	0	0.030051	0.668038	-3.715194
24	1	0	1.437259	-0.504890	-3.442753
25	1	0	-1.089436	2.381808	1.067797
26	1	0	0.285798	2.971959	2.013017
27	1	0	-0.072504	-0.981771	1.639066
28	1	0	-1.377515	0.118169	1.374589
29	18	0	-3.642268	0.996212	0.395495
30	18	0	1.325186	-3.138290	0.961903
31	18	0	-1.491090	-1.730832	-1.173150

Sum of electronic and zero-point Energies= -2028.382971
Sum of electronic and thermal Energies= -2028.365008
Sum of electronic and thermal Enthalpies= -2028.364064
Sum of electronic and thermal Free Energies= -2028.437578

Ama$^+$(III)-Ar$_3$(I)

1	6	0	0.176983	2.618194	-1.261808
2	6	0	0.329074	3.482283	0.000000
3	6	0	0.176983	2.618194	1.261808
4	6	0	-1.200712	1.923633	1.264281
5	6	0	-1.367006	1.148070	0.000000
6	6	0	-1.200712	1.923633	-1.264281
7	6	0	1.256881	1.515917	-1.274573
8	6	0	-2.000127	-0.191962	0.000000
9	6	0	1.209827	0.743566	0.000000
10	6	0	1.256881	1.515917	1.274573
11	7	0	1.367592	-0.559573	0.000000
12	1	0	0.289895	3.231600	-2.154678
13	1	0	1.300142	3.980857	0.000000
14	1	0	-0.428423	4.266772	0.000000
15	1	0	0.289895	3.231600	2.154678
16	1	0	-1.309867	1.284663	2.141194
17	1	0	-1.982173	2.690934	1.331346
18	1	0	-1.309867	1.284663	-2.141194
19	1	0	-1.982173	2.690934	-1.331346
20	1	0	1.146627	0.860581	-2.138391
21	1	0	2.248465	1.976430	-1.336185
22	1	0	1.146627	0.860581	2.138391
23	1	0	2.248465	1.976430	1.336185
24	1	0	1.379543	-1.088641	-0.859611
25	1	0	1.379543	-1.088641	0.859611
26	1	0	-1.742586	-0.768027	0.889352
27	1	0	-1.742586	-0.768027	-0.889352
28	1	0	-3.094698	-0.092880	0.000000
29	18	0	0.003693	-1.621885	-3.202821
30	18	0	0.034925	-3.667202	0.000000
31	18	0	0.003693	-1.621885	3.202821

Sum of electronic and zero-point Energies= -2028.415728
Sum of electronic and thermal Energies= -2028.397946
Sum of electronic and thermal Enthalpies= -2028.397002
Sum of electronic and thermal Free Energies= -2028.468924

Ama$^+$(I)-N_2(I)

1	6	0	-2.759462	0.952758	-0.627116
2	1	0	-2.828494	1.985454	-0.974862
3	1	0	-3.730569	0.489827	-0.803187
4	6	0	-2.419413	0.908414	0.872427
5	1	0	-3.179677	1.451833	1.433758
6	6	0	-1.677512	0.192934	-1.400214
7	1	0	-1.879369	0.246351	-2.479072
8	6	0	-0.318698	0.867741	-1.247330
9	1	0	-0.327995	1.915233	-1.539146
10	1	0	0.479679	0.341752	-1.765174
11	6	0	-1.066692	1.591885	1.111363
12	1	0	-0.827138	1.596525	2.176957
13	1	0	-1.090994	2.631186	0.774651
14	6	0	0.052339	0.823015	0.391973
15	6	0	0.093637	-0.664103	0.782601
16	1	0	0.871332	-1.183870	0.218663
17	1	0	0.363788	-0.715942	1.839521
18	6	0	-2.351418	-0.552600	1.349544
19	1	0	-3.321206	-1.032014	1.211628
20	1	0	-2.127066	-0.592882	2.417441
21	6	0	-1.617730	-1.270096	-0.950322
22	1	0	-2.580173	-1.750021	-1.128066
23	1	0	-0.872011	-1.820689	-1.527421
24	6	0	-1.278106	-1.310505	0.549790
25	1	0	-1.225134	-2.347590	0.881930
26	7	0	1.288941	1.444455	0.475245
27	1	0	2.150730	0.931724	0.310043
28	1	0	1.357590	2.452478	0.544385
29	7	0	3.965439	-0.187609	-0.047857
30	7	0	4.887378	-0.751638	-0.193190

Sum of electronic and zero-point Energies= -555.278190
Sum of electronic and thermal Energies= -555.265703
Sum of electronic and thermal Enthalpies= -555.264759
Sum of electronic and thermal Free Energies= -555.319245

Ama$^+$(II)-N_2(I)

1	6	0	2.264647	0.642075	0.515244
2	6	0	2.147327	0.948836	-0.981102
3	6	0	0.791875	0.449280	-1.489947
4	6	0	0.627927	-1.080175	-1.263824
5	6	0	1.782282	-1.710539	-0.456872
6	6	0	2.133692	-0.881561	0.795108
7	6	0	1.164635	1.455662	1.273035
8	6	0	1.502518	-3.130094	-0.115983
9	6	0	-0.159924	1.128175	0.698007
10	6	0	-0.335858	1.256015	-0.767218
11	7	0	-1.102265	0.637446	1.435455
12	1	0	3.216794	1.008393	0.894823
13	1	0	2.253632	2.020413	-1.161720
14	1	0	2.955281	0.465161	-1.528265
15	1	0	0.686441	0.680368	-2.548509
16	1	0	-0.312365	-1.291898	-0.746400
17	1	0	0.553328	-1.588075	-2.224716
18	1	0	2.669268	-1.691996	-1.107888
19	1	0	1.371116	-1.066845	1.557343
20	1	0	3.064396	-1.259796	1.216437
21	1	0	1.198322	1.262311	2.345080
22	1	0	1.347005	2.521295	1.110473
23	1	0	0.930203	-3.753316	-0.788413
24	1	0	2.019333	-3.610286	0.702753
25	1	0	-1.324384	0.926421	-1.084027
26	1	0	-0.226920	2.312804	-1.025813
27	1	0	-0.973527	0.510189	2.431302
28	1	0	-2.000922	0.367110	1.045214
29	7	0	-3.866831	-0.249105	0.186473
30	7	0	-4.823044	-0.567094	-0.230297

Sum of electronic and zero-point Energies= -555.263239
Sum of electronic and thermal Energies= -555.249048
Sum of electronic and thermal Enthalpies= -555.248104
Sum of electronic and thermal Free Energies= -555.307242

Ama$^+$(III)-N_2(I)

1	6	0	1.293652	-0.481504	1.244499
2	6	0	2.663400	0.122454	0.898063
3	6	0	2.655939	0.679308	-0.534259
4	6	0	2.305713	-0.440831	-1.535777
5	6	0	1.009946	-1.069677	-1.144219
6	6	0	0.943212	-1.605090	0.247329
7	6	0	0.200002	0.600856	1.134346
8	6	0	-0.002259	-1.444386	-2.159729
9	6	0	0.259619	1.258151	-0.203830
10	6	0	1.580620	1.778829	-0.659536
11	7	0	-0.843125	1.576923	-0.839270
12	1	0	1.300659	-0.871416	2.261510
13	1	0	2.917296	0.912142	1.607750
14	1	0	3.435015	-0.642735	0.989879
15	1	0	3.627173	1.108581	-0.776489
16	1	0	2.269192	-0.051652	-2.553965
17	1	0	3.103070	-1.194151	-1.513129
18	1	0	-0.039982	-2.023059	0.467072
19	1	0	1.668001	-2.420197	0.365234
20	1	0	-0.790409	0.187363	1.326334
21	1	0	0.379468	1.380670	1.882118
22	1	0	1.523538	2.164793	-1.678334
23	1	0	1.840308	2.618380	-0.006044
24	1	0	-1.750040	1.270362	-0.511314
25	1	0	-0.814759	2.086907	-1.709753
26	1	0	-0.043305	-0.729473	-2.982983
27	1	0	-0.997364	-1.553134	-1.725404
28	1	0	0.248729	-2.416661	-2.606625
29	7	0	-3.616021	0.262762	0.126455
30	7	0	-4.552871	-0.214217	0.416597

Sum of electronic and zero-point Energies= -555.295838
Sum of electronic and thermal Energies= -555.281687
Sum of electronic and thermal Enthalpies= -555.280743
Sum of electronic and thermal Free Energies= -555.339761

Ama$^+$(I)-H_2O(I)

1	6	0	-1.083868	1.829552	0.168291
2	1	0	-0.319033	2.605809	0.097682
3	1	0	-2.044041	2.333771	0.280440
4	6	0	-0.821113	0.921080	1.380933
5	1	0	-0.801910	1.523433	2.289745
6	6	0	-1.097310	0.970344	-1.099992
7	1	0	-1.246627	1.604856	-1.982356
8	6	0	0.265806	0.314155	-1.324329
9	1	0	1.077715	1.036713	-1.364527
10	1	0	0.284796	-0.313197	-2.213005
11	6	0	0.547564	0.244700	1.225595
12	1	0	0.761022	-0.385201	2.091590
13	1	0	1.347517	0.982764	1.146143
14	6	0	0.543721	-0.667467	-0.017562
15	6	0	-0.589678	-1.702540	0.024610
16	1	0	-0.582356	-2.309335	-0.884656
17	1	0	-0.403046	-2.370287	0.868380
18	6	0	-1.921714	-0.148881	1.478719
19	1	0	-2.891204	0.330572	1.619868
20	1	0	-1.753573	-0.786451	2.349304
21	6	0	-2.202003	-0.088651	-1.022017
22	1	0	-3.171819	0.399431	-0.921584
23	1	0	-2.233688	-0.679475	-1.939884
24	6	0	-1.941767	-0.994244	0.193857
25	1	0	-2.721444	-1.753354	0.257939
26	7	0	1.792739	-1.222783	-0.278404
27	1	0	1.864038	-2.079978	-0.814166
28	1	0	2.658096	-0.689982	-0.093929
29	8	0	3.964915	0.456178	0.228712
30	1	0	4.567800	0.810224	-0.433876
31	1	0	4.444904	0.489906	1.063260

Sum of electronic and zero-point Energies= -522.172210
Sum of electronic and thermal Energies= -522.160107
Sum of electronic and thermal Enthalpies= -522.159163
Sum of electronic and thermal Free Energies= -522.210494

Ama+(II)-H_2O(I)

1	6	0	-0.307557	-0.257828	1.541455
2	6	0	-1.386780	-1.253122	1.102851
3	6	0	-1.418066	-1.314678	-0.427967
4	6	0	-1.720601	0.079570	-1.045735
5	6	0	-1.771912	1.216873	-0.005235
6	6	0	-0.578767	1.162818	0.970643
7	6	0	1.082705	-0.795823	1.077407
8	6	0	-1.893515	2.549503	-0.652445
9	6	0	1.045935	-1.030125	-0.387588
10	6	0	-0.047257	-1.873720	-0.927711
11	7	0	1.876356	-0.425055	-1.169009
12	1	0	-0.263980	-0.216327	2.628456
13	1	0	-1.185543	-2.241950	1.520128
14	1	0	-2.359741	-0.950208	1.487347
15	1	0	-2.168647	-2.032975	-0.753269
16	1	0	-0.969425	0.333565	-1.799287
17	1	0	-2.670368	0.048350	-1.578643
18	1	0	-2.679261	1.054756	0.595975
19	1	0	0.308955	1.539365	0.454426
20	1	0	-0.761850	1.860425	1.787385
21	1	0	1.886151	-0.113263	1.347571
22	1	0	1.263095	-1.758549	1.563524
23	1	0	-2.419162	2.659294	-1.590459
24	1	0	-1.633812	3.447735	-0.110086
25	1	0	-0.015103	-1.925337	-2.016121
26	1	0	0.080680	-2.885138	-0.532891
27	1	0	2.603738	0.207454	-0.800295
28	1	0	1.824083	-0.556145	-2.171083
29	8	0	3.712902	1.285571	0.050349
30	1	0	3.670154	2.246983	0.007631
31	1	0	4.627218	1.071979	0.265910

Sum of electronic and zero-point Energies= -522.158290
Sum of electronic and thermal Energies= -522.144486
Sum of electronic and thermal Enthalpies= -522.143542
Sum of electronic and thermal Free Energies= -522.199137

Ama+(III)-H_2O(I)

1	6	0	2.018460	0.446014	0.590343
2	6	0	1.871503	1.284713	-0.689389
3	6	0	0.564084	0.928568	-1.412306
4	6	0	0.549035	-0.572401	-1.768610
5	6	0	0.776754	-1.383513	-0.536513
6	6	0	2.008148	-1.056819	0.241340
7	6	0	0.832502	0.727732	1.536515
8	6	0	0.022795	-2.637870	-0.296851
9	6	0	-0.463297	0.532755	0.823823
10	6	0	-0.640293	1.215406	-0.491824
11	7	0	-1.480922	-0.037082	1.413168
12	1	0	2.943722	0.703328	1.104614
13	1	0	1.889641	2.348753	-0.445493
14	1	0	2.719809	1.097567	-1.348783
15	1	0	0.461276	1.527156	-2.316758
16	1	0	-0.390180	-0.842071	-2.253270
17	1	0	1.347880	-0.768246	-2.495554
18	1	0	2.079308	-1.662927	1.145707
19	1	0	2.898571	-1.282206	-0.359671
20	1	0	0.884790	0.109519	2.434161
21	1	0	0.866696	1.774011	1.858227
22	1	0	-1.582390	0.924145	-0.954535
23	1	0	-0.689799	2.290325	-0.286653
24	1	0	-1.378527	-0.444607	2.331050
25	1	0	-2.388743	-0.122574	0.944379
26	1	0	-1.013919	-2.559250	-0.629788
27	1	0	0.040083	-2.933318	0.753287
28	1	0	0.466536	-3.469609	-0.861916
29	8	0	-3.844379	-0.141070	-0.142872
30	1	0	-4.533625	0.530208	-0.095204
31	1	0	-4.278934	-0.934571	-0.472922

Sum of electronic and zero-point Energies= -522.188669
Sum of electronic and thermal Energies= -522.174944
Sum of electronic and thermal Enthalpies= -522.173999
Sum of electronic and thermal Free Energies= -522.229251

Ada

1	6	0	0.000000	0.000000	1.774934
2	1	0	0.621527	0.621527	2.425680
3	1	0	-0.621527	-0.621527	2.425680
4	6	0	0.889683	-0.889683	0.889683
5	1	0	1.520547	-1.520547	1.520547
6	6	0	-0.889683	0.889683	0.889683
7	1	0	-1.520547	1.520547	1.520547
8	6	0	0.000000	1.774934	0.000000
9	1	0	0.621527	2.425680	0.621527
10	1	0	-0.621527	2.425680	-0.621527
11	6	0	1.774934	0.000000	0.000000
12	1	0	2.425680	-0.621527	-0.621527
13	1	0	2.425680	0.621527	0.621527
14	6	0	0.889683	0.889683	-0.889683
15	1	0	1.520547	1.520547	-1.520547
16	6	0	0.000000	0.000000	-1.774934
17	1	0	-0.621527	0.621527	-2.425680
18	1	0	0.621527	-0.621527	-2.425680
19	6	0	0.000000	-1.774934	0.000000
20	1	0	-0.621527	-2.425680	0.621527
21	1	0	0.621527	-2.425680	-0.621527
22	6	0	-1.774934	0.000000	0.000000
23	1	0	-2.425680	-0.621527	0.621527
24	1	0	-2.425680	0.621527	-0.621527
25	6	0	-0.889683	-0.889683	-0.889683
26	1	0	-1.520547	-1.520547	-1.520547

Sum of electronic and zero-point Energies= -390.638558
Sum of electronic and thermal Energies= -390.631860
Sum of electronic and thermal Enthalpies= -390.630915
Sum of electronic and thermal Free Energies= -390.666803

Ada^{+}(I)

1	6	0	-1.264306	0.729947	-1.000887
2	1	0	-1.317997	0.760946	-2.097682
3	1	0	-2.157799	1.245806	-0.647152
4	6	0	0.000000	1.449632	-0.553782
5	1	0	0.000000	2.497787	-0.854160
6	6	0	-1.255418	-0.724816	-0.553782
7	1	0	-2.163147	-1.248893	-0.854160
8	6	0	0.000000	-1.459894	-1.000887
9	1	0	0.000000	-1.521892	-2.097682
10	1	0	0.000000	-2.491611	-0.647152
11	6	0	1.264306	0.729947	-1.000887
12	1	0	2.157799	1.245806	-0.647152
13	1	0	1.317997	0.760946	-2.097682
14	6	0	1.255418	-0.724816	-0.553782
15	1	0	2.163147	-1.248893	-0.854160
16	6	0	1.260725	-0.727880	1.057314
17	1	0	1.276133	-1.761607	1.396935
18	1	0	2.163663	-0.224360	1.396935
19	6	0	0.000000	1.455760	1.057314
20	1	0	-0.887530	1.985967	1.396935
21	1	0	0.887530	1.985967	1.396935
22	6	0	-1.260725	-0.727880	1.057314
23	1	0	-2.163663	-0.224360	1.396935
24	1	0	-1.276133	-1.761607	1.396935
25	6	0	0.000000	0.000000	1.463488
26	1	0	0.000000	0.000000	2.586843

Sum of electronic and zero-point Energies= -390.314906
Sum of electronic and thermal Energies= -390.307097
Sum of electronic and thermal Enthalpies= -390.306153
Sum of electronic and thermal Free Energies= -390.345713

Ada$^+$(II)

1	6	0	0.314931	0.674387	1.251226
2	6	0	1.022614	1.211447	0.000000
3	6	0	0.314931	0.674387	-1.251226
4	6	0	0.314931	-0.885594	-1.279710
5	6	0	0.918052	-1.505591	0.000000
6	6	0	0.314931	-0.885594	1.279710
7	6	0	-1.118600	1.224650	1.279902
8	6	0	0.824703	-2.972029	0.000000
9	6	0	-1.794836	1.313081	0.000000
10	6	0	-1.118600	1.224650	-1.279902
11	1	0	0.823004	1.042593	2.140454
12	1	0	1.013091	2.304059	0.000000
13	1	0	2.072604	0.923393	0.000000
14	1	0	0.823004	1.042593	-2.140454
15	1	0	-0.702534	-1.264050	-1.399295
16	1	0	0.873577	-1.239934	-2.144974
17	1	0	1.993103	-1.260475	0.000000
18	1	0	-0.702534	-1.264050	1.399295
19	1	0	0.873577	-1.239934	2.144974
20	1	0	-1.771878	0.744738	2.019722
21	1	0	-1.114069	2.278397	1.633784
22	1	0	0.803321	-3.530263	-0.925849
23	1	0	0.803321	-3.530263	0.925849
24	1	0	-1.771878	0.744738	-2.019722
25	1	0	-1.114069	2.278397	-1.633784
26	1	0	-2.859986	1.527286	0.000000

Sum of electronic and zero-point Energies= -390.258696
Sum of electronic and thermal Energies= -390.248971
Sum of electronic and thermal Enthalpies= -390.248027
Sum of electronic and thermal Free Energies= -390.293923

Ada$^+$(III)

1	6	0	-0.875727	-0.441729	-1.263459
2	6	0	-1.574718	-0.963874	0.000000
3	6	0	-0.875727	-0.441729	1.263459
4	6	0	0.614068	-0.864384	1.259046
5	6	0	1.246253	-0.390926	0.000000
6	6	0	0.614068	-0.864384	-1.259046
7	6	0	-0.875727	1.110482	-1.267341
8	6	0	2.598656	0.197483	0.000000
9	6	0	-0.260031	1.557518	0.000000
10	6	0	-0.875727	1.110482	1.267341
11	1	0	-1.363514	-0.816966	-2.161164
12	1	0	-2.619773	-0.651210	0.000000
13	1	0	-1.572755	-2.055050	0.000000
14	1	0	-1.363514	-0.816966	2.161164
15	1	0	1.134554	-0.489329	2.138747
16	1	0	0.674932	-1.958519	1.277643
17	1	0	1.134554	-0.489329	-2.138747
18	1	0	0.674932	-1.958519	-1.277643
19	1	0	-0.357442	1.505152	-2.139137
20	1	0	-1.912342	1.464409	-1.306525
21	1	0	-0.357442	1.505152	2.139137
22	1	0	-1.912342	1.464409	1.306525
23	1	0	2.808628	0.777933	0.896849
24	1	0	2.808628	0.777933	-0.896849
25	1	0	3.312028	-0.642910	0.000000
26	1	0	0.498548	2.330189	0.000000

Sum of electronic and zero-point Energies= -390.316951
Sum of electronic and thermal Energies= -390.308028
Sum of electronic and thermal Enthalpies= -390.307084
Sum of electronic and thermal Free Energies= -390.350448
